# A relay-type innate immunity activation strategy involving water-soluble NIR-II AIEgen for boosted tumor photo-immunotherapy

**DOI:** 10.7150/thno.95724

**Published:** 2024-08-05

**Authors:** Shanshan Liu, Yan Sun, Dingyuan Yan, Wen Song, Jiajia Fu, Shanyong Wang, Tianhao Yang, Jun Zhu, Dongxia Zhu, Dong Wang, Feifan Zhou, Ben Zhong Tang

**Affiliations:** 1 State Key Laboratory of Digital Medical Engineering, School of Biomedical Engineering, Hainan University, Sanya 572025, China.; 2 Key Laboratory of Biomedical Engineering of Hainan Province, One Health Institute, Hainan University, Sanya 572025, China.; 3 Center for AIE Research, Shenzhen Key Laboratory of Polymer Science and Technology, Guangdong Research Center for Interfacial Engineering of Functional Materials, College of Materials Science and Engineering, Shenzhen University, Shenzhen 518060, China.; 4 Key Laboratory of Nanobiosensing and Nanobioanalysis at Universities of Jilin Province, Department of Chemistry, Northeast Normal University, Changchun 130024, China.; 5 School of Science and Engineering, Shenzhen Institute of Aggregate Science and Technology, The Chinese University of Hong Kong, Shenzhen, Guangdong 518172, China.

**Keywords:** photo-immunotherapy, aggregation-induced emission, NIR-II fluorescence imaging, relay-type innate immunity activation

## Abstract

**Background:** Effective innate immunity activation could dramatically improve the anti-tumor efficacy and increase the beneficiary population of immunotherapy. However, the anti-tumor effect of unimodal immunotherapy is still not satisfactory.

**Methods:** Herein, a novel relay-type innate immunity activation strategy based on photo-immunotherapy mediated by a water-soluble aggregation-induced emission luminogen, PEG_420_-TQ, with the assistant of toll-like receptor 7 (TLR-7) agonist, imiquimod (R837), was developed and constructed.

**Results:** The strategy could promote tumor cells to undergo immunogenic cell death (ICD) induced by the well-designed PEG_420_-TQ@R837 (PTQ@R) nanoplatform under light irradiation, which in turn enhanced the infiltration of immune cells and the activation of innate immune cells to achieve the first innate immunity activation. The second innate immunity activation was subsequently achieved by drug delivery of R837 *via* apoptotic bodies (ApoBDs), further enhancing the anti-tumor activity of infiltrated immune cells.

**Conclusion:** The strategy ultimately demonstrated robust innate immunity activation and achieved excellent performance against tumor growth and metastasis. The construction of the relay-type innate immunity activation strategy could provide a new idea for the application of immunotherapy in clinical trials.

## Introduction

Cancer metastasis remains one of the most relevant factors in the current failure of cancer treatment 1-3. In recent years, the emergence of immunotherapy has provided a revolutionary protocol for manipulating metastasis 4-7. Current modalities of immune modulation are mainly focused on enhancing T cell immune responses, such as targeting inhibitory pathways through immune checkpoint inhibitors or activating pathways using chimeric antigen receptor T (CAR-T) cells. The treatment, however, elicits a response in only a minority of patients 8-13. Lately, strategies for enhanced immunotherapy based on activating innate immunity have attracted significant attentions 14-16. The activation/maintenance of anti-tumor activity and the long-term immune memory of T lymphocytes depend on the activation of innate immune system, especially on the activation of antigen-presenting cells (APCs) and repolarization of immunosuppressive tumor-associated macrophages (TAMs), in which immunostimulatory phenotype could substantially activate the innate immunity and dramatically increase the beneficiary population of immunotherapy 17-19. Emerging evidence underscores the significant potential of imiquimod (R837), a hydrophobic small-molecule drug, in bolstering innate immunity through activation of the toll-like receptor-7 (TLR-7) pathway 20, 21. This activation not only triggers the maturation of dendritic cells (DCs) but also fosters the pro-inflammatory repolarization of macrophages, thereby enhancing the overall immune response. Despite those progresses, the anti-tumor effect of unimodal immunotherapy is still confronted with the limitations of inadequate immunogenicity, large individual differences in therapeutic responses as well as off-target toxicity 22.

Synergistic cancer therapy is expected to achieve the better therapeutic effect as it could remedy the deficiencies of individual approaches and integrate strengths of multimodal therapies 23-26. Photo-immunotherapy, based on the combination of phototherapy and immunotherapy, has emerged as a promising strategy with non-invasiveness and high spatiotemporal controllability to combat cancer 27-29. Especially, the photo-immunotherapy based on aggregation-induced emission luminogens (AIEgens) has gained increasing attention due to its excellent reactive oxygen species (ROS) generation and photothermal conversion characteristics 30-32. The generated ROS and heat would lead to immunogenic cell death (ICD), exposure of tumor-associated antigens, and release of damage-associated molecular patterns (DAMPs), thereby inducing the infiltration of immune cells at tumor sites and initiating the activation of innate immunity 33-38. In addition, AIEgens with fluorescence emission in the second near-infrared window (NIR-II) are preferable because of the reduced autofluorescence, minimized photodamage and deeper penetration depth, which allows for fluorescence imaging (FLI) of deep located lesions 39-44. Remarkable as it is, the overwhelming majority of NIR-II AIEgens are hydrophobic which require to be encapsulated into water-dispersible nanoparticles for further study. However, the limited photosensitizer-loading amount and complicated formulation procedures may lead to unsatisfactory diagnostic outcomes and low ICD induction efficiency.

Apoptotic bodies (ApoBDs) are membrane-enclosed packets secreted by tumor cells during apoptosis, which are generated by the outward blebbing of the plasma membrane and have the capacity to store remaining drugs 45-48. The apoptotic signals carried by ApoBDs have the potential to facilitate the recognition of ApoBDs by immune cells, including DCs and macrophages, thereby enabling the efficient delivery of the stored drugs 49. Therefore, such a strategy could be established to induce ICD of tumor cells utilizing photo-immunotherapy mediated by the water-soluble NIR-II AIEgen, which promotes the infiltration of innate immune cells. Meanwhile, the innate immune activation drugs delivered *via* ApoBDs derived from apoptotic tumor cells serve to intensify the activity of innate immune cells, ultimately achieving relay-type innate immune activation.

**Figure 1:**
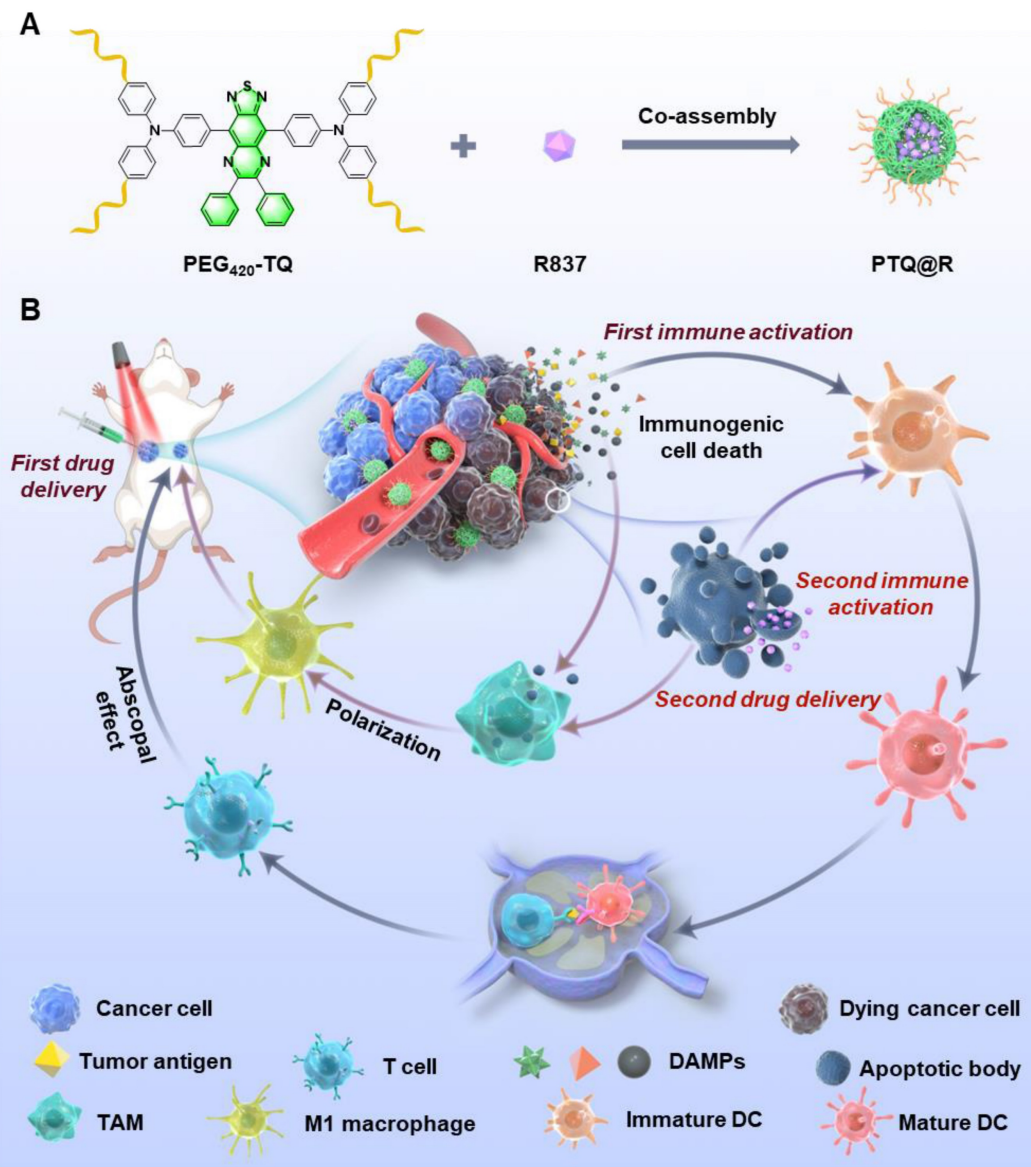
Schematic illustration of nanofabrication and applications. (A) Nanofabrication of PTQ@R. (B) Strategic overview of the relay-type immunity activation strategy induced by PTQ@R for boosted tumor photo-immunotherapy.

We herein report a relay-type innate immunity activation strategy involving a water-soluble NIR-II AIEgen for dramatically boosting tumor therapy. The developed photo-immunotherapy nanoplatform PTQ@R was constructed by the integration of R837 and a water-soluble AIEgen, PEG_420_-TQ, *via* π-π stacking (Figure 1A). The utilization of PTQ@R could achieve twice drug deliveries and immune activations with a single drug administration, significantly enhancing therapeutic efficacy in both primary and distant tumors. As shown in Figure 1B, PTQ@R could effectively accumulate in tumor region for the first drug delivery and perform prominent fluorescence imaging of deep tumor tissues at NIR-II. Furthermore, upon 660 nm laser irradiation at the tumor site, PTQ@R could provide photo-immunotherapy to tumor cells and induce ICD to promote the release of tumor-associated antigens as well as the infiltration and activation of APCs in tumor region (first immune activation). Subsequently, apoptotic tumor cells deliver R837 *via* ApoBDs to the infiltrated APCs in tumor region (the second drug delivery) for further innate immunity activation (the second immune activation). Ultimately, the strategy of the twice drug deliveries can trigger synergistic relay-type innate immunity activation with laser irradiation to efficiently inhibit both the primary and distant tumors, prevent pulmonary metastasis and prolong survival in tumor-bearing mice. The establishment of this nanoplatform offers a novel solution for advancing clinical immunotherapy.

## Results and Discussion

### Synthesis and photophysical studies

**Figure 2:**
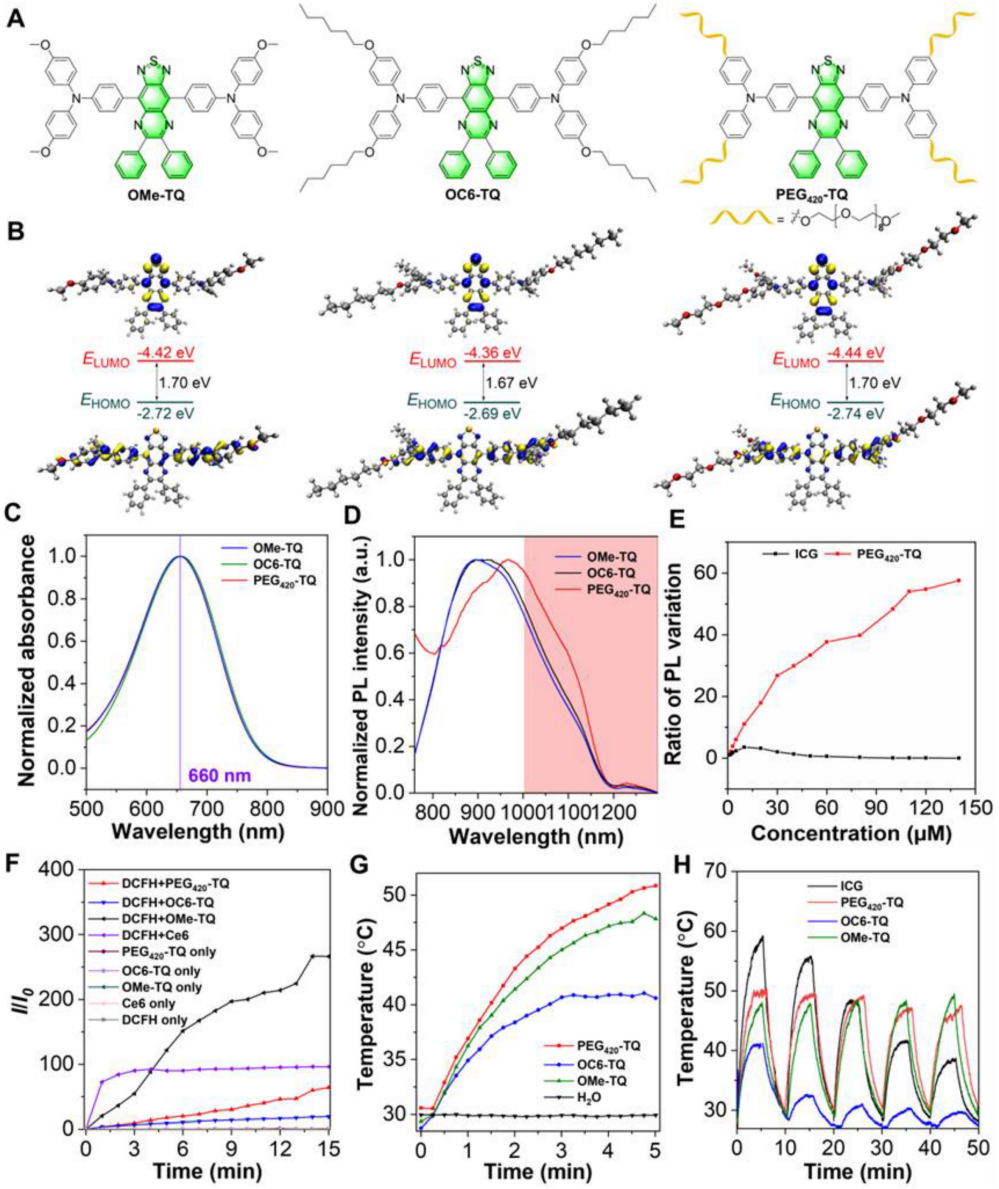
Characterization of obtained compounds. (A) Chemical structures. (B) Optimized S_0_ geometries and illustration of the frontier molecular orbitals (LUMOs and HOMOs) determined at the B3LYP/6-311g(d,p) level of theory. (C) Absorption spectra and (D) normalized PL spectra of three luminogens in THF solution (10 µM). (E) The multiple of emission enhancement of PEG_420_-TQ and ICG at different concentrations. (F) Relative changes in PL intensity of DCFH (for overall ROS detection) in the presence of OMe-DPTQ, OC6-DPTQ, PEG_420_-TQ and Ce6 (1 µM) upon laser irradiation for different times (660 nm, 0.3 W cm^-2^). (G) Photothermal conversion behaviors of OMe-DPTQ, OC6-DPTQ, PEG_420_-TQ (100 µM) under 660 nm laser irradiation (0.3 W cm^-2^). (H) Cyclic temperature curves of OMe-DPTQ, OC6-DPTQ, PEG_420_-TQ and ICG in aqueous solution (100 µM) under 660 nm laser irradiation (0.3 W cm^-2^).

As shown in Scheme S1 and Figures S1-S21, OMe-TQ was obtained *via* simple Suzuki coupling reaction between 4-methoxy-*N*-(4-methoxyphenyl)-*N*-(4-(4,4,5,5-tetramethyl-1,3,2-dioxaborolan-2-yl)phenyl)aniline and 4,7-dibromo-5,6-dinitrobenzo[*c*][1,2,5]thiadiazole. It has been revealed that the prevalence of alkyl chains is beneficial to prevent the intermolecular π-π stacking among fluorophores, thereby boosting the fluorescence intensity in the aggregated state 50, 51. In this context, hexyloxy group was recommended to afford OC6-TQ. To endow luminogen with good water-soluble feature, a long polyethylene glycol (PEG) chain, namely PEG_420_, was employed to replace the hydrophobic hexyloxy moiety to synthesize PEG_420_-TQ (Figure 2A). The density functional theory calculations displayed that the lowest unoccupied molecular orbitals (LUMOs) are mostly localized on the electron-withdrawing acceptor, whereas the highest occupied molecular orbitals (HOMOs) are distributed on the triphenylamine units, revealing the intramolecular charge transfer characteristics of OMe-TQ, OC6-TQ and PEG_420_-TQ (Figure 2B). The band gaps between the HOMO and LUMO in three luminogens show slight differences owing to the similarity of electron donor-acceptor interactions.

The photophysical properties of three compounds were then investigated. As displayed by their absorption spectra in Figure 2C, OMe-TQ, OC6-TQ and PEG_420_-TQ showed almost the same maximum wavelength with peak around 660 nm in tetrahydrofuran (THF) solution. Remarkably, they all exhibited a broad emission spectra ranging from 750 to 1200 nm, which affords the use of NIR-II FLI (Figure 2D). Subsequently, their AIE properties were measured in THF/water binary solutions. As expected, OC6-TQ showed better AIE property, which can be explained by the reduced π-π interactions brought by the hexyloxy chain (Figure S22). It is worth noting that the introduction of PEG chain significantly improves the water solubility of PEG_420_-TQ (Figure S23 and Table S1). The relative quantum yield of PEG_420_-TQ was calculated as 1.4% and 1.6% in THF and water, respectively (Figure S24). As shown in Figure 2E and Figure S25, the fluorescence intensity of PEG_420_-TQ increased gradually accompanying with the increase of concentration. On the contrary, the near-infrared dye indocyanine green (ICG) which was approved by the Food and Drug Administration (FDA) showed obviously quenched emission as the concentration surpassed 5 µM.

Thereafter, the therapeutic capacity of PEG_420_-TQ was investigated. As depicted in Figure 2F and Figure S26, PEG_420_-TQ displayed excellent ROS generation efficiency under the irradiation of a 660 nm laser at a power density of 0.3 W cm^-2^, which can be adopted for conducting photodynamic therapy. Besides, the photothermal conversion ability of OMe-DPTQ, OC6-DPTQ, PEG_420_-TQ were systematically evaluated by recording the temperature profiles under different conditions. PEG_420_-TQ demonstrated the strongest PTT ability (Figure 2G). No surprisingly, an obvious laser power and concentration-dependent photothermal generating were achieved, which make it accessible to afford the temperature as needed (Figure S27). In comparison with ICG, PEG_420_-TQ exhibited overwhelming advantage in terms of photothermal stability after five continuous cycles of heating/cooling administration (Figure 2H). To evaluate the photostability of PEG_420_-TQ, UV-vis absorption spectra of PEG_420_-TQ in water were acquired under irradiation with a 660 nm laser (0.3 W cm^-2^) over various durations. As shown in Figure S28, the absorption intensity of PEG_420_-TQ showed little change after 30 minutes of continuous irradiation.

### ICD mediated by PTQ@R based photo-immunotherapy *in vitro*

**Figure 3:**
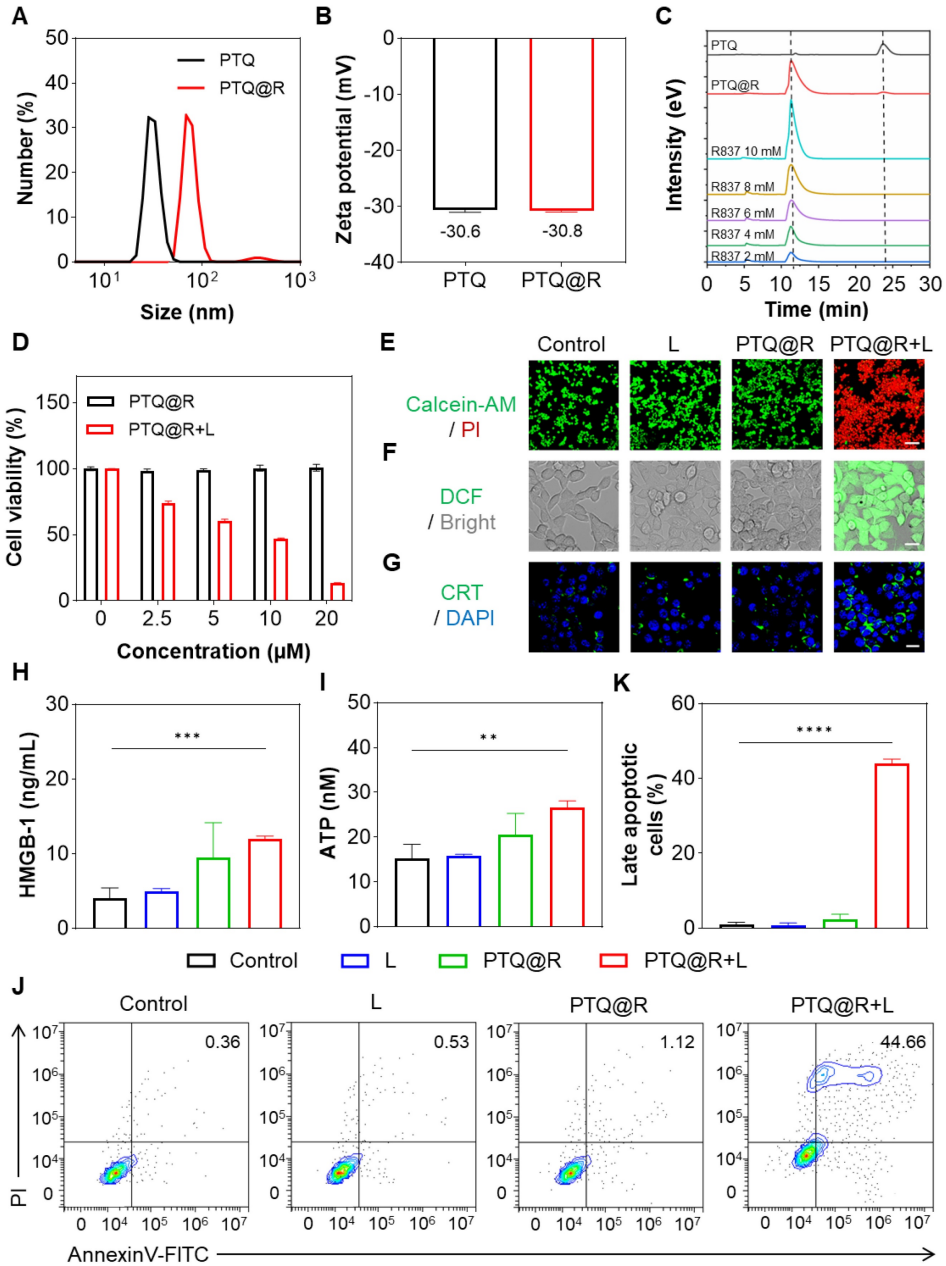
Cell killing and ICD induced by PTQ@R with or without laser irradiation on 4T1 cells. (A) The size distribution of PTQ and PTQ@R determined by dynamic light scattering (DLS). (B) Zeta potential of PTQ and PTQ@R. (C) HPLC analysis of compound PTQ, PTQ@R and R837. (D) Cell viability of 4T1 cells treated with PTQ@R at various concentrations, with or without light irradiation, as determined by the CCK-8 assay. (E) Calcein-AM/PI staining of living/dead 4T1 cells induced by different treatments. Living cells were labeled with Calcein-AM. Dead cells were labeled with PI. Scale bar: 100 μm. (F) Intracellular ROS generation observed by CLSM. Scale bar: 20 μm. (G) Fluorescence images of CRT exposure on 4T1 cell surface. Scale bar: 20 μm. (H) The release of HMGB-1 from 4T1 cells after different treatments. (I) The release of ATP from 4T1 cells after different treatments. (J) Representative flow cytometry plots and (K) quantification of late apoptotic cells induced by different treatments staining with AnnexinV-FITC/PI. **P < 0.01, ***P < 0.001, ****P < 0.0001.

Inspired by the prominent capability of photothermal conversion and ROS production of PEG_420_-TQ (PTQ), we next constructed PTQ@R by introducing R837, a potent TLR-7 agonist, to enhance the tumoricidal effects on tumor cells. As shown in Figure 3A, compared with PTQ, the hydrodynamic size of PTQ@R increased from 28.2 nm to 68.1 nm. Besides, the results of zeta potential exhibited that the surface charge of PTQ@R did not change obviously after loading R837, which suggested that R837 was encapsulated in the core of PTQ *via* π-π stacking (Figure 3B). In order to confirm PTQ@R was successfully constructed, high-performance liquid chromatography (HPLC) analysis was conducted to measure the composition of PTQ@R. The HPLC profile in Figure 3C indicated that the composition of PTQ@R contains PTQ and R837. Besides, the encapsulation efficiency of PTQ@R was determined to be 48.8% using HPLC (Figure S29). Meanwhile, fractions with a retention time of 12.5 min were collected, ^1^H NMR and HRMS further indicated that R837 was encapsulated in PTQ (Figure S30 and Figure S31). Furthermore, the UV-vis-NIR absorption spectra of PTQ and PTQ@R were detected respectively. As illustrated in Figure S32 and Figure S33, compared with PTQ, two characteristic absorption peaks of R837 were observed in PTQ@R at 305 nm and 318 nm, which indicated that R837 was successfully loaded into PTQ and PTQ@R was constructed. Next, the morphology of PTQ@R was observed by TEM. As shown in Figure S34, PTQ@R with uniform size and regular morphology were prepared. Additionally, to evaluate the stability of PTQ@R, the particle size changes of PTQ@R have been determined in H_2_O, phosphate buffered saline (PBS) and fetal bovine serum (FBS) over seven days. As depicted in Figure S35, PTQ@R exhibited excellent stability over a seven-day period, as evidenced by the absence of notable alterations in hydrodynamic size when dispersed in H_2_O, PBS and FBS.

To assess the cytotoxic effects of PTQ@R with or without laser irradiation, the cell viability was evaluated using the cell counting kit-8 (CCK-8) and Calcein-AM/ propidium iodide (PI) staining analysis. As depicted in Figure 3D, PTQ@R displayed no obvious cytotoxic effect on 4T1 cells in the absence of laser irradiation, but exhibited profoundly high cytotoxicity with laser irradiation. At a drug concentration of 20 μM, cell viability in the PTQ@R treated with laser (PTQ@R+L) group was only 13.30%, while the PTQ@R group showed no significant change in cell viability. Calcein-AM/PI staining was then conducted to further explore the tumoricidal efficacy of PTQ@R in the presence of light irradiation (Figure 3E and Figure S36A). As expected, bright green fluorescence could be observed in the Control, L and PTQ@R groups, which indicated that the cells in these three groups were alive. On the contrary, evident red fluorescence from PI was visualized in the PTQ@R+L group, demonstrating that PTQ@R could dramatically promote the death of 4T1 cells under light irradiation. The presented findings indicated that PTQ@R demonstrated favorable biocompatibility in the absence of light, yet exhibited significant cytotoxicity when illuminated. Furthermore, the generation of intracellular ROS was evaluated by using 2',7'-dichlorofluorescein diacetate (DCFH-DA) as a probe. The bright green fluorescence from DCF was clearly visible in the PTQ@R+L group (Figure 3F and Figure S36B). In sharp contrast, the green fluorescence signal was barely observed in PTQ@R group in the absence of light stimulation, indicating that PTQ@R could only generate ROS under light irradiation, which contributes to the high cytotoxicity of PTQ@R+L against tumor cells. These results demonstrated that PTQ@R offered the potential for precise and targeted anti-tumor therapy through the controlled application of light.

DAMPs released by damaged tumor cells, including calreticulin (CRT), high mobility group box-1 (HMGB-1), and adenosine triphosphate (ATP), are considered as the biomarkers of ICD. In this study, the CRT exposure on 4T1 cells were assessed by immunofluorescence and the secretion of HMGB1 and ATP from 4T1 cells were detected by enzyme-linked immune sorbent assay (ELISA). As depicted in Figure 3G and Figure S36C, unlike the faint green fluorescence in other groups, a large amount of green fluorescence was evidently visible in the PTQ@R+L group, which represented that PTQ@R could induce ICD in tumor cells under the exposure of light and promote the expression of CRT to send the "eat me" signal. Additionally, the levels of released HMGB-1 and ATP in the PTQ@R+L group were significantly higher than those in the other groups (Figure 3H and Figure 3I). Based on the presented results, it is evidenced that 4T1 cells treated with PTQ@R+L (the first drug delivery) could release a significant number of DAMPs to effectively induce ICD, which could lead to the activation of immune system (the first immune activation). Subsequently, the apoptosis of tumor cells was further verified by staining with AnnexinV-FITC/PI. As the results of flow cytometry in Figure 3J and Figure 3K indicated, a large proportion of 4T1 cells in PTQ@R+L group were in the late apoptotic state, while the 4T1 cells in the other groups were in the living state. These findings indicated that PTQ@R has the potential to induce ICD in tumor cells and trigger cell apoptosis when exposed to laser irradiation, thereby emerging as a promising candidate for tumor therapy.

### Activation of BMDCs and BMDMs induced by PTQ@R mediated photo-immunotherapy *in vitro*

**Figure 4:**
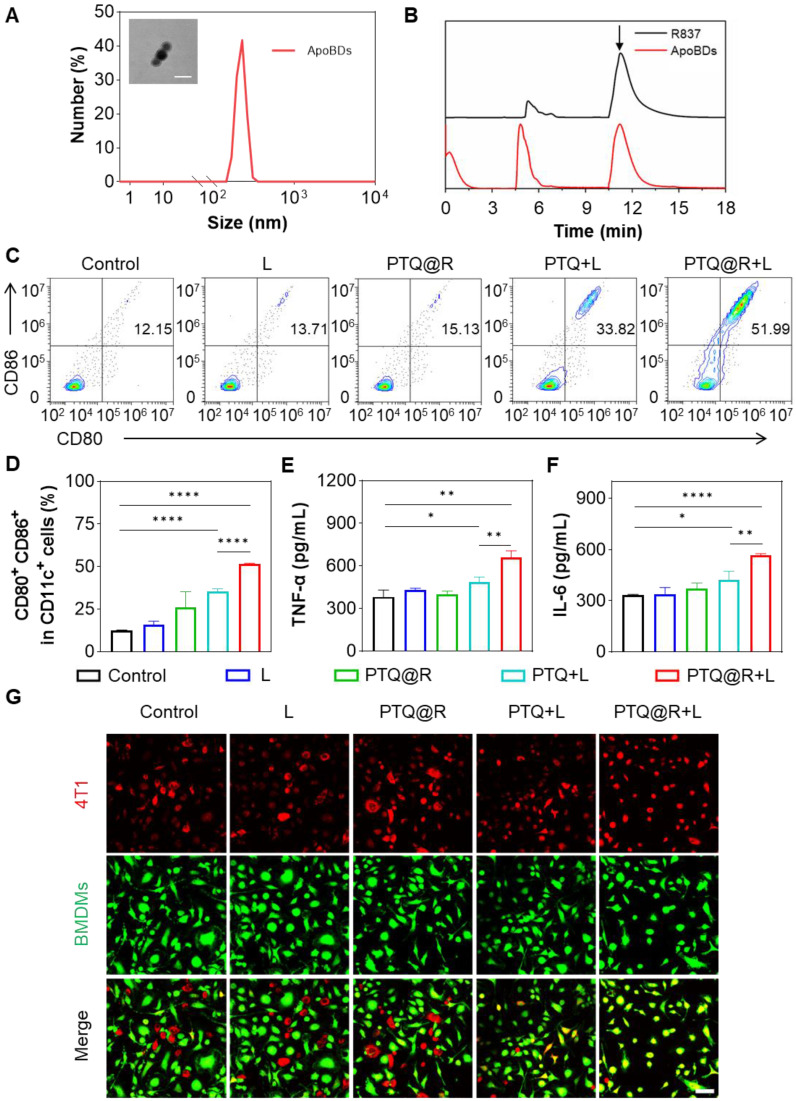
Immune effect induced by PTQ@R with or without laser irradiation on 4T1 cells. (A) The size distribution of ApoBDs determined by DLS. Insert: Representative TEM image of ApoBDs (Scale bar: 100 nm). (B) HPLC analysis of R837 and ApoBDs. The peak of R837 was pointed by arrow. (C) Representative flow cytometry plots and (D) quantification of the expression of CD80 and CD86 (the markers for BMDCs maturation) on the surface of BMDCs stimulated by treated 4T1 cells (gated on CD11c^+^ cells). The secreted cytokines of (E) TNF-α and (F) IL-6 from BMDCs after incubated with treated 4T1 cells. (G) Representative images of phagocytosis of the different treated 4T1 cells by BMDMs. Scale bar: 50 μm. *P < 0.05, **P < 0.01, ****P < 0.0001.

To confirm the second drug delivery of PTQ@R for relay-type immune activation, the ApoBDs produced by apoptotic tumor cells after being treated with PTQ@R+L was obtained by centrifugation. The transmission electron microscope (TEM) image in Figure 4A (insert) revealed that the size of ApoBDs was approximately 80 nm, with a distinct visualization of the membrane structure. Similarly, the hydrodynamic size of ApoBDs was detected by DLS and the result was around 200 nm (Figure 4A). Furthermore, the composition of ApoBDs was solidly confirmed to be R837 through HPLC (Figure 4B).

In order to determine the effect of relay-type innate immunity activation, the maturation of bone marrow-derived dendritic cells (BMDCs) was evaluated as the primary step. BMDCs were isolated from BALB/c mice and the purity of extracted BMDCs was analyzed by flow cytometer (Figure S37). Then, BMDCs were co-cultured with different treated tumor cells. Subsequently, the populations of mature BMDCs were then assessed by flow cytometry. As indicated in Figure 4C and Figure 4D, only 12.50% of mature DCs were identified in the Control group. Similarly, the populations of mature DCs in the L and PTQ@R groups were 15.95% and 25.85%, respectively. These results could be attributed to the fact that the tumor cells treated in Control, L and PTQ@R groups neither undergone ICD nor delivered R837 *via* ApoBDs. However, the proportion of mature DCs in the PTQ@R+L group (51.46%) increased by 4.12-fold compared with the Control group, and 1.45-fold compared with the PTQ+L group (35.52%), providing further evidence that PTQ@R could induce tumor cells to initiate ICD under the light stimulation and achieve relay-type immune activation through the second delivery of R837 by apoptotic tumor cell-derived ApoBDs. Afterwards, the relevant cytokines in the medium of co-cultured DCs with tumor cells were measured by ELISA. As demonstrated in Figure 4E and Figure 4F, the secretion of tumor necrosis factor α (TNF-α) and interleukin 6 (IL-6) in the PTQ@R+L group were much higher than the other groups, which further verified that PTQ@R could promote the maturation of DCs by inducing ICD under light irradiation.

Subsequently, the activation of bone marrow-derived macrophages (BMDMs) after delivery of R837 *via* ApoBDs was explored. BMDMs were isolated from BALB/c mice and the purity of extracted BMDMs was analyzed by flow cytometer (Figure S38). Then, BMDMs were co-incubated with different treated tumor cells. BMDMs and 4T1 cells were labelled with Celltracker Green and Red, respectively. Next, the phagocytosis of 4T1 cells by BMDMs was observed by CLSM. As displayed in Figure 4G and Figure S39, the overlap of red and green fluorescence was barely visible in the Control, L and PTQ@R group, suggesting that BMDMs were immunologically inactive in the untreated tumor microenvironment and therefore do not possess the ability to phagocytose tumor cells. In contrast, yellow fluorescence formed by the overlap of red and green fluorescence was partially available in the PTQ+L group. While a large amount of yellow fluorescence was strongly visualized in the PTQ@R+L group, which fully demonstrated that the PTQ@R+L group could further promote the immune activation of BMDMs by the second delivery of R837 *via* ApoBDs compared to the one-time immune activation by PTQ+L.

### *In vivo* NIR-II fluorescence imaging on 4T1 tumor-bearing mice

In order to verify the fluorescence imaging capability of PTQ@R *in vivo*, the orthotopic 4T1 tumor-bearing mice model was established, which is a triple-negative breast cancer (TNBC) model. After intravenous injection of PTQ@R into 4T1 tumor-bearing mice, the fluorescence of PTQ@R in tumor region was observed by the NIR-II FLI system at different time points. As shown in Figure S40, the fluorescence intensity of PTQ@R increased with time and reached a peak at 24 h post-injection in tumor region (indicated by white circle), which demonstrated that PTQ@R showed good tumor targeting capacity and could achieve NIR-II FLI at tumor region in tumor-bearing mice.

### *In vivo* anti-tumor effect on 4T1 tumor-bearing mice

**Figure 5:**
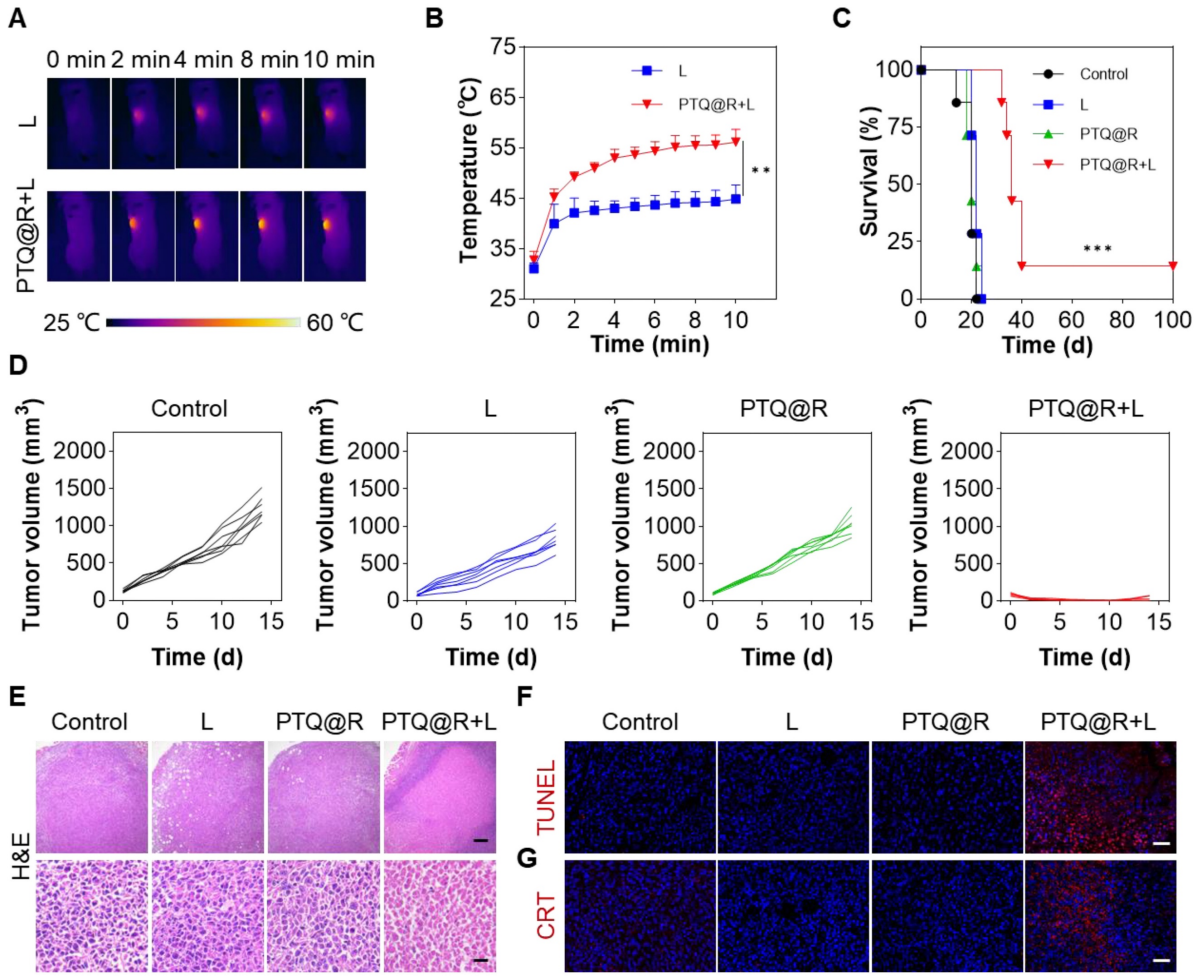
*In vivo* anti-tumor effect on 4T1 tumor-bearing mice. (A) Infrared thermal images of 4T1 tumor-bearing mice and (B) mean temperature of tumors at different monitoring times under laser irradiation (660 nm, 0.8 W cm^-2^, 10 min). (C) Survival curves of mice after different treatments. (D) Individual growth curves of primary tumors after various treatments. (E) Representative images of primary tumors stained with H&E (Upper scale bar: 200 μm. Lower scale bar: 20 μm). Representative images of primary tumors stained with (F) TUNEL and (G) CRT. Scale bar: 30 μm. **P < 0.01, ***P < 0.001.

To investigate the *in vivo* anti-tumor efficacy of PTQ@R on primary tumors, the unilateral 4T1 tumor-bearing mice model was established by subcutaneous inoculation of 4T1 cells (Figure S41). As we know, intratumoral injection is recognized as a highly safe and effective mode of drug delivery for the treatment of subcutaneous tumors, as it offers the advantages of rapidly concentrating the drug at tumor site and minimizing systemic toxicity of the drug. The PTQ@R was initially injected intratumorally, followed by the application of a 660 nm laser on the tumor region, while the temperature changes on the tumor surface was recorded by the infrared thermal imager (Figure 5A). Compared with the L group, the temperature of the tumor injected with PTQ@R increased significantly within 10 min under light irradiation, indicating that PTQ@R also possessed excellent capacity of photothermal conversion *in vivo* (Figure 5B). Then, the unilateral 4T1 tumor-bearing mice were randomly divided into different treatment groups. As shown in Figure 5C, 14% of mice in the PTQ@R+L group survived and remained tumor-free during 100 days after the administration, while mice in Control, L and PTQ@R groups were all dead. Besides, it was observed that the tumor volumes in Control, L and PTQ@R groups gradually increased over fourteen days, whereas the tumor volumes of the mice in PTQ@R+L groups were markedly decreased (Figure 5D and Figure S42). Additionally, no noticeable body weight variation was observed, indicating the treatments had no obvious adverse effects on the mice (Figure S43). In summary, the results showed that PTQ@R+L treatment distinctly suppressed primary tumors growth more than the other groups, and effectively led to better survival rate of tumor-bearing mice.

To further confirm the anti-tumor effect mediated by PTQ@R based photo-immunotherapy, tumors from mice after 1 day of various treatments were collected and analyzed. Hematoxylin and eosin (H&E) and terminal deoxynucleotidyl transferase dUTP nick end labeling (TUNEL) staining of tumor tissues were conducted. In the PTQ@R+L group, extensive destruction of tumor tissues and significant abnormalities in tumor cells were clearly observable, which fully revealed the excellent anti-tumor efficacy after PTQ@R+L treatment (Figure 5E). Meanwhile, an abundance of red fluorescence representing apoptotic tumor cells could be also detected in PTQ@R+L group, further identifying that PTQ@R could tremendously inhibit the growth of primary tumors (Figure 5F and Figure S45A). Subsequently, the exposed CRT in the primary tumor section has also been evaluated. Compared with the other groups, the considerable and marked red fluorescence was presented in the PTQ@R+L group, suggesting PTQ@R+L could effectively induce ICD (Figure 5G and Figure S45B).

**Figure 6:**
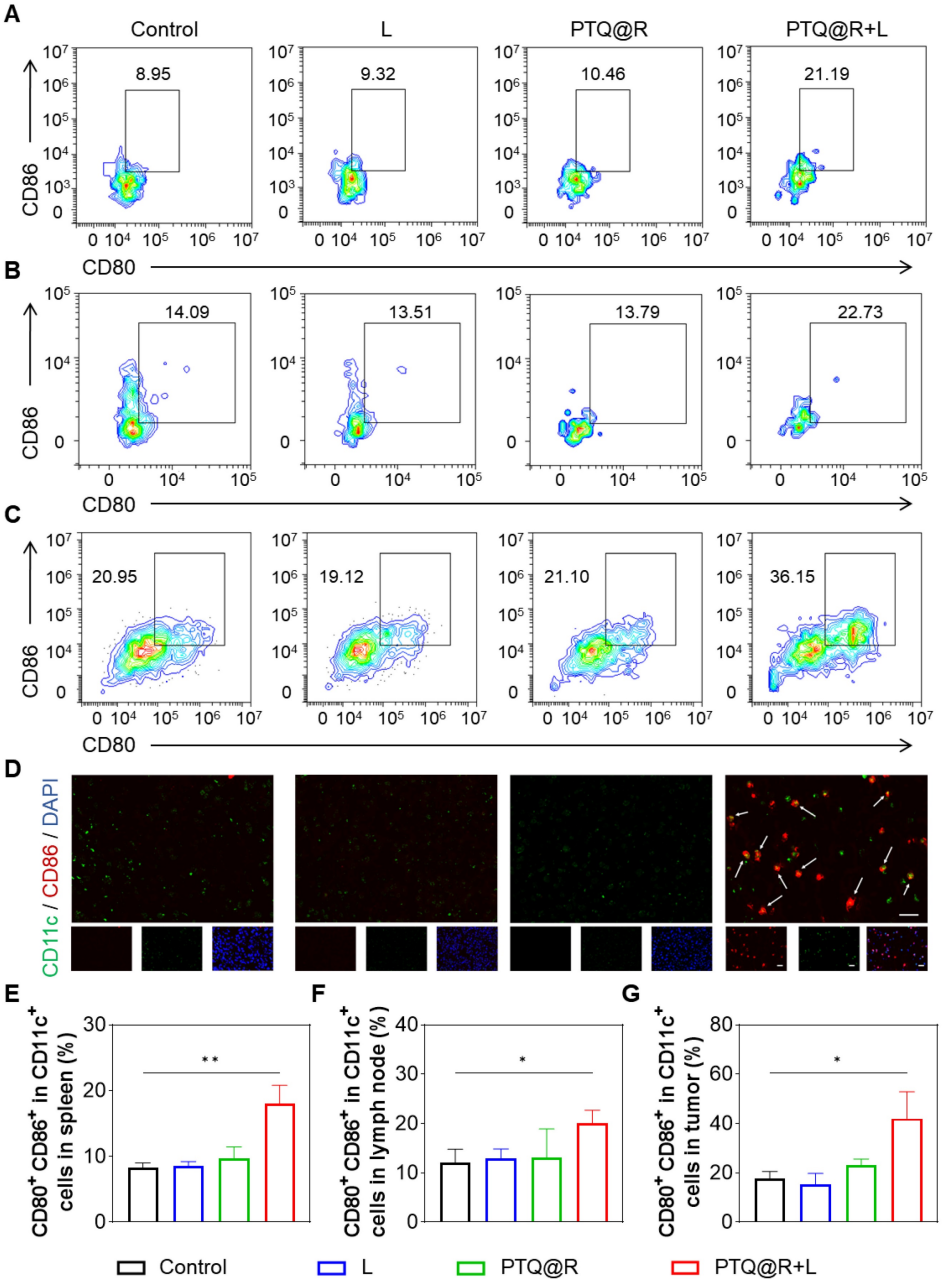
*In vivo* immune response after different treatments. Representative flow cytometric plots of mature DCs (CD11c^+^CD80^+^CD86^+^) in (A) spleens, (B) lymph nodes and (C) primary tumors from mice. (D) Representative images of primary tumors showing DCs stained with CD11c (the marker of DCs) and CD86 (the marker of mature DCs). Scale bar: 20 μm. The mature DCs were pointed by arrows. Quantification of CD11c^+^CD80^+^CD86^+^ in (E) spleens, (F) lymph nodes and (G) primary tumors. *P < 0.05, **P < 0.01.

To evaluate the immune responses after PTQ@R mediated photo-immunotherapy, we investigated the maturation of DCs in spleens and lymph nodes after different treatments. The populations of mature DCs (CD11c^+^CD80^+^CD86^+^) in the spleens and lymph nodes of PTQ@R+L treated mice were significantly higher than those in the Control group (Figures 6A, 6B, 6E, and 6F). Besides, it was also observed that in the L and PTQ@R groups, the populations of mature DCs showed no significant increase after treatments. These results suggest that PTQ@R+L can effectively activate DCs due to the efficient induction of ICD during the first immune activation. Additionally, the activation of DCs in primary tumors was analyzed by flow cytometry and immunofluorescence staining. The population of mature DCs in the primary tumor tissue of the PTQ@R+L group reached up to 42.07%, whereas in the Control, L, and PTQ@R groups, the populations were only 17.56%, 15.20%, and 23.06%, respectively (Figures 6C and 6G). This indicates that PTQ@R+L treatment could induce a stronger antitumor immune response. Immunofluorescence analysis showed similar results, with more CD11c^+^CD86^+^ mature DCs that had infiltrated the PTQ@R+L treated tumors. As shown in Figure 6D, yellow fluorescence, indicating an overlap of red and green fluorescence, was clearly visible within the PTQ@R+L treated tumors. Scarcely any green, red, or yellow fluorescence was observed in Control, L and PTQ@R groups, indicating that these groups had inadequate DCs infiltration and an immunosuppressive tumor microenvironment. Subsequently, the infiltration of CD25-labeled regulatory T cells (Tregs), CD206-labeled M2-phenotype macrophages, and CD49b-labeled natural killer (NK) cells into the primary tumor was also assessed (Figure S44 and Figure S45C-E). A significant decrease in CD25^+^ Tregs and CD206^+^ M2-phenotype macrophages, and an increase in CD49b^+^ NK cells could be observed within the primary tumors treated with PTQ@R+L. These results demonstrate that PTQ@R+L can more effectively induce anti-tumor immune response, alleviate the immunosuppressive microenvironment, and enhance the infiltration of mature DCs into the primary tumors, laying a solid foundation for the subsequent delivery of R837 by ApoBDs as a delivery vector to further activate the immune system.

### *In vivo* immune activation and therapeutic effect

**Figure 7:**
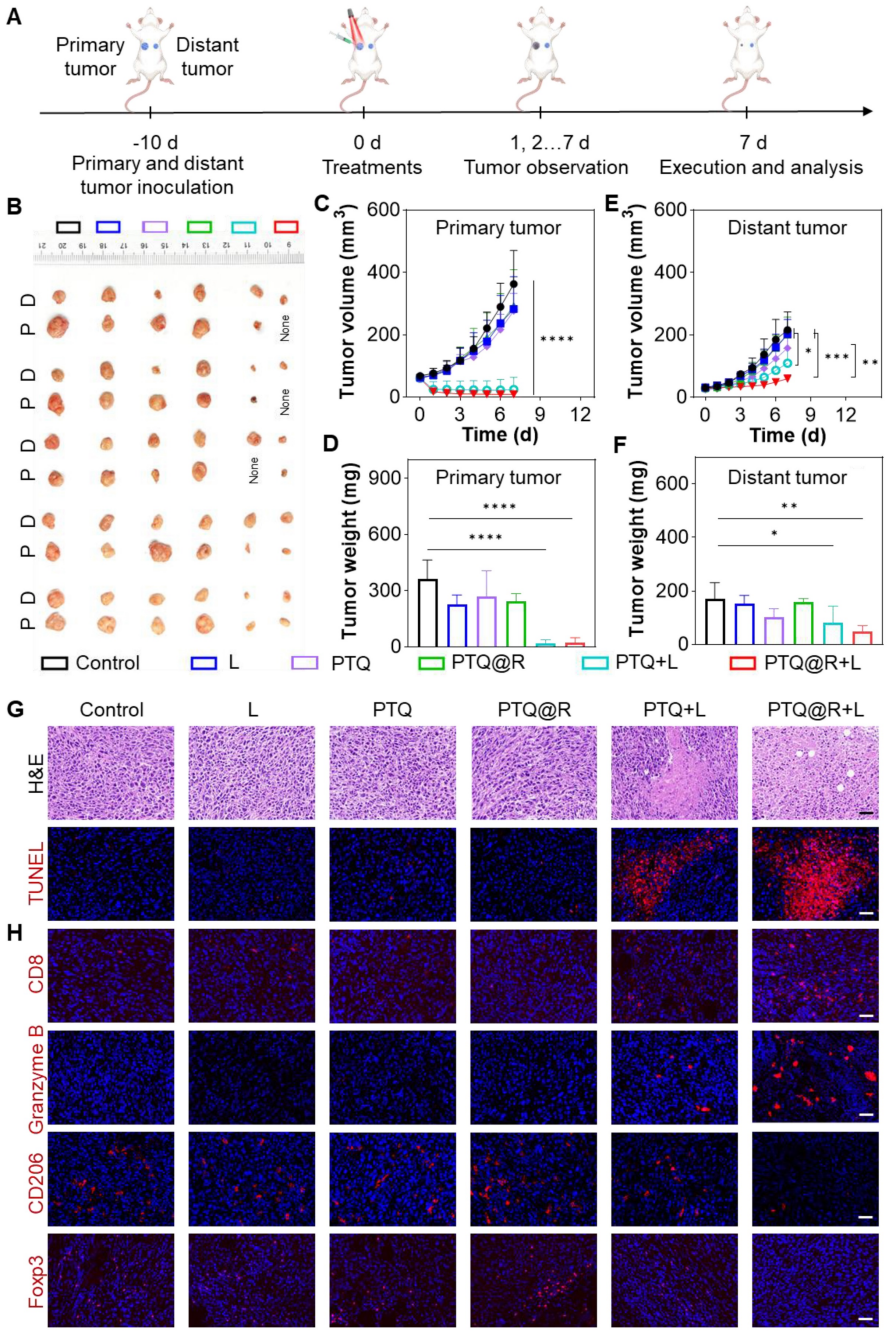
*In vivo* immune activation and therapeutic effect on 4T1 tuomr-bearing mice. (A) Schematic illustration of the *in vivo* anti-tumor therapy. (B) Photographs of dissected primary (indicated as P) and distant (indicated as D) tumors. (C) Tumor growth curves and (D) tumor weights of primary tumors, along with (E) tumor growth curves and (F) tumor weights of distant tumors from mice after various treatments. (G) Representative images of distant tumors stained with H&E and TUNEL after various treatments. Scale bar: 30 μm. (H) Representative images of distant tumors stained with CD8, Granzyme B, CD206 and Foxp3 after various treatments. Scale bar: 30 μm. *P < 0.05, **P < 0.01, ***P < 0.001, ****P < 0.0001.

Further in-depth evaluation of the anti-tumor efficacy of PTQ@R mediated photo-immunotherapy was conducted on bilateral 4T1 tumor-bearing mice model, which was established by subcutaneous inoculation of 4T1 cells onto the right and left flanks of BALB/c mice (Figure 7A). The tumor-bearing mice were sacrificed at the end of treatments. As shown in Figure 7B, the photographs of dissected primary tumors and distant tumors visually illustrated the excellent anti-tumor performance of the PTQ@R+L group. The volume of primary tumors in the PTQ@R+L group was significantly reduced (Figure 7C and Figure S46A) and the primary tumors weight in PTQ@R+L group was distinctly decresed (Figure 7D). In addition, the growth of distant tumors in the PTQ@R+L group was also remarkably inhibited. In comparison with the other groups, the volume (Figure 7E and Figure S46B) and weight (Figure 7F) of distant tumors in the PTQ@R+L group were significantly much smaller. Next, H&E and TUNEL staining of the distant tumors were also implemented. As presented in Figure 7G, a large number of apoptotic and necrotic areas could be clearly observed in the PTQ@R+L group from the H&E staining results, indicating the strategy that combined the photoirradiation of primary tumors with the second delivery of R837 could significantly elevate the systemic immune response and thereby effectively inhibiting the growth of distant tumors. Red fluorescence representing apoptotic cells could be conspicuously detected in TUNEL stained distant tumors sections of the PTQ@R+L group, which further confirmed that the growth of distant tumors in the PTQ@R+L group was effectively inhibited (Figure S47A). Collectively, these results indicate that both primary and distant tumors in the PTQ@R+L group were significantly suppressed, demonstrating the superior anti-tumor efficacy of PTQ@R+L mediated photo-immunotherapy.

Additionally, the extent of immune activation in distant tumors was assessed by immunofluorescence staining. The antibodies of CD8, Granzyme B, CD206 and Foxp3 were used to label cytotoxic T lymphocytes (CTLs), granzyme B, M2-phenotype macrophages and Tregs, respectively. As shown in Figure 7H and Figure S47B-E, in contrast to the results of the other groups, the proportion of CTLs and the production of granzyme B, were significantly elevated in the PTQ@R+L group. Accordingly, the populations of tumorigenic M2-phenotype macrophages as well as immunosuppressive Tregs were significantly declined in the PTQ@R+L group. These results fully illustrated that the relay-type immune activation strategy could fully activate the immune responses, ultimately inhibiting the growth of distant tumors.

**Figure 8:**
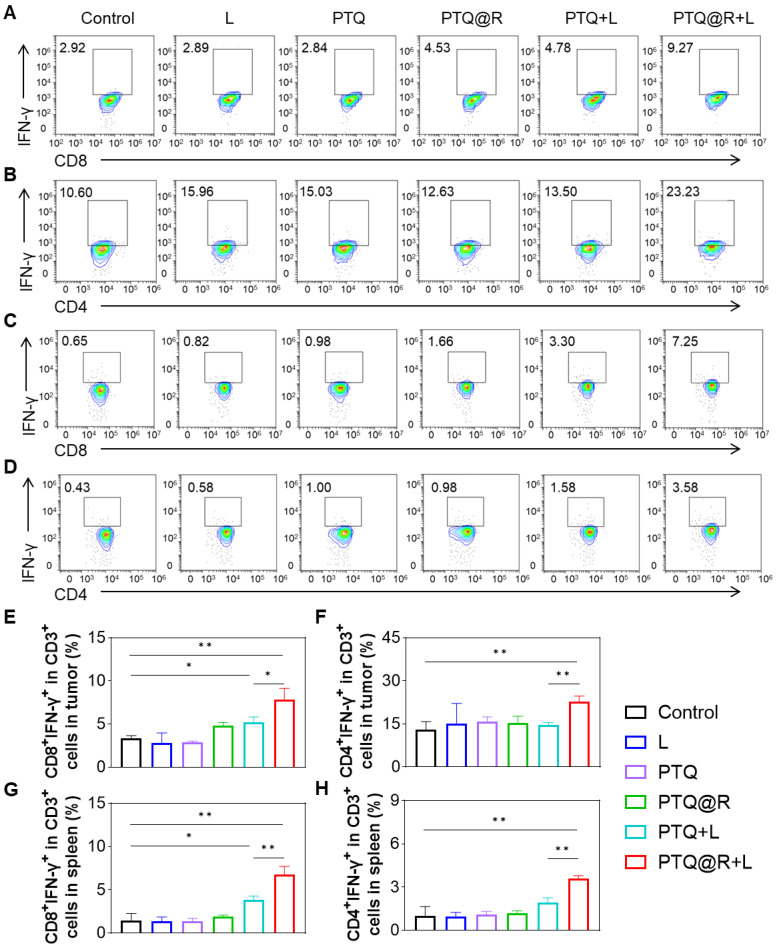
Systemic antitumor immune response induced by PTQ@R mediated relay-type immune activation under laser irradiation. Representative flow cytometry plots of (A) CD3^+^CD8^+^IFN-γ^+^ and (B) CD3^+^CD4^+^IFN-γ^+^ T lymphocytes in distant tumors. Representative flow cytometry plots of (C) CD3^+^CD8^+^IFN-γ^+^ and (D) CD3^+^CD4^+^IFN-γ^+^ T lymphocytes in spleens. Quantification of (E) CD3^+^CD8^+^IFN-γ^+^ and (F) CD3^+^CD4^+^IFN-γ^+^ T lymphocytes in distant tumors. Quantification of (G) CD3^+^CD8^+^IFN-γ^+^ and (H) CD3^+^CD4^+^IFN-γ^+^ T lymphocytes in spleens. *P < 0.05, **P < 0.01.

Next, the populations of T lymphocytes in both distant tumors and spleens from mice in each group were analyzed by flow cytometry (Figure S48). As shown in the Figure 8A and Figure 8B, in the distant tumor tissues, the populations of CD3^+^CD8^+^IFN-γ^+^ and CD3^+^CD4^+^IFN-γ^+^ T lymphocytes in PTQ+L and PTQ@R+L groups were obviously higher than the other groups. It is worth noting that PTQ@R+L induced a greater number of CD3^+^CD8^+^IFN-γ^+^ and CD3^+^CD4^+^IFN-γ^+^ T lymphocytes, specifically 1.51-fold and 1.57-fold when compared to PTQ+L, indicating the presence of effective intratumor lymphocytes infiltration after the second immune activation (Figure 8E and Figure 8F). Similar results could be found in the flow analysis of CD3^+^CD8^+^IFN-γ^+^ and CD3^+^CD4^+^IFN-γ^+^ T lymphocytes in spleens (Figure 8C and Figure 8D). The populations of CD3^+^CD8^+^IFN-γ^+^ and CD3^+^CD4^+^IFN-γ^+^ T lymphocytes in PTQ+L and PTQ@R+L groups were obviously higher than the other groups. Besides, the populations of CD3^+^CD8^+^IFN-γ^+^ and CD3^+^CD4^+^IFN-γ^+^ T lymphocytes in PTQ@R+L were 1.77-fold and 1.85-fold when compared to PTQ+L group (Figure 8G and Figure 8H). Together, these results suggested PTQ@R mediated relay-type photo-immunotherapy synergized the twice immune activation by once drug administration, stimulating superior systemic immune responses over mono-immune activation.

Given that TNBC is one of the most susceptible tumor types to induce lung metastasis, the anti-metastasis potential was assessed. According to the H&E staining results of the lungs, various degrees of pulmonary metastasis were observed in Control, L, PTQ, PTQ@R and PTQ+L groups, while it hard to be found in the PTQ@R+L group (Figure S49A). Meanwhile, the number of pulmonary metastasis nodules from mice in the PTQ@R+L group was significantly less than that in the other groups (Figure S49B). Overall, these results fully manifested the strategy that the twice drug deliveries as well as relay-type immune activation could not only markedly eliminate primary tumors, but also effectively inhibit the growth of distant tumors and suppress the progression of pulmonary metastasis by enhanced systemic anti-tumor effects.

### Biocompatibility

In order to evaluate the toxicity of different treatment in tumor-bearing mice, we recorded the body weight change during treatment period. As shown in Figure S50, no significant weight loss was observed over seven days. After complete treatments, mice were sacrificed, and the major organs and blood were collected for H&E staining and blood biochemistry analysis, respectively. As displayed in Figure S51, no noticeable organ damage or inflammatory lesions were found in the H&E-stained major organs after treatments. Besides, compared with the Control group, the blood biochemistry parameters of hepatic function markers (albumin (ALB), alanine aminotransferase (ALT), aspartate aminotransferase (AST)) and renal function markers (urea (UREA) and creatinine (CREA)) in the PTQ or PTQ@R-administered with or without light irradiation groups appeared to be normal, indicating no obvious systemic side effects of PTQ and PTQ@R (Figure S52).

In additon, to explore the biosafety and systemic toxicity of PTQ@R, we conducted *in vivo* toxicity studies by administering PTQ@R (50 μL, 100 μM PTQ, 1.2 mg kg^-1^ R837) into normal BALB/c mice *via* tail vein. Subsequently, serum biochemistry of the mice was monitored at 5 and 10 days post-injection. As shown in Figure S53A, the blood biochemical markers of mice in all groups exhibited normal ranges. Furthermore, H&E staining of the major organs indicated no significant pathological changes after treatment (Figure S53B). These results suggest that PTQ@R does not exhibit significant systemic toxicity in mice.

Moreover, to confirm the clearance efficiency of PTQ@R *in vivo*, we administered PTQ@R to tumor-bearing mice *via* intratumoral injection, and continuously observed the accumulation of PTQ@R in tumor sites and its clearance *in vivo* using the NIR-II FLI system. As shown in Figure S54, the tumor tissue of mice treated with intratumoral injection exhibited the strongest fluorescence intensity at 0 h, indicating the highest drug content in the tumor region at this time. We observed a gradual decrease in fluorescence intensity in the tumor tissue over time. At 72 h, the fluorescence intensity at the tumor tissue diminished to a level comparable to that observed in the absence of PTQ@R injection, indicating complete drug clearance. Generally, the biocompatibility of PTQ@R is excellent, showing a promising future for antitumor therapy.

## Conclusion

In summary, a water-soluble AIEgen, PEG_420_-TQ, was constructed and synthesized, which displayed favorable capabilities for NIR-II FLI, ROS generation, and photothermal conversion, making it an excellent candidate for anti-tumor therapy. In this study, a strategy based on PEG_420_-TQ with the assistant of immune adjuvant R837 (PTQ@R) for the relay-type innate immunity activation was constructed and developed, in which the first innate immunity activation was achieved by initiating tumor cells to undergo ICD *via* a photo-immunotherapy mediated by PTQ@R, thereby promoting the infiltration of immune cells and the activation of innate immune cells. Subsequently, the second innate immunity activation was achieved by drug delivery of R837 *via* ApoBDs, leading to further activation of the innate immune system, ultimately eliciting systemic anti-tumor immune response. Overall, the strategy significantly facilitated the anti-tumor efficacy and markedly inhibited metastasis. It is promising that the relay-type innate immunity activation strategy offers a great potential for clinical application.

## Supplementary Material

Supplementary experimental section, figures and tables.
