# Supplementary material for: A relay-type innate immunity activation strategy involving water-soluble NIR-II AIEgen for boosted tumor photo-immunotherapy
Source: Theranostics. 2024 Aug 5;14(12):4667–82. doi: 10.7150/thno.95724 (PMC11373630; doi:10.7150/thno.95724)
Supplement: Supplementary file 1 — Supplementary experimental section, figures and tables. [file thnov14p4667s1.pdf]

Supporting Information

A relay-type innate immunity activation strategy involving water-soluble NIR-II AIEgen for boosted tumor photo-immunotherapy

Shanshan Liu [1,2#], Yan Sun [4#], Dingyuan Yan [3*], Wen Song [1,2], Jiajia Fu [1,2], Shanyong Wang [1,2], Tianhao Yang [1,2], Jun Zhu [3], Dongxia Zhu [4], Dong Wang [3*], Feifan Zhou [1,2*], Ben Zhong Tang [5]

[1] State Key Laboratory of Digital Medical Engineering, School of Biomedical Engineering, Hainan University, Sanya 572025, China

[2] Key Laboratory of Biomedical Engineering of Hainan Province, One Health Institute, Hainan University, Sanya 572025, China

[3] Center for AIE Research, Shenzhen Key Laboratory of Polymer Science and Technology, Guangdong Research Center for Interfacial Engineering of Functional Materials, College of Materials Science and Engineering, Shenzhen University, Shenzhen 518060, China

[4] Key Laboratory of Nanobiosensing and Nanobioanalysis at Universities of Jilin Province, Department of Chemistry, Northeast Normal University, Changchun 130024, China

[5] School of Science and Engineering, Shenzhen Institute of Aggregate Science and Technology, The Chinese University of Hong Kong, Shenzhen, Guangdong 518172, China

Corresponding Authors

* Dingyuan Yan - Email: yandingyuan@szu.edu.cn

* Dong Wang - Email: wangd@szu.edu.cn

* Feifan Zhou - Email: zhouff@hainanu.edu.cn

[#] These authors contributed equally to this work

Table of Contents

Experimental Section S4
Synthesis and Characterization S1
Scheme S1. The synthetic routes S14
Figures S1-S2. ^{1}H NMR and ^{13}C NMR spectra of OMe-TQ S22
Figures S3-S4. ^{1}H NMR and ^{13}C NMR spectra of HO-TPA-Br S23
Figures S5-S6. ^{1}H NMR and ^{13}C NMR spectra of C6-TPA-Br S24
Figures S7-S8. ^{1}H NMR and ^{13}C NMR spectra of C6-TPA-NO_2 S25
Figures S9-S10. ^{1}H NMR and ^{13}C NMR spectra of OC6-TQ S26
Figures S11-S12. ^{1}H NMR and ^{13}C NMR spectra of PEG_{420}-OTs S27
Figures S13-S14. ^{1}H NMR and ^{13}C NMR spectra of PEG_{420}-TPA-Br S28
Figures S15-S16. ^{1}H NMR and ^{13}C NMR spectra of PEG_{420}-TPA-NO_2 S29
Figures S17-S18. ^{1}H NMR and ^{13}C NMR spectra of PEG_{420}-TQ S30
Figures S19-S21. HRMS spectra of OMe-TQ, OC6-TQ and PEG_{420}-TQ S31
Figure S22. AIE property of OMe-TQ, OC6-TQ and PEG_{420}-TQ S32
Figure S23. Water solubility study of OC6-TQ and PEG_{420}-TQ S32
Figure S24. NIR-II fluorescence quantum yield measurement of PEG_{420}-TQ S33
Figure S25. PL spectra of PEG_{420}-TQ and ICG at different concentrations S33
Figure S26. ROS generation of OMe-TQ, OC6-TQ and PEG_{420}-TQ S34
Figure S27. Photothermal performance of PEG_{420}-TQ S35
Figure S28. Photostability of PEG_{420}-TQ S35
Figure S29. Standard curve of R837 S36
Figure S30. ^{1}H NMR spectrum of R837 S36
Figure S31. HRMS spectrum of R837 S36
Figure S32. Absorption spectra of PTQ and PTQ@R S37
Figure S33. Absorption spectra of R837 at different concentrations S37
Figure S34. TEM image of PTQ@R S37
Figure S35. The particle size change of PTQ@R S38
Figure S36. Quantitative results of cell death rate, ROS^{+} cells and CRT^{+} cells S38
Figure S37. Flow cytometric analysis of the purity of extracted BMDCs S38
Figure S38. Flow cytometric analysis of the purity of extracted BMDMs S38
Figure S39. Quantitative results of BMDMs phagocytosis of 4T1 cells S39
Figure S40. NIR-II FLI of 4T1 tumor-bearing mice S39
Figure S41. Schematic illustration of treatment of the primary tumor S39
Figure S42. Average primary tumors growth curves of mice S40
Figure S43. Body weight curves of tumor-bearing mice S40
Figure S44. Representative immunofluorescence images of primary tumor stained with CD25, CD206 and CD49b S40
Figure S45. The quantitative results of $TUNEL^{+}$, CRT^{+}, $CD25^{+}$, $CD206^{+}$ and $CD49b^{+}$ cells S41

Figure S46. Individual tumor growth curves of the primary or distant tumors S42
Figure S47. The quantitative results of TUNEL^{+}, CD8^{+} cells, Granzyme B, CD206^{+} cells and Foxp3^{+} T cells S43
Figure S48. The gating strategies for cytometric analysis of DCs and T cells S44
Figure S49. H&E staining of lungs and number of pulmonary metastatic nodules from mice after various treatments S45
Figure S50. Body weight curves of tumor-bearing mice after various treatments S45
Figure S51. H&E images of hearts, livers, spleens and kidneys were harvested from mice at day 7 after various treatments S45
Figure S52. Blood biochemistry analysis after different treatments S46
Figure S53. Biocompatibility analysis after treatments S47
Figure S54. The clearance of PTQ@R post-injection S47
Table S1. The Clog P values of OMe-TQ, OC6-TQ and PEG_{420}-TQ S48
Table S2. Cartesian coordinates for DFT optimized structure (at B3LYP /6-31g (d) level) of OMe-TQ. S48
Table S3. Cartesian coordinates for DFT optimized structure (at B3LYP /6-31g (d) level) of OC6-TQ. S51
Table S4. Cartesian coordinates for DFT optimized structure (at B3LYP /6-31g (d) level) of PEG_{420}-TQ. S57
References S63

Experimental Section

Main materials: Commercially available chemicals, such as nonaethylene glycol monomethyl ether, 1-Bromohexane, *n*-BuLi, tributyltin chloride, $Pd(PPh_3)_4$, benzil and 4,7-dibromo-5,6-dinitrobenzo[*c*][1,2,5]thiadiazole, K_2CO_3, 4-tosyl chloride, *et al.* were obtained from Adamas, Energy, TCI and Bide and used as received unless otherwise stated. Imiquimod (R837) was purchased from MedChemExpress. 2',7′-dichlorofluorescin diacetate (DCFH-DA), Calcein-AM/propidium iodide (PI) detection kit, AnnexinV-FITC/PI detection kit and cell counting kit-8 (CCK-8) were purchased from Beyotime.

Instruments: Reactions were monitored with analytical thin-layer chromatography (TLC) on silica. ^1H NMR and ^{13}C NMR data were recorded on Bruker nuclear resonance (400 MHz, 500 MHz and 600MHz) spectrometers unless otherwise specified, respectively. Chemical shifts (δ) are given in ppm relative to TMS. MALDI-TOF-MS analysis was performed by Bruker Autoflex Speed. UV-vis-NIR absorption spectra were measured on a PerkinElmer Lambda 950 spectrophotometer. PL spectra were recorded on FluoroMax$^+$ fluorescence spectrophotometer of HORIBA. HPLC analyzes and purification were performed on an Acchrom S3000 system with acetonitrile/water as the eluent. Atomic force microscopy (AFM) images were observed by an AFM-Smart SPM (Horiba Jobin Yvon, Inc., France).

Cartesian coordinates of DFT optimized structures: The Gaussian16 suite program using B3LYP/6-31g (d) basis was used to perform geometric optimization and frequency calculation on OMe-TQ, OC6-TQ, and PEG_{420}-TQ the obtained optimized structure had no imaginary frequency, indicating that them were the structure with the lowest energy.

Fluorescence QY measurement: Relative fluorescence quantum yields of PEG_{420}-TQ dissolved in water was measured in a similar way to a previous publication [1]. ICG was employed as the reference (QY = 1.6% in water). For reference calibration, a series of ICG dissolved in water was diluted to its absorbance value of ~0.10, ~0.08, ~0.06, ~0.04, and ~0.02 at 660 nm. The emission was collected in the transmission geometry with a 700 nm long-pass filter to reject the excitation light, and the integrated fluorescence was plotted against absorbance for both reference and samples. The quantum yield was calculated based on the equation as follows:

$$QY_{\mathrm{sample(wavelength)}} = QY_{\mathrm{ref}} \cdot \frac{S_{\mathrm{sample(wavelength)}}}{S_{\mathrm{ref}}} \cdot \left(\frac{n_{\mathrm{sample(wavelenth)}}}{n_{\mathrm{ref}}}\right)^2$$

Where "n" represents the refractive index, "S" represents the slopes obtained by linear fitting of the integrated emission spectra of the samples against the absorbance at 660 nm, respectively.

Photothermal performance measurement: The aqueous solution of OMe-TQ, OC6-TQ, PEG_{420}-TQ and ICG with different concentrations exposed to the 660 nm laser

irradiation with varying power densities for various times. The temperature was recorded 20 times per minute and stopped until the temperature was close to a plateau.

Detection of ROS generation in solution: The generation efficiency of reactive oxygen species (ROS) was commonly measured using DCFH-DA as an indicator. The pre-activation process of the DCFH-DA is as follows: DCFH-DA (0.5 mL) ethanol solution (1×10^{-3} M) was added to NaOH (2 mL) (1×10^{-2} M) and stirred at room temperature for 30 minutes. At that time, DCFH-DA was hydrolyzed to DCFH. The hydrolysate was then neutralized with PBS (10 mL) at pH 7.4 and kept in the dark until use. The activated DCFH-DA solution (DCFH, 4×10^{-5} M) was added to the sample solution containing Me-TQ, OC6-TQ, PEG_{420}-TQ (AIEgen, 1 μM). Then, the mixed solution was irradiated with a 660 nm laser (0.3 W cm^{-2}) for different time intervals. The fluorescence of indicator at 525 nm triggered by AIEgens sensitized ROS was measured by fluorescence spectrometer under 488 nm excitation.

Preparation and characterization of PTQ@R: 1 mg of PEG_{420}-TQ compound and 4 mg R837 (dissolved in DMSO) were poured into 10 mL deionized water, followed by sonication with a microtip probe sonicator at 45% output power for 2 min continuously. Then the mixtures were sealed into dialysis bag and dialyzed against deionized water for 24 h to remove the residual DMSO and unencapsulated R837. The prepared PTQ@R were concentrated to the specified concentrations by ultrafiltration before use.

Hydrodynamic diameter and zeta potential were measured by dynamic light scattering (DLS, Zetasizer Pro, Malvern).

Extraction and characterization of ApoBDs: 4T1 cells were incubated with PTQ@R (PTQ, 20 μM) for 12 h, followed by a 660 nm laser irradiation (0.3 W cm^{-2} for 5 min). The cell supernatant was collected three hours later and centrifuged twice at 50 g for 5 min to remove apoptotic cells and debris. Then, the ApoBDs were harvested by centrifugation at 1000 g for 10 min [2]. The morphology and size of ApoBDs were examined using transmission electron microscopy (TEM, JEM-2100, JEOL).

Cell culture and animal models: The 4T1 murine mammary tumor cells were grown in RPMI 1640 medium (Gibco) supplemented with 10% FBS (Gibco), 50 U/mL penicillin (Gibco) and 50 μg/mL streptomycin (Gibco) in 5% CO_2 at 37 °C in a humidified incubator. Additional granulocyte-macrophage colony-stimulating factor (GM-CSF, PeproTech) was added into the culture medium for BMDCs, or macrophage colony-stimulating factor (M-CSF, PeproTech) was added into the culture medium for BMDMs.

Female BALB/c mice (5-6 weeks old) were purchased from SPF Biotechnology Co. Ltd. All animal experiments were performed in accordance with the Guide for the Care and Use of Laboratory Animals, and obtained approval from Experimental Animal Ethics Committee in Hainan University (HNUAUCC-2021-00025). To establish the

unilateral or bilateral tumor model, 1×10^5 or 2×10^5 4T1 cells were injected into right or bilateral breast of mice. Tumor volume was monitored regularly with Vernier calipers using the formula: length × width^2 × 0.5. The mice were euthanized at the end of treatments or when the tumor burden exceeded 2000 mm^3 in volume, and tumors were collected.

Intracellular ROS generation: 4T1 cells were seeded and cultured in a 24-well culture plate for 12 h. Then, the cells were treated with PTQ@R (PTQ, 20 μM) in fresh medium for another 12 h. Afterwards, cells were stained with DCFH-DA immediately with or without laser irradiation (660 nm, 0.3 W cm^{-2} for 5 min).

Cell death assay: For cytotoxicity and phototoxicity assay, 4T1 cells (1×10^4) were seeded in a 96-well plate per well for 12 h. Then the cells were treated with PTQ@R at indicated concentration for 12h. Cells for phototoxicity assay were irradiated with a 660 nm laser (0.3 W cm^{-2} for 5 min) and incubated for another 12 h. Meanwhile, the cells for cytotoxicity assay were treated with PTQ@R and incubated for 24 h without laser irradiation. After washing, cells were incubated with fresh serum-free medium containing CCK-8 for 2 h in the dark. Then the absorbance was measured at 450 nm with a microplate reader to assess viable percentage of cells. For live/dead cell staining, 4T1 cells (5×10^5) cultured on cell slices in a 24-well culture plate for 12 h and incubated with PTQ@R (PTQ, 20 μM) for another 12 h, followed by a 660 nm laser

irradiation (0.3 W cm^{-2} for 5 min). Cells were costained with Calcein-AM and PI three hours later, then the cell death was observed through confocal laser scanning microscopy (CLSM, FV3000, Olympus). For cell apoptosis detection, cells were stained with Annexin V-FITC/PI and then detected by flow cytometry (Beckman Coulter).

Detection of the immunogenic cell death (ICD) biomarker: 4T1 cells were seeded and cultured in 24-well plate for 12 h, PTQ@R (PTQ: 20 μM, R837: 0.41 mM) was then added into the cell culture medium for another 12 h with or without laser irradiation at 660 nm (0.3 W cm^{-2}) for 5 min. For calreticulin (CRT) expression analysis, cells were fixed in 4% paraformaldehyde after different treatments, and then were incubated with anti-calreticulin antibody (ImmunoWay) for 1 h and the secondary antibody (Invitrogen) for another 1 h at room temperature. After that, cells were stained with DAPI (Invitrogen) for 3 min and imaged by CLSM. For High Mobility Group Box 1 (HMGB-1) and adenosine triphosphate (ATP) released analysis, cell supernatants of different-treated cells were collected after 10 hours post-treatment incubation and extracellular HMGB1 and ATP secreted from treated cells were detected by HMGB-1 enzyme-linked immunosorbent assay (ELISA) kit (mlbio) and ATP ELISA kit (Beyotime), following the manufacturer's protocol.

BMDCs extraction and stimulation: Bone marrow-derived dendritic cells (BMDCs) were generated from BALB/c mice using established methods [3]. The purity of extracted BMDCs was analyzed by flow cytometer. Afterwards, BMDCs were seeded in a 24-well plate and cultured in RPMI 1640 medium for 12 h. Then, BMDCs were incubated with 4T1 cells that had been treated under different conditions (Control, L, PTQ@R, PTQ+L, PTQ@R+L) for 24 h. Subsequently, the cell supernatants of BMDCs were collected, and cytokines secretion from BMDCs were detected using the TNF-α and IL-6 ELISA kit according to the manufacturer's protocol. Furthermore, to evaluate the maturation of BMDCs after co-culture with different treated 4T1 cells, the BMDCs stained with antimouse CD11c, antimouse CD80 and antimouse CD86 were analyzed by flow cytometry.

BMDMs extraction and phagocytosis of 4T1 cells: Bone marrow-derived macrophages (BMDMs) were generated from BALB/c mice using established methods [4]. The purity of extracted BMDMs was analyzed by flow cytometer. Afterwards, BMDMs (2×10^5) were seeded in a 24-well plate and cultured for 24 h. Subsequently, BMDMs and 4T1 cells were stained with Celltracker Green and Celltracker Red (Yeasen), respectively. Fluorescence-labelled BMDMs and 2×10^4 4T1 cells, which had been treated under different conditions (Control, L, PTQ@R, PTQ+L, PTQ@R+L), were co-cultured for 12 h. The phagocytosis of 4T1 cells by BMDMs was then observed using CLSM.

In vivo NIR-II fluorescence imaging: The 4T1 tumor-bearing mice were anesthetized and then PTQ@R (PTQ, 200 μg per mouse) was injected into the mice through the tail vein. *In vivo* NIR-II fluorescence images were collected before and 1, 3, 6, 12 and 24 h after injection on MARS *in vivo* imaging system (Artemis Intelligent Imaging) with the long pass (LP) filter of 1000 nm.

In vivo anti-tumor therapy: When the primary tumor volume achieved approximately 100 mm^3 and the distant tumor volume achieved approximately 30 mm^3, the tumor-bearing mice were randomly divided into different groups. 50 μL PBS or PTQ@R (100 μM PTQ, 1.2 mg kg^{-1} R837) were directly injected into the primary tumors. Then mice were anaesthetized with 2% v/v isoflurane and tumors were irradiated by a 660 nm laser (0.8 W cm^{-2}) for 10 min. The *in vivo* photothermal imaging of mice and surface temperature changes of tumors were recorded by an infrared thermal imaging camera (FOTRIC).

Histological and immunofluorescence assay: Tumors and major organs (heart, liver, spleen, lung and kidney) of mice were excised after being sacrificed for the analysis of anti-tumor effect, immune stimulation and biotoxicity. Tissues were fixed in 4% paraformaldehyde overnight, embedded in paraffin and sectioned at 5 μm thickness. After blocked with bovine serum albumin, the sections were stained with dyes or antibodies: H&E kit (Beyotime), TUNEL kit (Beyotime), CD11c antibody (Invitrogen),

CD86 antibody (Santa Cruz), CD25 antibody (Invitrogen), CD49b antibody (Invitrogen), CD8 antibody (Invitrogen), Granzyme B antibody (Invitrogen), CD206 antibody (Proteintech), Foxp3 antibody (Invitrogen), and 4',6-diamidino-2-phenylindole (DAPI, Invitrogen).

Flow Cytometry: Tumors were harvested, treated with collagenase IV, hyaluronidase and DNase I for 60 min, and grounded using the rubber end of a syringe. Spleens were also harvested and grounded, and red blood cells were removed *via* ACK lysis buffer. The lymph nodes were prepared into a single-cell suspension through grinding. Cells were filtered through nylon mesh filters and washed with PBS. To analyze activated DCs, the cells were stained with anti-mouse CD11c (Biolegend), anti-mouse CD80 (Biolegend) and anti-mouse CD86 (Biolegend) antibodies. To analyze activated T cells, the cells were stained with anti-mouse CD3 (Biolegend), anti-mouse CD4 (Biolegend), anti-mouse CD8 (Biolegend) and anti-mouse IFN-γ (Biolegend).

HPLC analysis of compounds: 2-10 mM solution of R837 was prepared in DMSO, PTQ@R and PEG_{420}-TQ were prepared in H_2O, ApoBDs was prepared in PBS buffer (pH=7.4). For R837, PEG_{420}-TQ, PTQ@R, isocratic elution with 90% phase A (MeCN) and 10% phase B (H_2O) was applied for 30 min, For ApoBDs, same isocratic elution was applied for 18 min. Injection volume was 100 μL. The absorbance wavelength of

HPLC was set to 255 nm and 318 nm. and the percentages of R837 were calculated by the ratio of peak areas at 318 nm.

Statistical analysis: All the data were expressed as the mean ± standard deviations. Statistical analyzes were performed using one-way ANOVA when the compared groups were more than two. For difference analysis between two groups, Student's t-test was used. Significant differences between the groups were expressed as follows: *$P < 0.05$, **$P < 0.01$, ***$P < 0.001$ and ****$P < 0.0001$.

Synthesis and Characterization

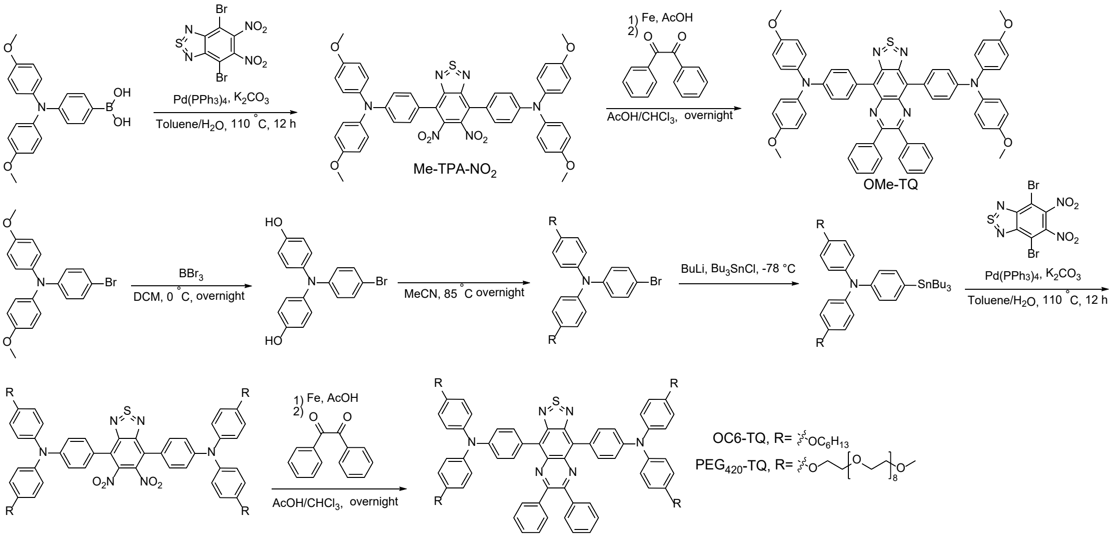

Scheme S1. The synthetic routes of OMe-TQ, OC6-TQ and PEG420-TQ.

General procedure for the synthesis of OMe-TQ, OC6-TQ and PEG420-TQ

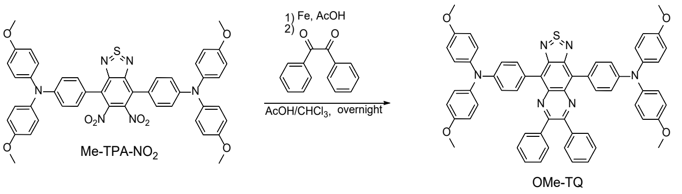

Synthetic route of OMe-TQ: Compound Me-TPA-NO2 was synthesized according to a reported literature [5]. To the mixture of Me-TPA-NO2 (0.1665 g, 0.2 mmol) and acetic acid (5 mL) in a 50 mL two-necked round-bottom flask, iron powder (0.336 g, 6 mmol) was added. The mixture was heated to 80 °C, and stirred for 4 h. After cooling down to room temperature, water was added, and the mixture was washed with dichloromethane three times. The organic phase was combined, dried with Na_2SO_4, and the solvent was evaporated under reduced pressure. The crude product was used without further purification.

The above obtained crude product and benzil (0.044 g, 0.2 mmol) were dissolved in the mixture of acetic acid (2 mL) and chloroform (2 mL) in a 15 mL tube under N_2.

Then the mixture was heated to 80 °C, and stirred for 12 h. After cooling down to room temperature, water was added, and the mixture was extracted with dichloromethane three times. The organic phase was combined, dried with Na_2SO_4, and the solvent was evaporated under reduced pressure. The crude product was purified by column chromatography (PE:DCM = 10:5) to obtain OMe-TQ as a dark green solid (0.160 g, 84%). **^{1}H NMR (500 MHz, Chloroform-*d*)** δ 7.96–7.95 (m, 4H), 7.53–7.50 (m, 4H), 7.20 (s, 2H), 7.12–7.11 (d, *J* = 8.6 Hz, 4H), 6.98–6.94 (m, 8H), 6.86–6.85 (m, 8H), 6.64–6.63 (t, *J* = 8.5 Hz, 4H), 3.78 (s, 12H). **^{13}C NMR (126 MHz, THF-*d_8*)** δ 157.27, 154.95, 153.59, 152.26, 149.82, 140.97, 139.18, 134.85, 132.63, 131.95, 131.75, 127.72, 126.96, 124.31, 118.36, 115.14, 55.19. HRMS (ESI) calculated for $C_{60}H_{47}N_6O_4S$ $[M+H]^+$: 947.3374; Found: 947.3367.

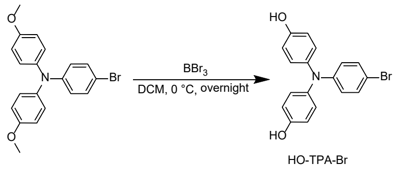

Synthetic route of HO-TPA-Br: To a solution of 4-bromo-*N*,*N*-bis(4-methoxyphenyl)aniline (10 g, 25.8 mmol) in DCM (50 mL), BBr_3 (18.829 g, 76 mmol) was slowly added at 0 °C. The resulting mixture was stirred overnight, water was added to quench the reaction. The mixture was extracted with DCM for three times and dried on anhydrous Na_2SO_4. The crude product was purified with silica gel column chromatography (DCM:MeOH = 20:1) as green solid (8.5 g, 93%). **^{1}H NMR (600 MHz, DMSO-*d_6*)** δ 9.39 (s, 2H), 7.28–7.25 (m, 2H), 6.97–6.93 (m, 4H), 6.77–6.74 (m, 4H),

6.60–6.58 (m, 2H). **^{13}C NMR (151 MHz, DMSO- d_6)** δ 154.92, 148.86, 138.50, 131.93, 127.92, 119.81, 116.79, 110.18.

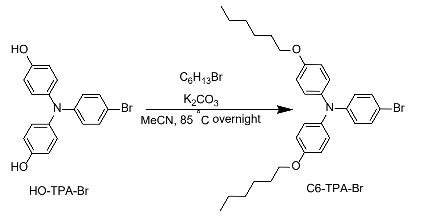

Synthetic route of C6-TPA-Br: To the mixture of HO-TPA-Br (0.1775 g, 1 mmol) and MeCN (20 mL) in a 50 mL two-necked round-bottom flask, 1-Bromohexane (0.4951 g, 3 mmol) and K_2CO_3 (0.2070 g, 1.5 mmol) were added. The mixture was heated to 85 °C and stirred overnight. After the accomplishment of the reaction, the solvent was removed by rotary evaporation. and purified by chromatography (PE: DCM =10:1) to yield the product as a colorless transparent oily liquid (0.245 g, 94%). **^{1}H NMR (500 MHz, THF-d_8)** δ 7.11–7.07 (m, 2H), 6.89–6.85 (m, 4H), 6.72–6.68 (m, 4H), 6.65–6.62 (m, 2H), 3.80 (t, *J* = 6.4 Hz, 4H), 1.65–1.60 (m, 4H), 1.39–1.32 (m, 4H), 1.24 (dq, *J* = 7.2, 3.6 Hz, 8H), 0.83–0.78 (m, 6H). **^{13}C NMR (126 MHz, THF-d_8)** δ 156.03, 148.32, 140.26, 131.49, 126.55, 121.47, 115.11, 111.63, 67.78, 31.62, 29.33, 25.72, 22.58, 13.45.

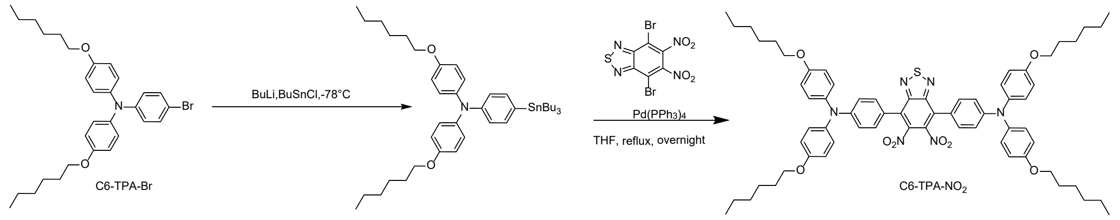

Synthetic route of C6-TPA-NO$_2$: A solution of C6-TPA-Br (0.5256 g, 1 mmol) in dry THF (12 mL) was treated with *n*-BuLi (0.44 mL, 1.1 mmol, 2.5 M in hexane) at -78 °C. After stirring for 1 h, tributyltin chloride (1.1 mmol) was injected at one portion. After

stirring the mixture overnight at room temperature, KF solution was added to quench the reaction. The mixture was extracted with EA for three times, the combined organic phase was dried with Na_2SO_4. After removing the solvent, the product was used directly without further purification.

A 15 mL tube was charged with above obtained crude product, $Pd(PPh_3)_4$ (0.0145g, 0.0125mmol) and 4,7-dibromo-5,6-dinitrobenzo[*c*][1,2,5]thiadiazole (0.144 mg, 0.375 mmol) and degassed dry toluene (5 mL), and sealed with a Teflon cap. The reaction mixture was heated with stirring to 120 °C overnight. Upon cooling, the crude product was quenched with KF solution and extracted with DCM. The combined organic phase was dried with Na_2SO_4. After the solvent was removed, the crude product was purified by column chromatography (PE:DCM = 10:5) to obtain C6-TPA-NO_2 as a purple solid (0.4890 g, 49%). **^{1}H NMR (500 MHz, THF-*d_8*)** δ 7.37–7.33 (m, 4H), 7.17–7.13 (m, 8H), 6.97–6.93 (m, 4H), 6.89–6.85 (m, 8H), 3.94 (t, *J* = 6.5 Hz, 8H), 1.82–1.75 (m, 8H), 1.47 (m, 8H), 1.35 (m, 16H), 0.93–0.89 (m, 12H). **^{13}C NMR (126 MHz, Chloroform-*d*)** δ 156.74, 150.88, 139.50, 130.40, 128.17, 127.98, 117.99, 115.76, 68.53, 31.87, 29.56, 26.02, 22.89, 14.33.

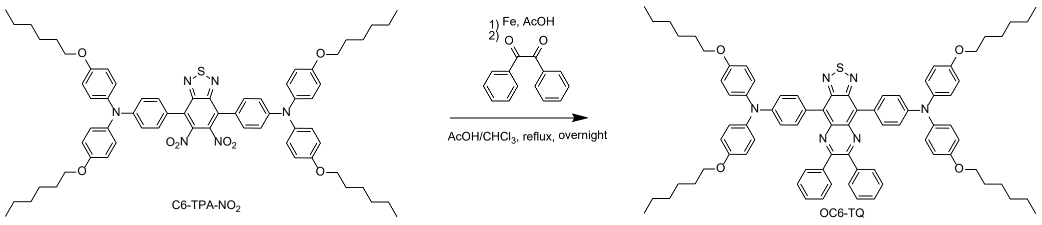

Synthetic route of OC6-TQ: To the mixture of C6-TPA-NO_2 (0.14 g, 0.12 mmol) and

acetic acid (5 mL) in a 50 mL two-necked round-bottom flask, iron powder (0.0211 g, 0.3 mmol) was added. The mixture was heated to 80 °C, and stirred for 4 h. After cooling down to room temperature, water was added, and the mixture was washed with dichloromethane three times. The organic phase was combined, dried with Na_2SO_4, and the solvent was evaporated under reduced pressure. The crude product was used without further purification.

The above obtained crude product and benzil (0.0588 g, 0.24 mmol) were dissolved in the mixture of acetic acid (2 mL) and chloroform (2 mL) in a 15 mL tube under N_2. Then the mixture was heated to 80 °C, and stirred for 12 h. After cooling down to room temperature, water was added, and the mixture was extracted with dichloromethane three times. The organic phase was combined, dried with Na_2SO_4, and the solvent was evaporated under reduced pressure. The product was isolated by silica gel column chromatography (PE: DCM =10:7) to obtain as a OC6-TQ purple as a dark green solid (0.110 g, 75%). **^{1}H NMR (600 Hz, Chloroform-*d*)** δ 7.86–7.84 (m, 4H), 7.58–7.56 (m, 4H), 7.31–7.29 (m, 2H), 7.25 (dd, *J* = 8.1, 6.6 Hz, 4H), 7.14 (d, *J* = 8.4 Hz, 8H), 7.07 (d, *J* = 8.3 Hz, 4H), 6.82–6.80 (m, 8H), 3.90 (t, *J* = 6.6 Hz, 8H), 1.74–1.71 (m, 8H), 1.29 (h, *J* = 3.9 Hz, 18H), 1.18 (s, 6H), 0.86–0.83 (m, 12H). **^{13}C NMR (151 MHz, Chloroform-*d*)** δ 155.83, 153.25, 152.56, 148.93, 140.45, 138.74, 136.00, 133.93, 130.11, 129.40, 128.41, 128.17, 127.30, 126.25, 118.55, 115.32, 68.31, 31.66, 29.39, 25.8, 22.66, 14.10. HRMS (ESI) calculated for $C_{60}H_{47}N_6O_4S$ $[M+H]^+$: 1227.6504; Found: 1227.6495.

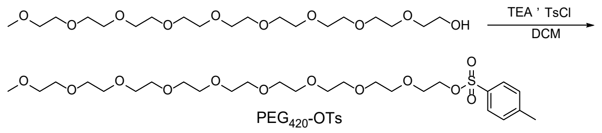

Synthetic route of PEG$_{420}$*-OTs*: To a solution of nonaethylene glycol monomethyl ether (10 g, 23.3 mmol) in dry DCM (50 mL), triethylamine (11.61 ml, 27.96 mmol) was added at 0 °C. After stirring the resulting mixture for 2 h, 4-tosyl chloride (4.45 g, 23.3 mmol) was added and continued to react for 18 h. After accomplishment of reaction, water was added, and the mixture was washed with dichloromethane three times. The organic phase was combined, dried with Na_2SO_4, and the solvent was evaporated under reduced pressure. The product was isolated by silica gel column chromatography (DCM: MeOH=20:1) as colorless transparent oil liquid (12 g, 88%). **^{1}H NMR (400 MHz, Chloroform-*d*)** δ 7.79 (d, *J* = 8.2 Hz, 2H), 7.36 (d, *J* = 8.1 Hz, 2H), 4.17–4.13 (m, 2H), 3.69–3.53 (m, 34H), 3.37 (s, 3H), 2.45 (s, 3H). **^{13}C NMR (101 MHz, Chloroform-*d*)** δ 144.52, 132.65, 129.56, 127.65, 71.60, 70.39, 70.27, 70.23, 70.17, 69.00, 68.33, 58.70, 21.34.

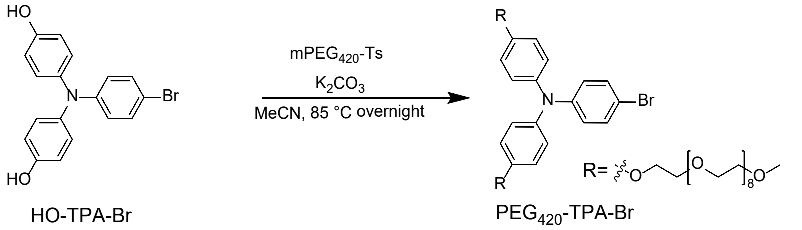

Synthetic route of PEG$_{420}$*-TPA-Br*: The synthesis step of PEG$_{420}$-TPA-Br was similar to C6-TPA-Br. Purification was conducted by chromatography (DCM: MeOH=15:1) to obtain the product as brown oil liquid (3 g, 91%). **^{1}H NMR (500 MHz, Chloroform-*d*)** δ 7.18–7.14 (m, 2H), 6.94–6.90 (m, 4H), 6.77–6.74 (m, 4H), 6.71 (d, *J* = 8.9 Hz, 2H),

4.03 (dd, J = 5.7, 4.0 Hz, 4H), 3.77 (dd, J = 5.6, 4.1 Hz, 4H), 3.66–3.64 (m, 4H), 3.59–3.57 (m, 56H), 3.48 (dd, J = 3.9, 2.0 Hz, 4H), 3.31 (d, J = 1.8 Hz, 6H). **^{13}C NMR (126 MHz, Chloroform-*d*)** δ 155.22, 147.87, 140.69, 131.76, 126.43, 122.14, 115.52, 112.44, 71.94, 70.82, 70.64, 70.58, 70.52, 69.76, 67.69, 59.04.

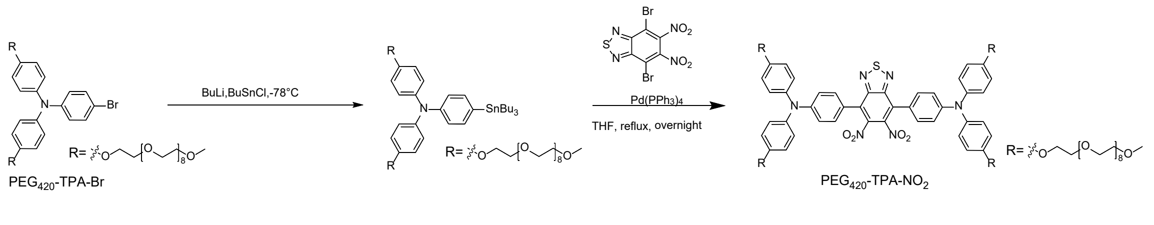

Synthetic route of PEG420-TPA-NO2: The synthesis step of PEG420-TPA-NO2 was similar to C6-TPA-NO2. The crude mixture was purified by chromatography (DCM:MeOH = 12:1) to obtain a purple oil liquid (0.42 g, 56%). **^{1}H NMR (600 MHz, Chloroform-*d*)** δ 7.37 (dt, J = 8.9, 2.8 Hz, 4H), 7.16 (dt, J = 8.9, 2.9 Hz, 8H), 6.96 (dt, J = 8.9, 2.8 Hz, 4H), 6.91 (dq, J = 9.1, 3.2 Hz, 8H), 4.14 (p, J = 3.0 Hz, 8H), 3.87 (dt, J = 6.0, 3.2 Hz, 8H), 3.75 (dt, J = 5.7, 3.2 Hz, 8H), 3.66 (dd, J = 6.9, 3.5 Hz, 112H), 3.55 (dd, J = 6.4, 3.2 Hz, 8H), 3.39–3.37 (m, 12H). **^{13}C NMR (151 MHz, Chloroform-*d*)** δ 156.04, 153.28, 150.46, 142.09, 139.58, 130.14, 127.81, 127.71, 120.56, 117.91, 115.66, 71.90, 70.81, 70.61, 70.53, 70.47, 69.72, 67.67, 59.03.

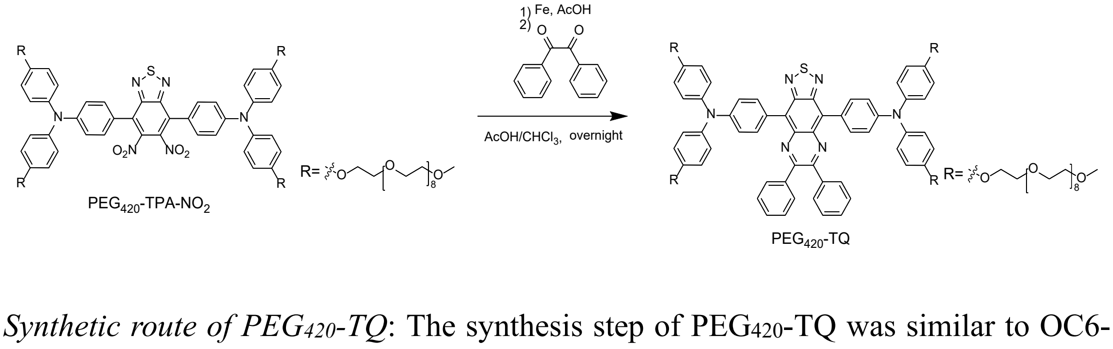

Synthetic route of PEG420-TQ: The synthesis step of PEG420-TQ was similar to OC6-TQ. Purified with silica gel column chromatography (DCM:MeOH = 12:1) to obtain a

dark green oil liquid (0.33 g, 71%). **^{1}H NMR (600 MHz, Chloroform-*d*)** δ 7.95–7.91 (m, 4H), 7.67–7.63 (m, 4H), 7.38 (d, *J* = 7.4 Hz, 2H), 7.33 (dd, *J* = 8.3, 6.8 Hz, 4H), 7.22–7.17 (m, 8H), 7.16–7.13 (m, 4H), 6.92–6.88 (m, 8H), 4.15 (t, *J* = 4.9 Hz, 8H), 3.88 (t, *J* = 4.9 Hz, 8H), 3.75 (dd, *J* = 5.9, 3.6 Hz, 8H), 3.69–3.63 (m, 112H), 3.56–3.54 (m, 8H), 3.37 (s, 12H). **^{13}C NMR (151 MHz, Chloroform-*d*)** δ 155.34, 153.16, 152.60, 148.77, 140.72, 138.61, 135.92, 133.91, 130.04, 129.44, 128.35, 128.16, 127.14, 126.35, 118.68, 115.45, 71.89, 70.79, 70.59, 70.52, 70.46, 69.77, 67.67, 59.02. HRMS (ESI) calculated for $C_{132}H_{191}N_6O_{40}S$ $[M+H]^+$: 2533.2844; Found: 2533.2836.

NMR and MS spectra of compounds

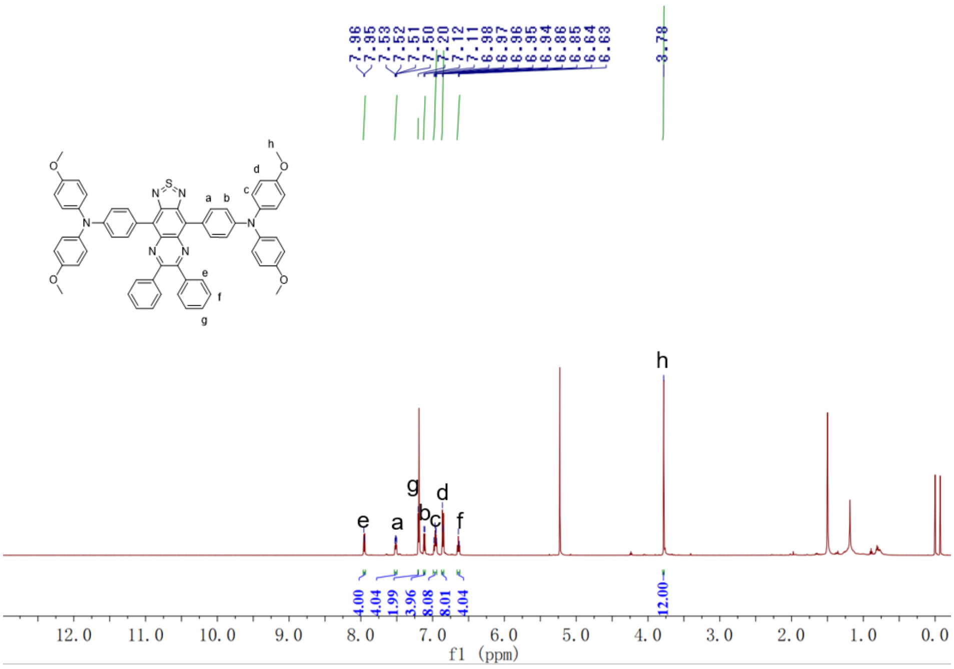

Figure S1. ^{1}H NMR spectrum of OMe-TQ.

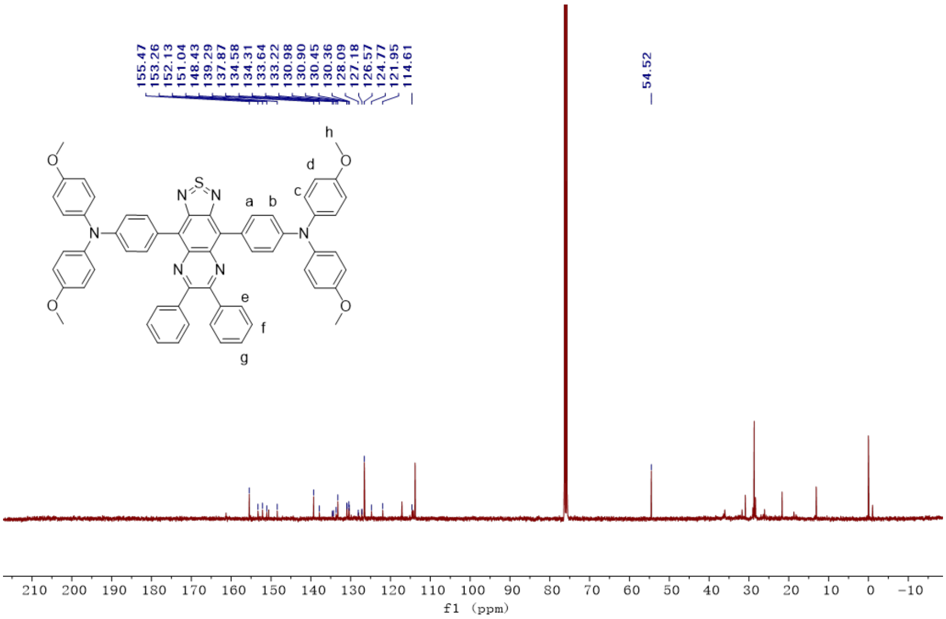

Figure S2. ^{13}C NMR spectrum of OMe-TQ.

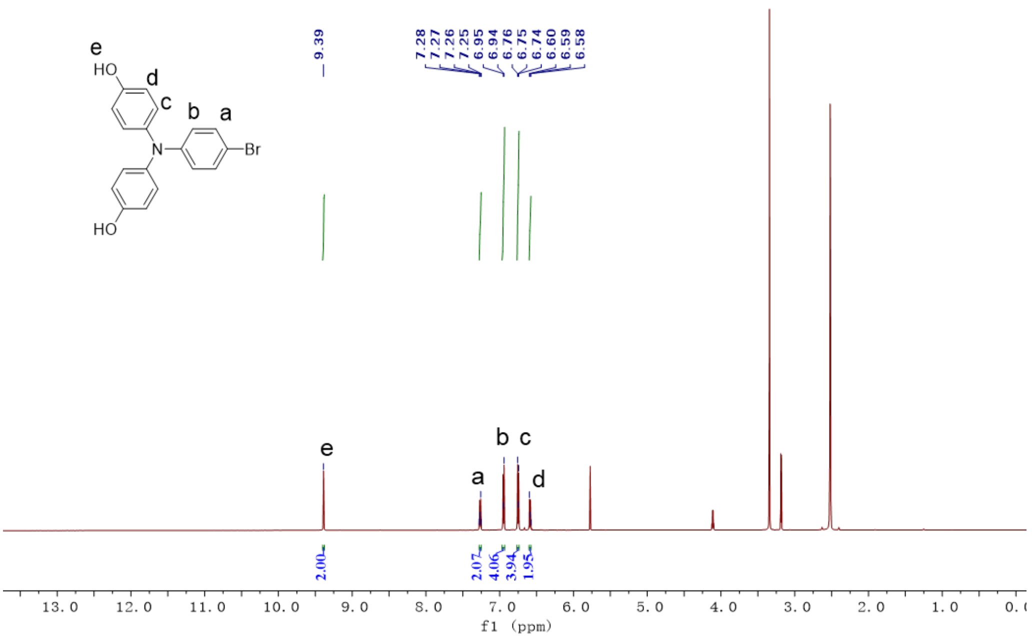

Figure S3. ^{1}H NMR spectrum of HO-TPA-Br.

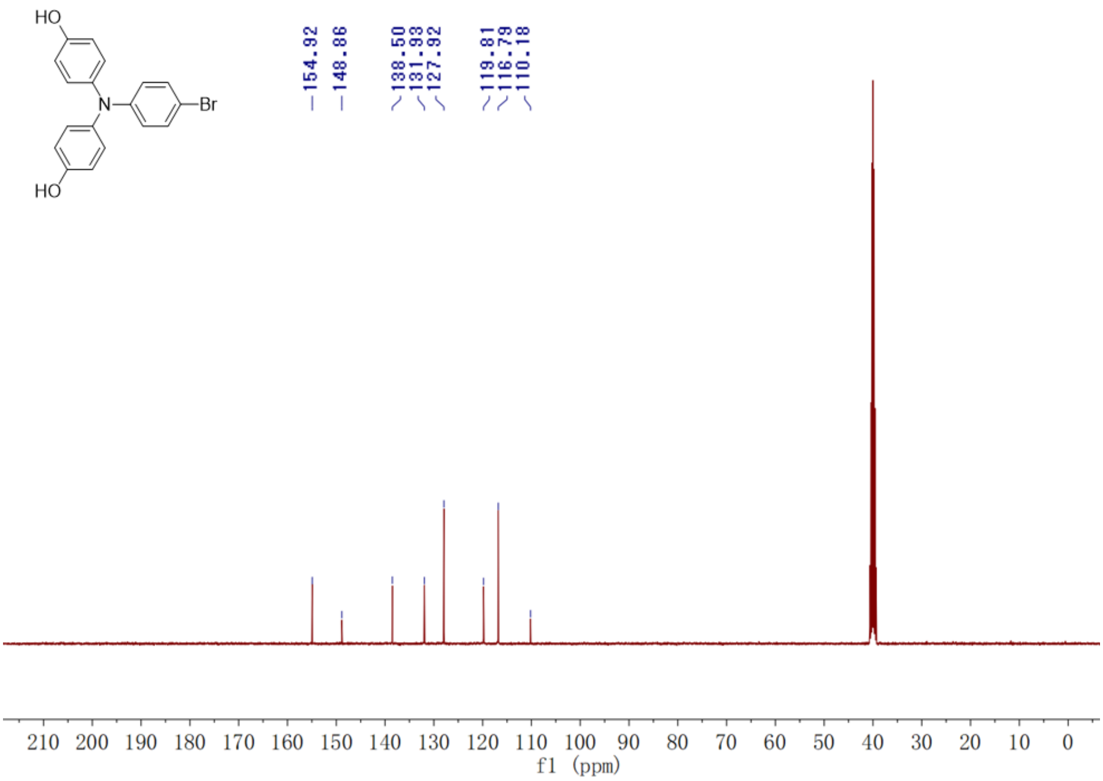

Figure S4. ^{13}C NMR spectrum of HO-TPA-Br.

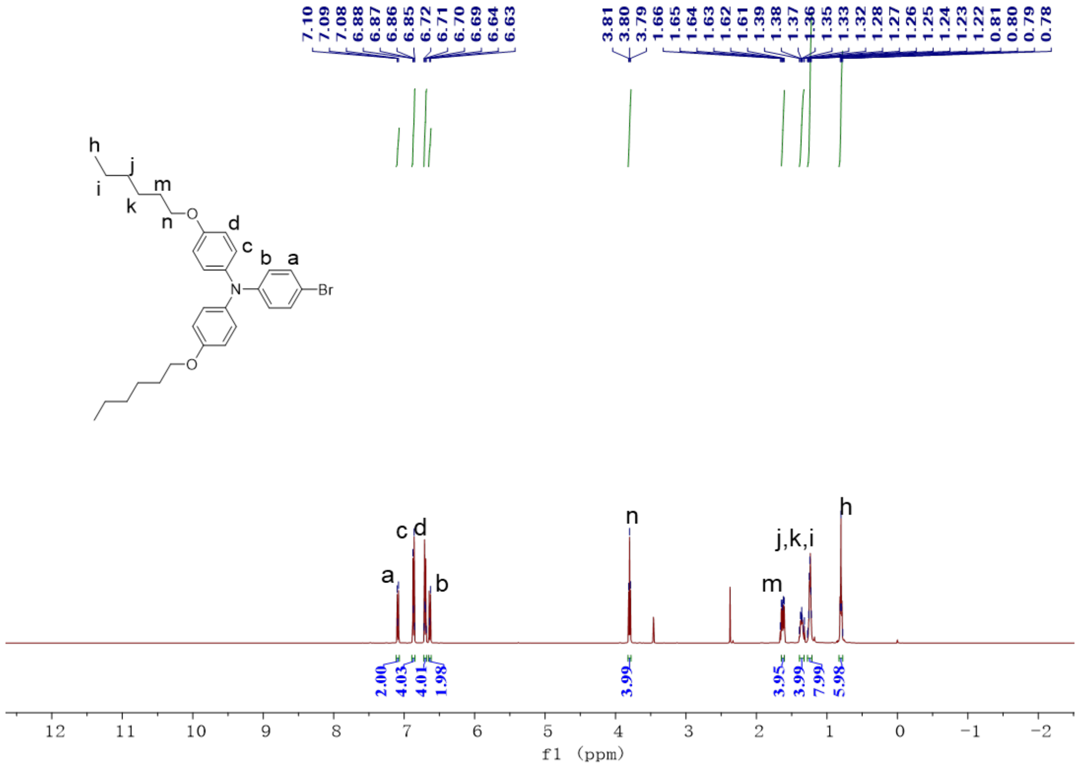

Figure S5. ^{1}H NMR spectrum of C6-TPA-Br.

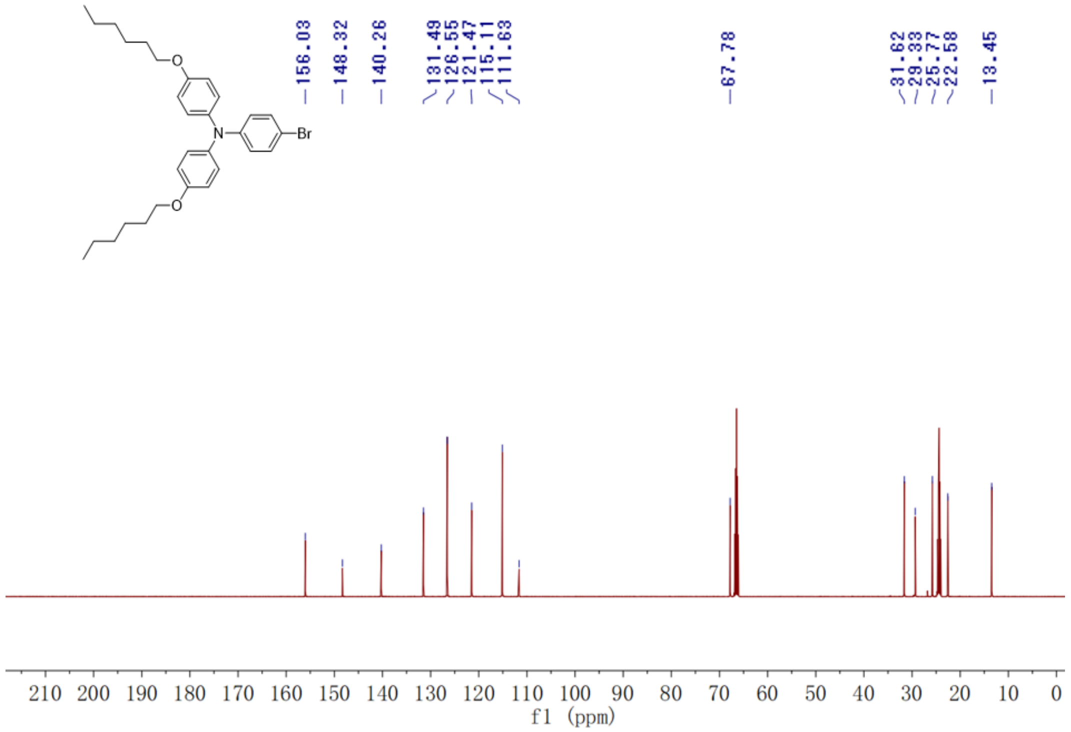

Figure S6. ^{13}C NMR spectrum of C6-TPA-Br.

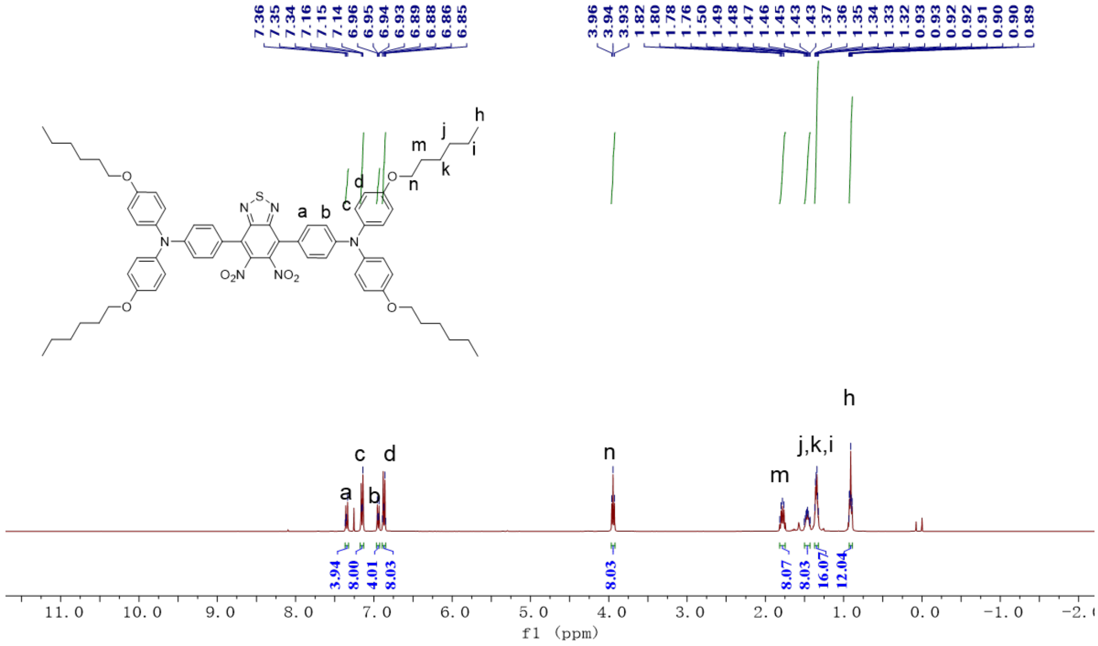

Figure S7. ^{1}H NMR spectrum of C6-TPA-NO_2.

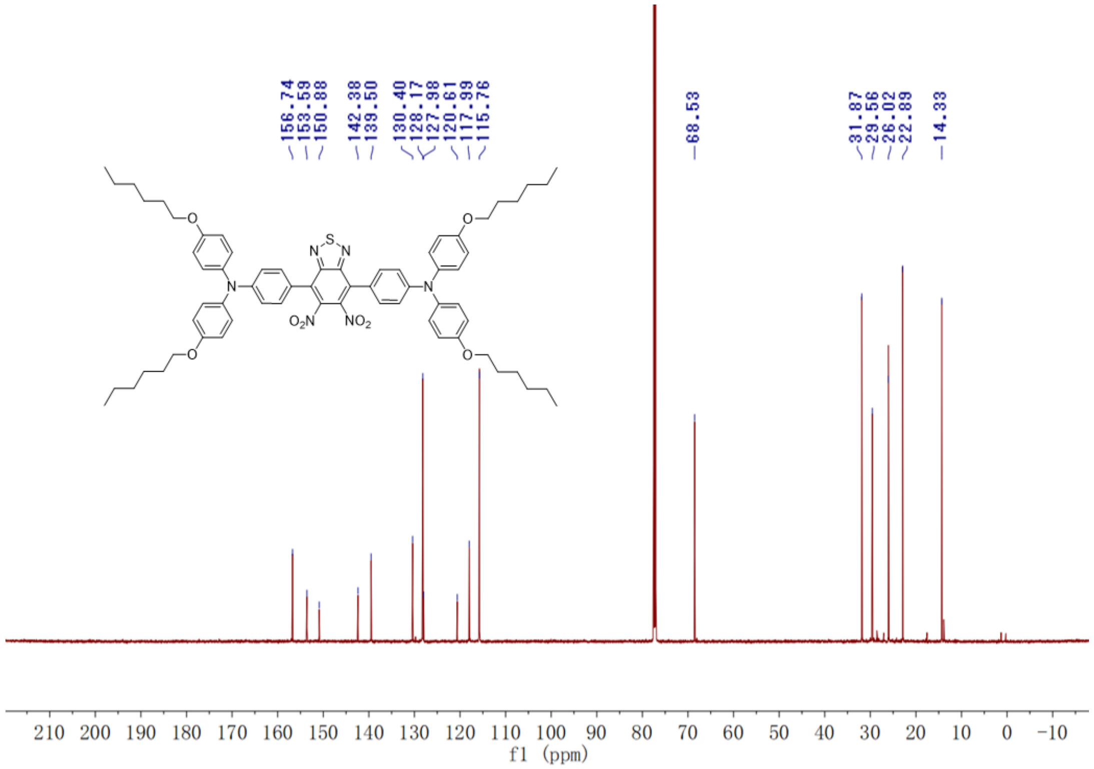

Figure S8. ^{13}C NMR spectrum of C6-TPA-NO_2.

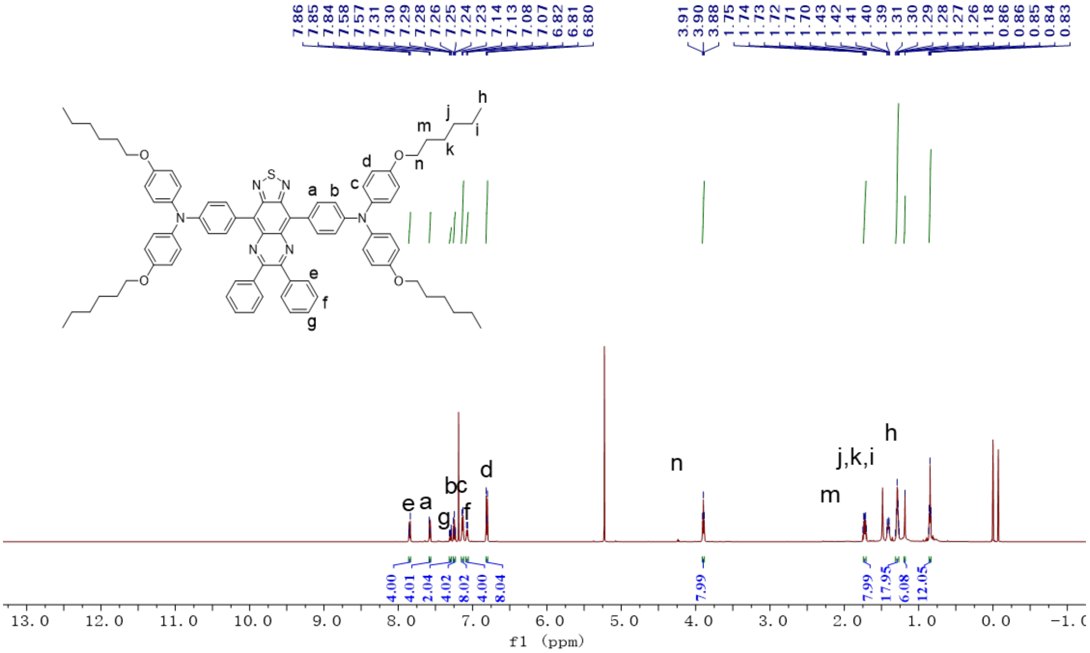

Figure S9. ^{1}H NMR spectrum of OC6-TQ.

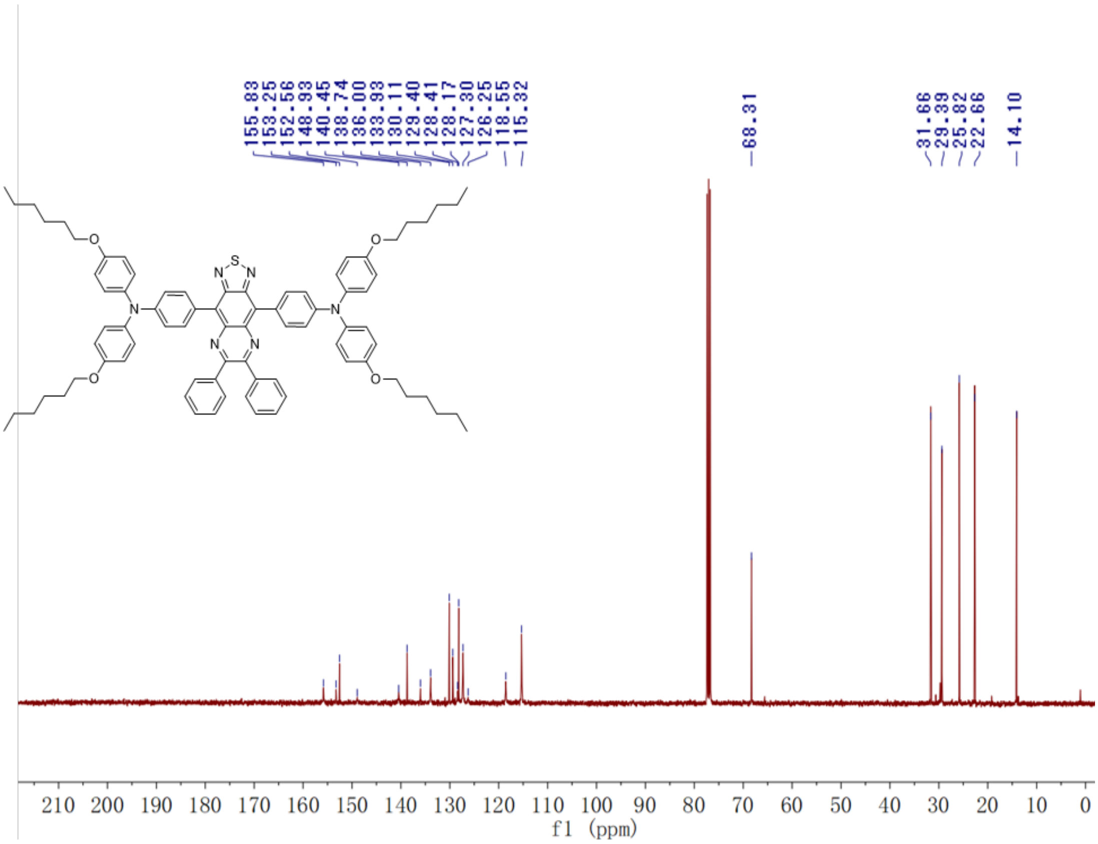

Figure S10. ^{13}C NMR spectrum of OC6-TQ.

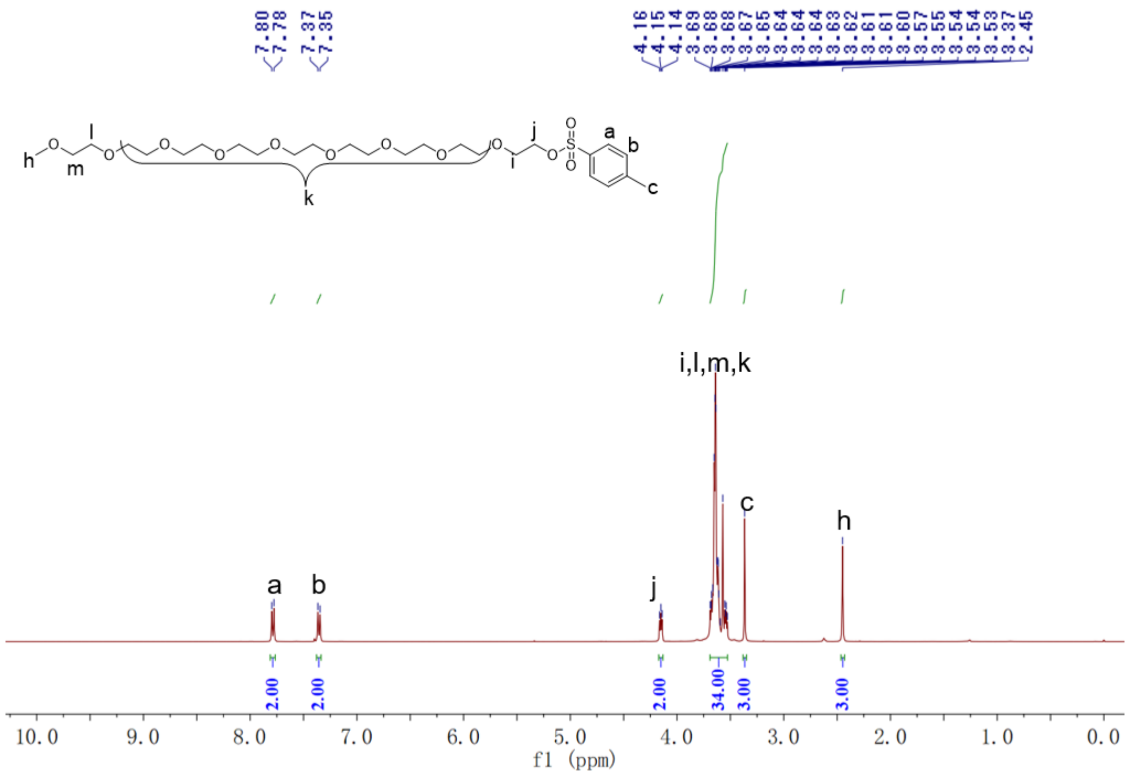

Figure S11. ^{1}H NMR spectrum of PEG420-OTs.

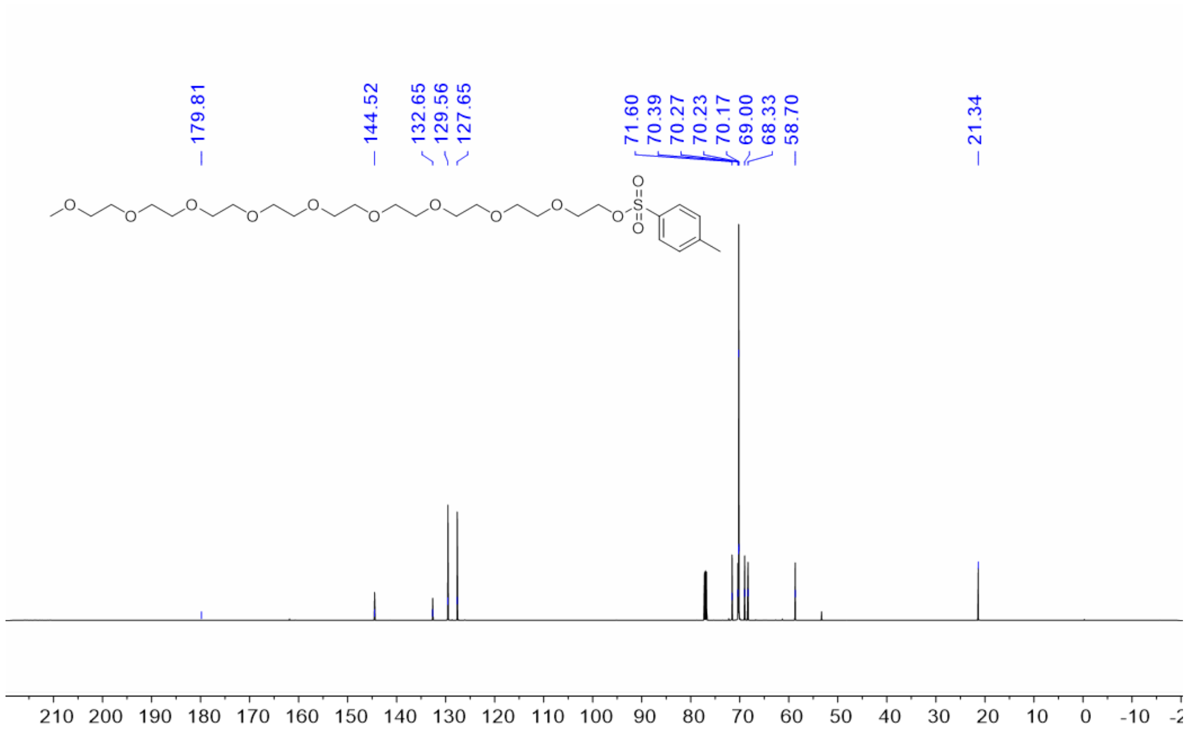

Figure S12. ^{13}C NMR spectrum of PEG420-OTs.

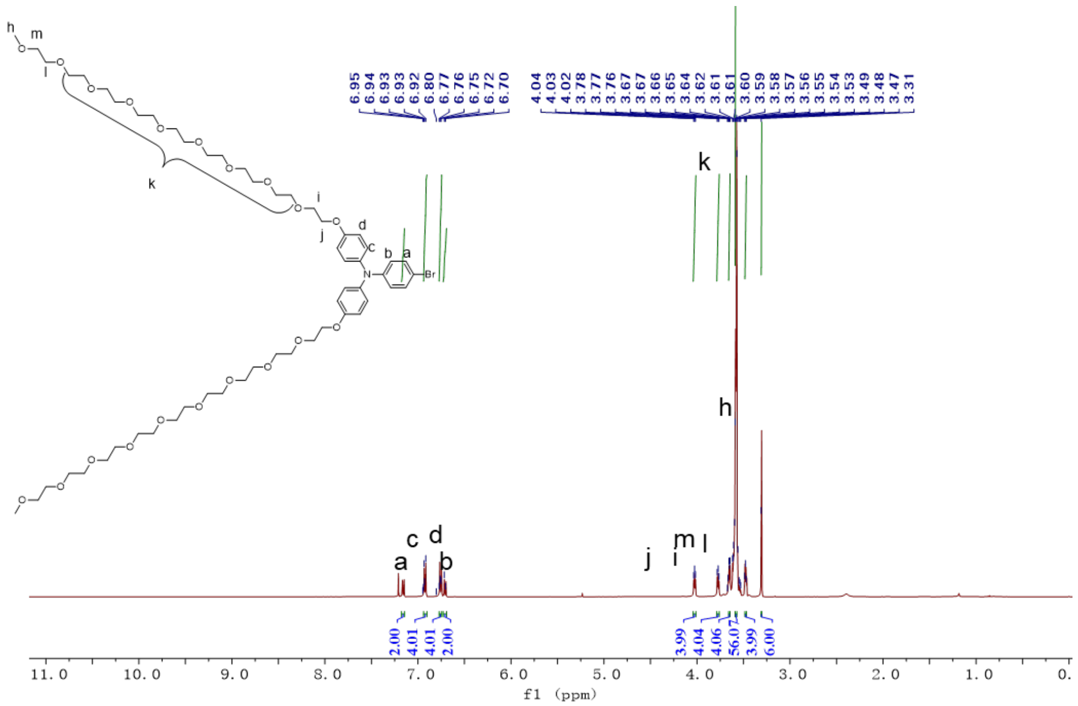

Figure S13. ^{1}H NMR spectrum of PEG_{420}-TPA-Br.

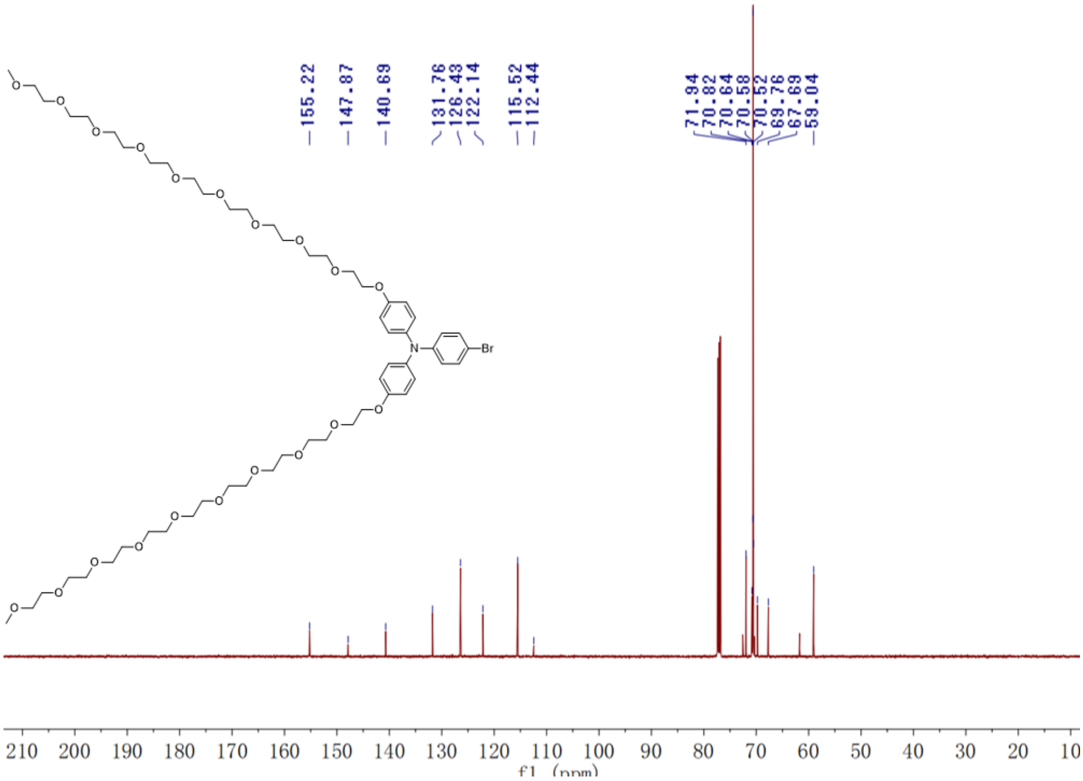

Figure S14. ^{13}C NMR spectrum of PEG_{420}-TPA-Br.

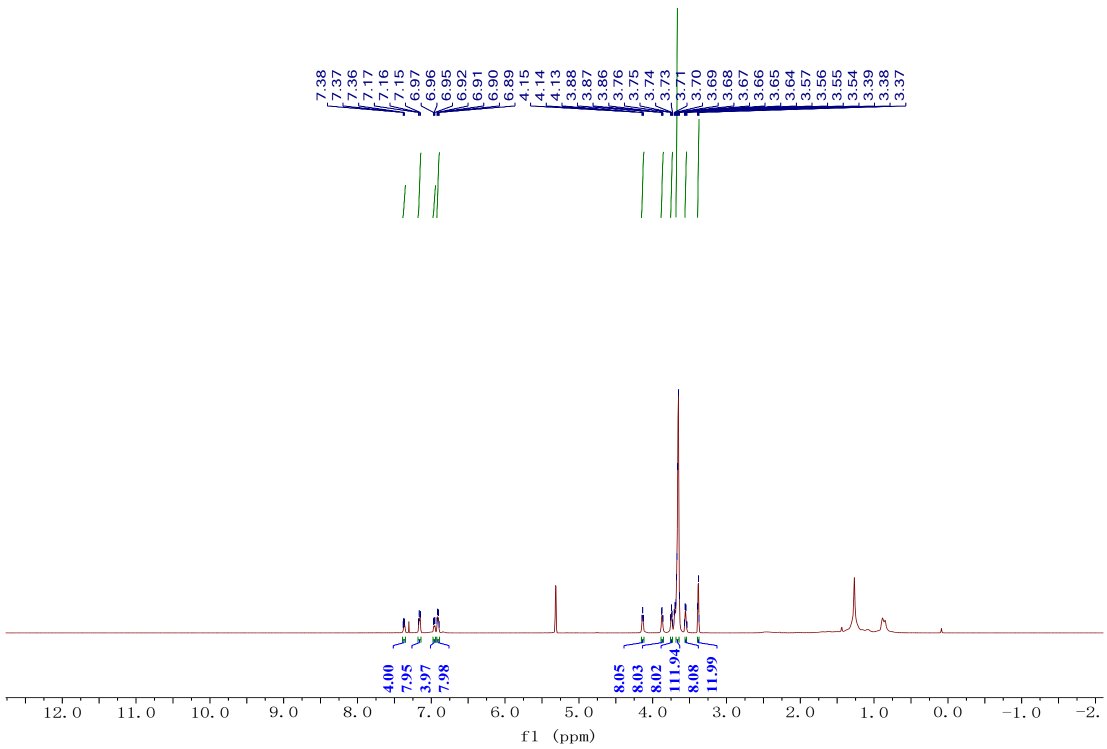

Figure S15. ^{1}H NMR spectrum of PEG_{420}-TPA-NO_2.

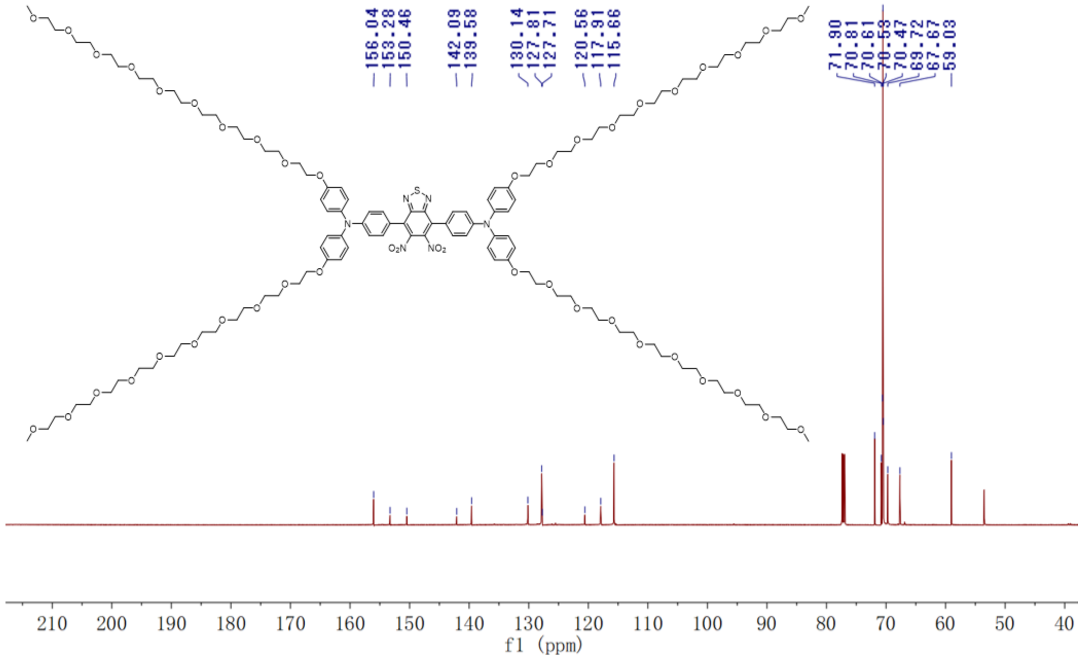

Figure S16. ^{13}C NMR spectrum of PEG_{420}-TPA-NO_2.

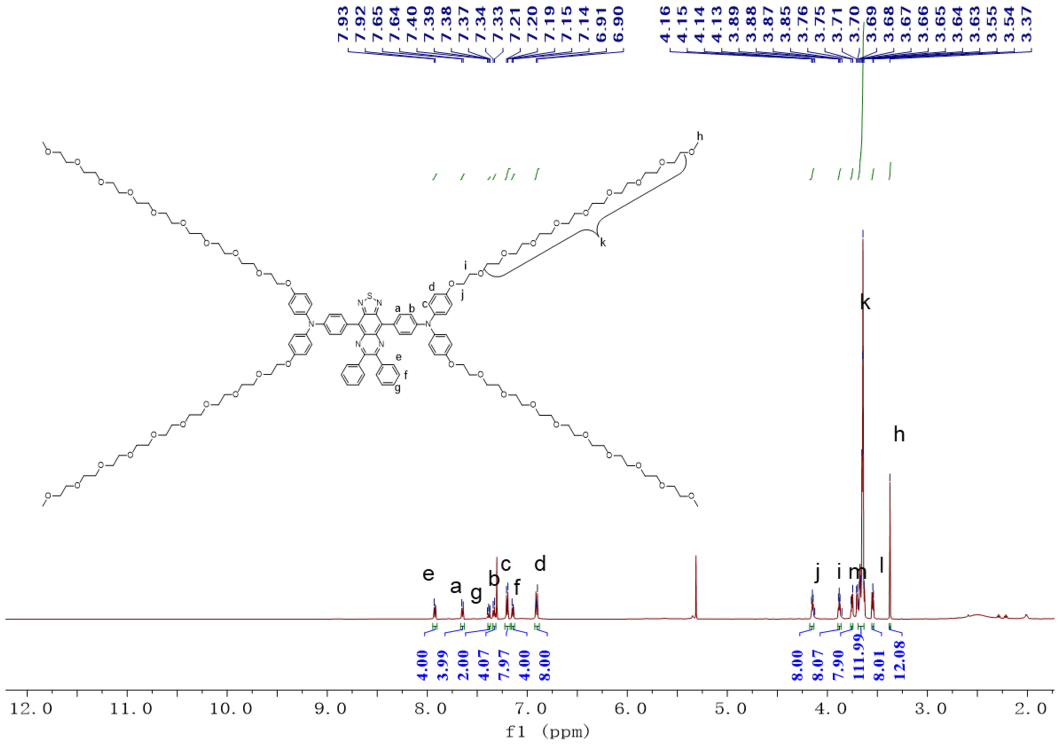

Figure S17. ^{1}H NMR spectrum of PEG$_{420}$-TQ.

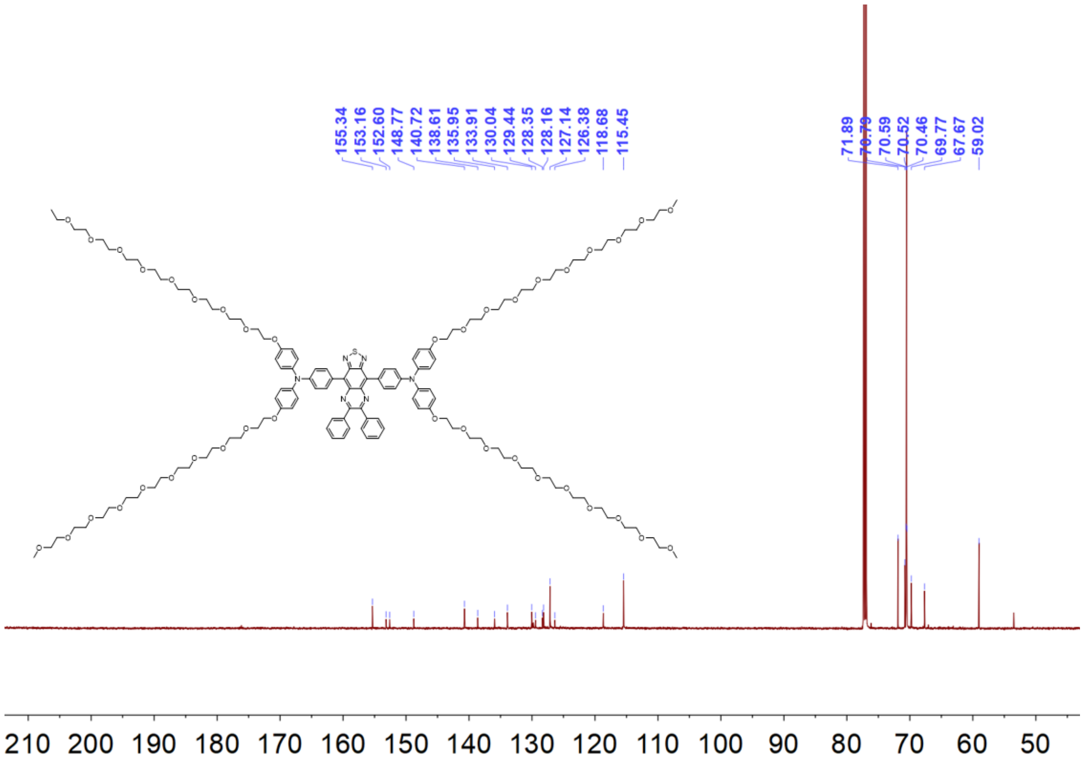

Figure S18. ^{13}C NMR spectrum of PEG$_{420}$-TQ.

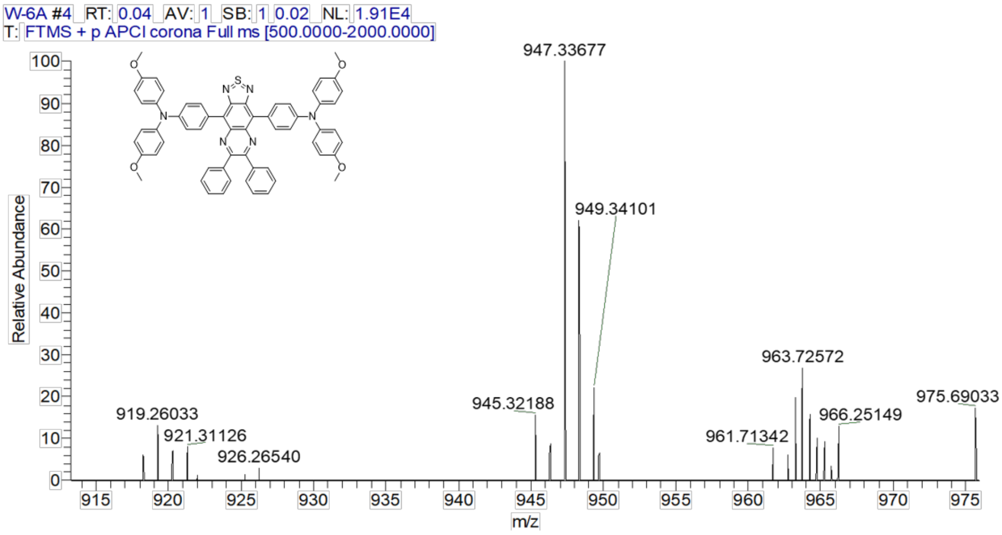

Figure S19. HRMS spectrum of OMe-TQ.

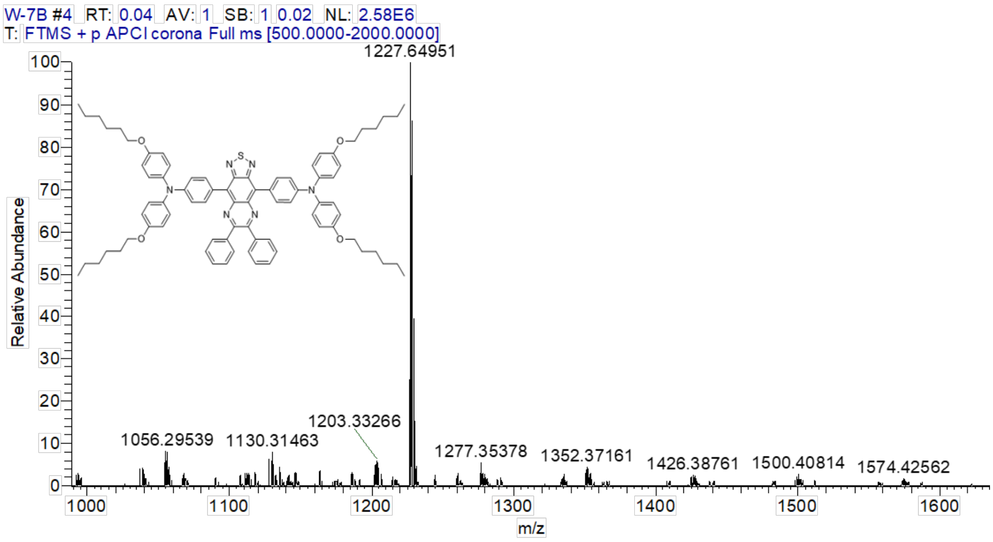

Figure S20. HRMS spectrum of OC6-TQ.

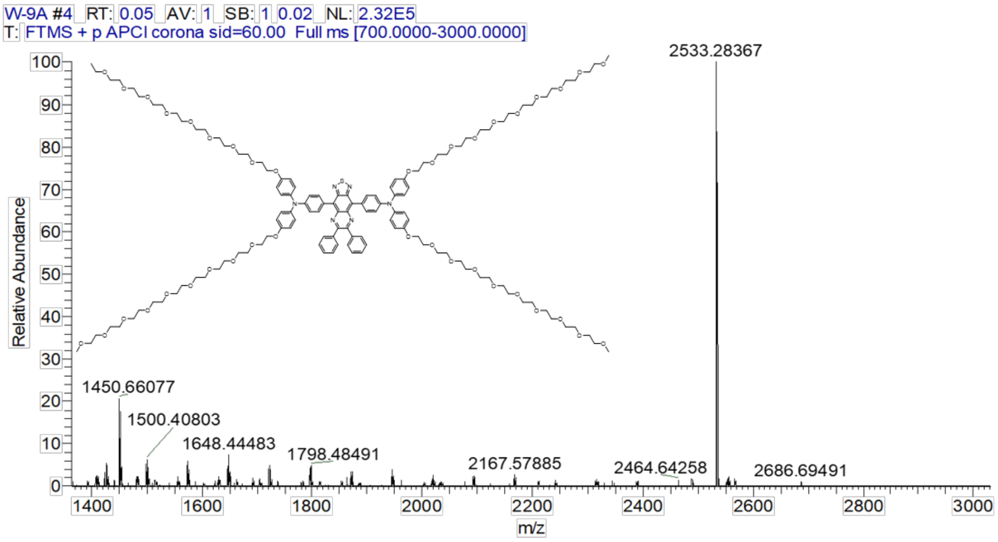

Figure S21. HRMS spectrum of PEG_{420}-TQ.

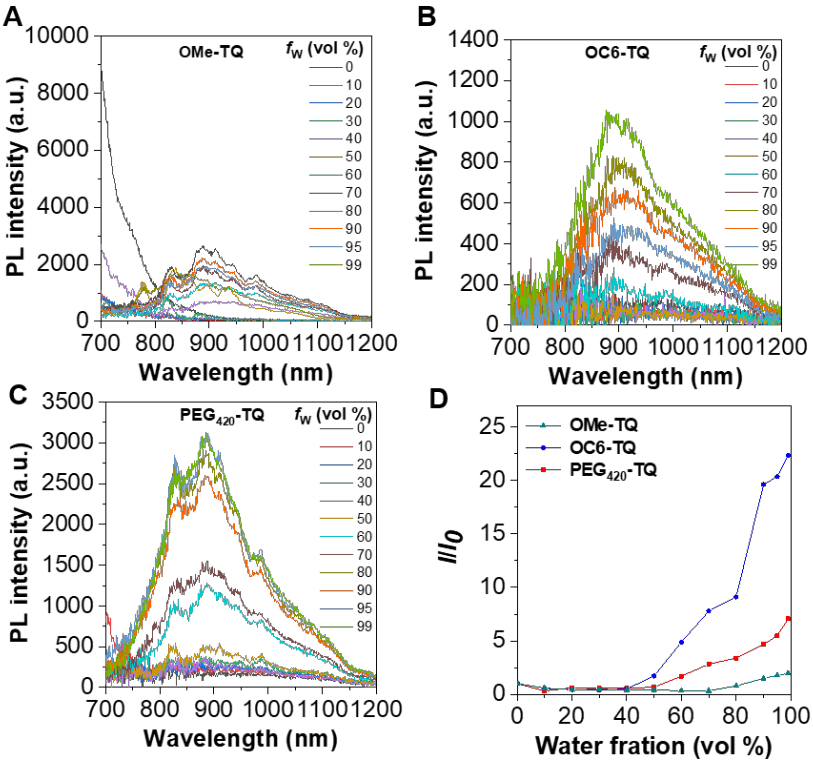

Figure S22. PL spectra of (A) OMe-TQ, (B) OC6-TQ and (C) PEG_{420}-TQ in THF/Water mixtures with different water fractions (f_W), 10 μM; (D) Plots of the relative emission intensity of the compounds versus water fraction. I_0 and I are the peak values of photoluminescence intensities of the compounds (10 μM) in THF and THF/water mixtures, respectively.

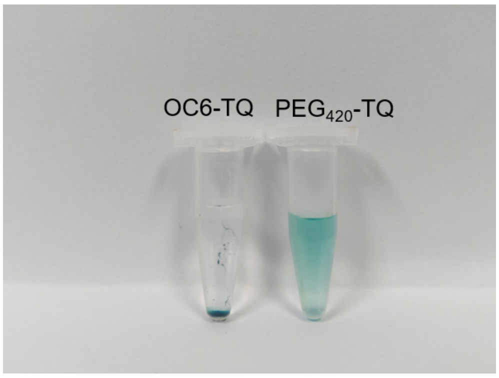

Figure S23. Water solubility study of OC6-TQ and PEG_{420}-TQ.

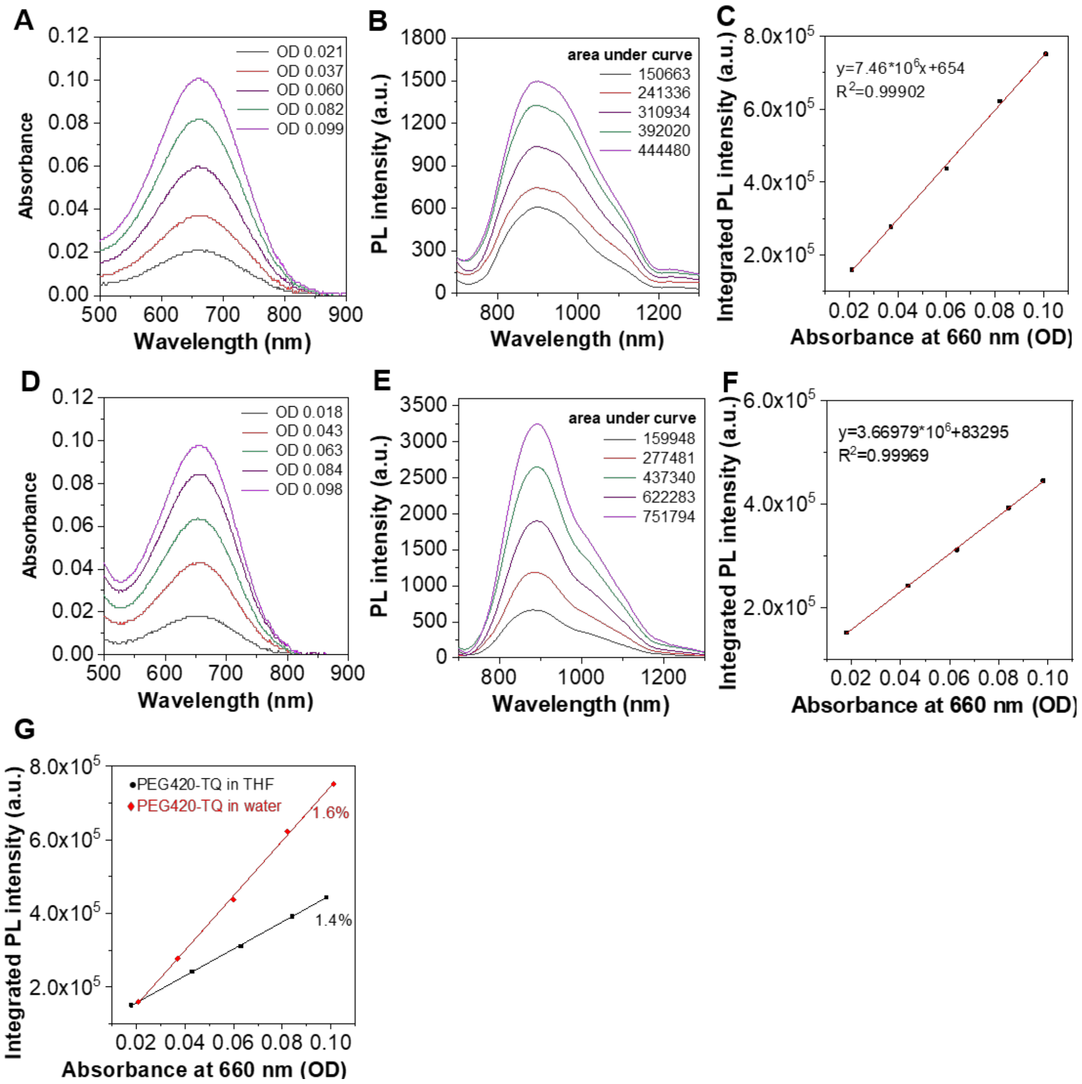

Figure S24. Fluorescence quantum yield calculation. UV-vis-NIR absorption spectra, PL spectra, and a plot of integrated fluorescence intensity versus the absorbance at 660 nm of PEG420-TQ (A, B, C) in water and (D, E, F) in THF. (G) The integrated fluorescence spectra (800-1200 nm) of corresponding samples versus different absorbance at 660 nm.

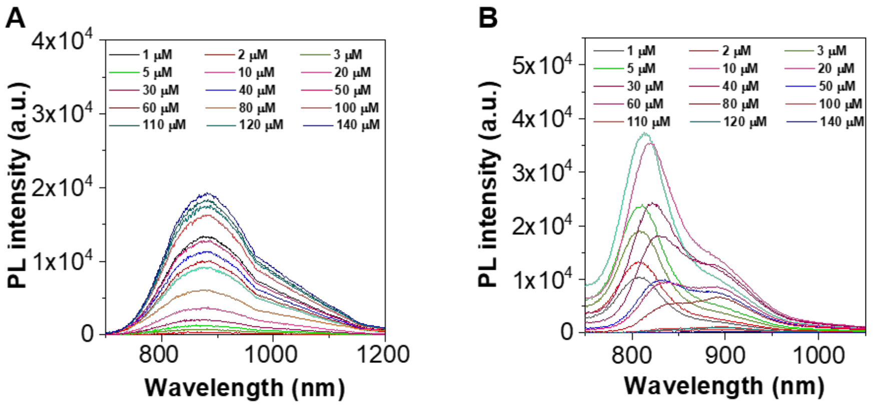

Figure S25. PL spectra of (A) PEG420-TQ and (B) ICG at different concentrations.

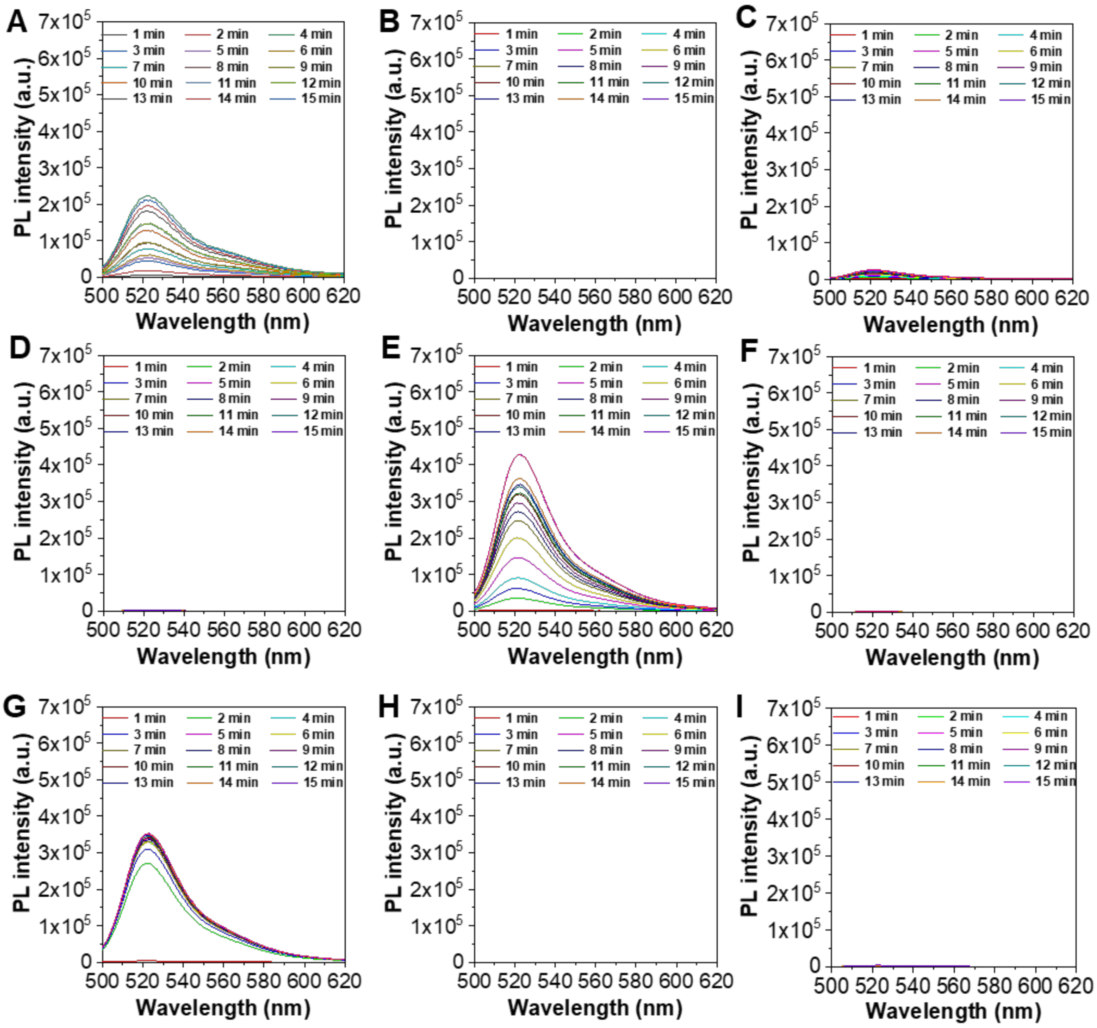

Figure S26. PL spectra of DCFH in the present of (A) DCFH + PEG420-TQ and (B) PEG420-TQ (1 μM), (C) DCFH + OC6-TQ and (D) OC6-TQ (1 μM), (E) DCFH + OMe-TQ and (F) OMe-TQ (1 μM), (G) DCFH + Ce6, (H) Ce6 (1 μM) and (I) DCFH when irradiated by 660 nm laser at a power density of 0.3 W cm^{-2}.

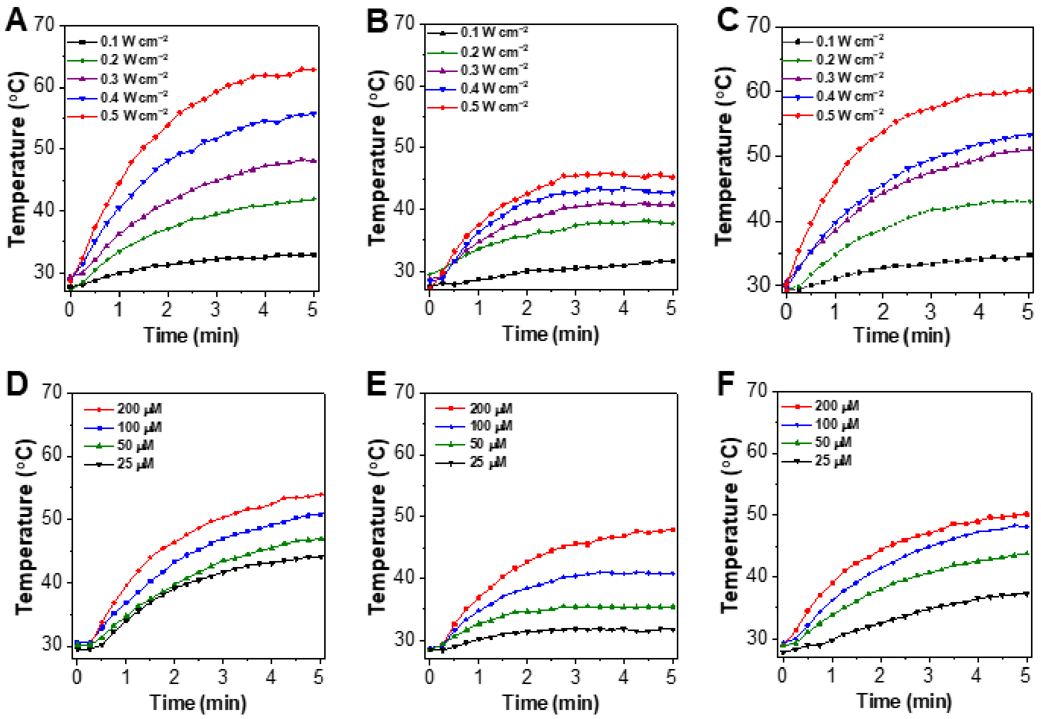

Figure S27. Thermal performance of (A) PEG420-TQ, (B) OC6-TQ, (C) OMe-TQ (100 μM) in aqueous solution under 660 nm laser irradiation with different exposure intensity. Photothermal performance of (D) PEG420-TQ, (E) OC6-TQ, (F) OMe-TQ as a function of concentration under 5 min illumination.

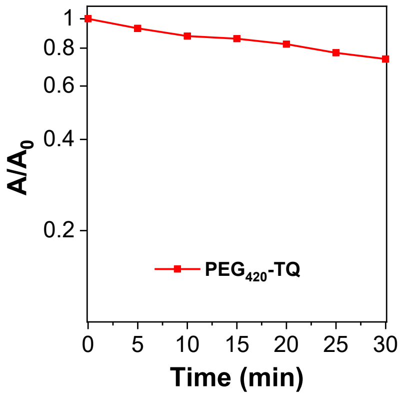

Figure S28. UV-vis absorption spectrum of PEG420-TQ irradiated with a 660 nm laser at 0.3 W cm^{-2} for different durations.

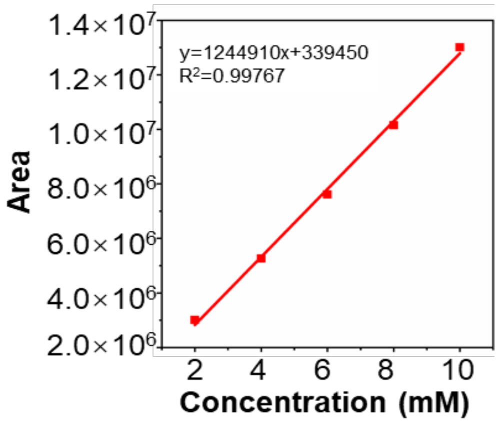

Figure S29. Standard curve of R837.

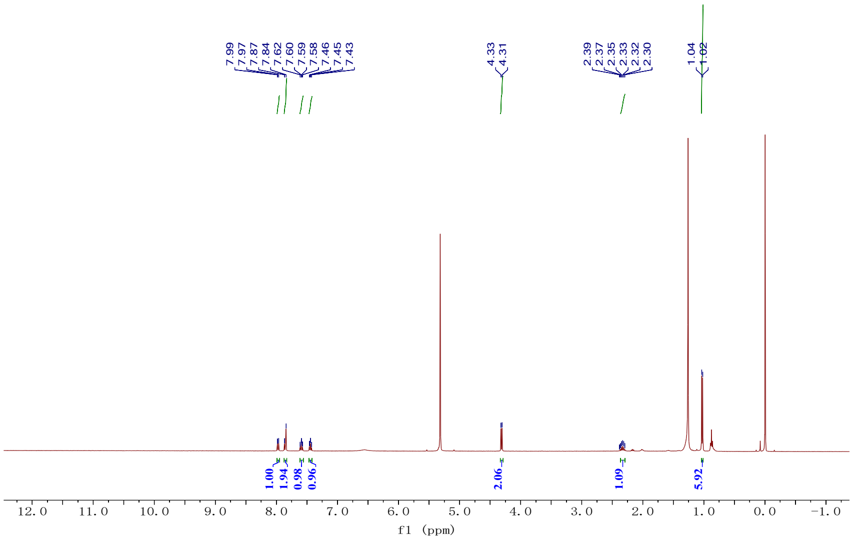

Figure S30. ^{1}H NMR spectrum of R837.

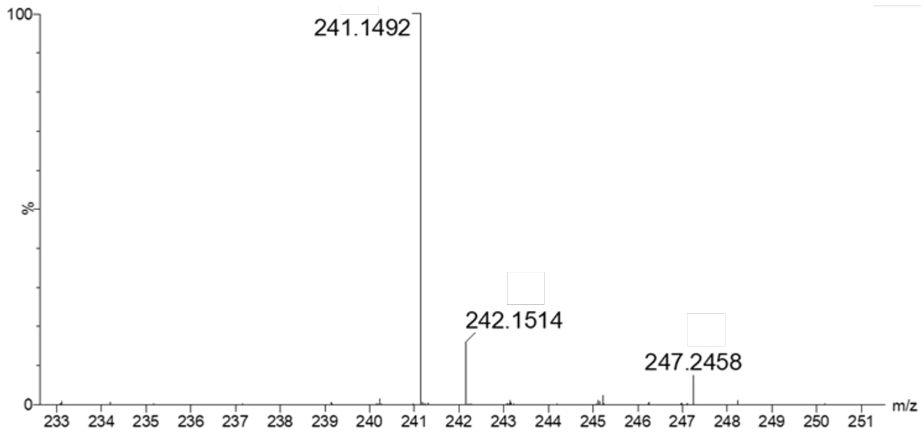

Figure S31. HRMS spectrum of R837.

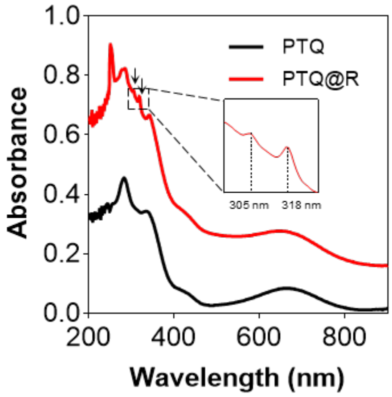

Figure S32. Absorption spectra of PTQ and PTQ@R. The peaks of R837 were pointed by arrows.

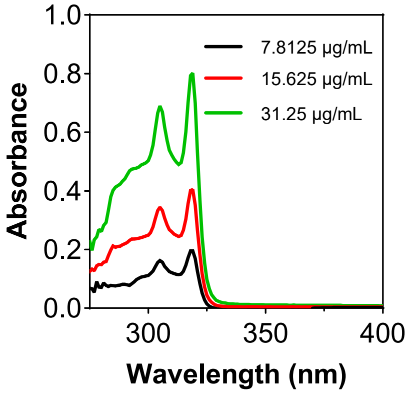

Figure S33. Absorption spectra of R837 with different concentrations.

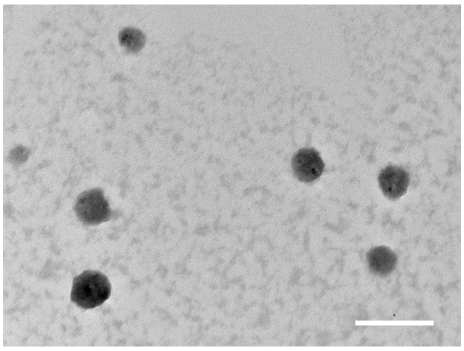

Figure S34. TEM image of PTQ@R. Scale bar: 200 nm.

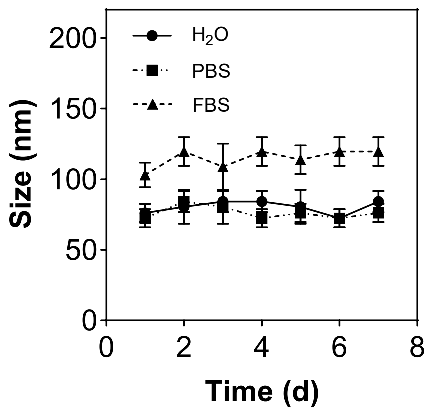

Figure S35. The particle size change of PTQ@R dispersed in H_2O, PBS and FBS determined by DLS.

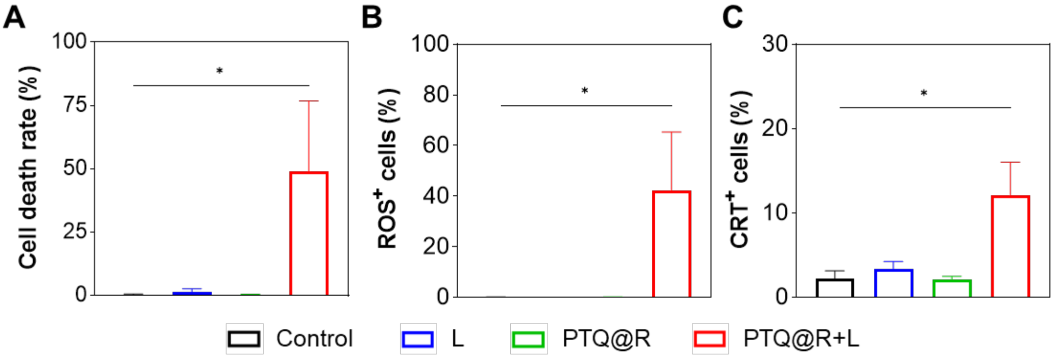

Figure S36. The quantitative results of (A) cell death rate, (B) ROS^+ cells and (C) CRT^+ cells after different treatments. **P* < 0.05.

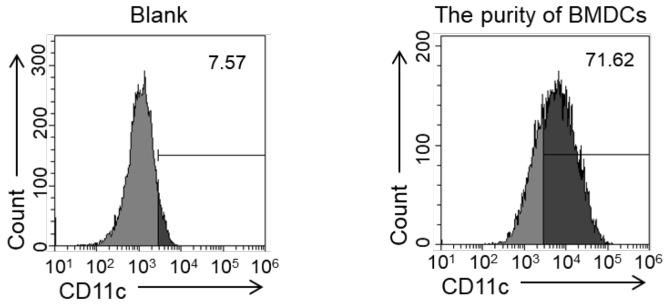

Figure S37. Flow cytometric analysis of the purity of extracted BMDCs.

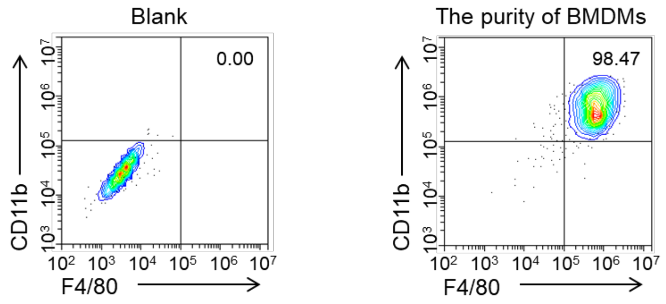

Figure S38. Flow cytometric analysis of the purity of extracted BMDMs.

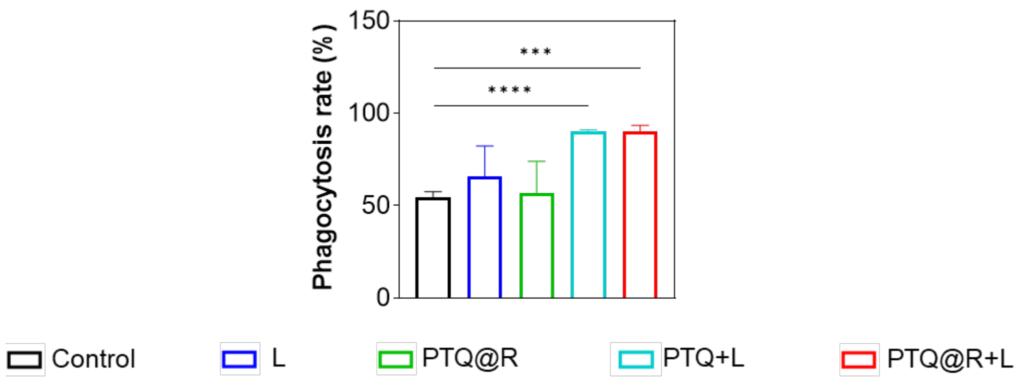

Figure S39. The quantitative results of BMDMs phagocytosis of 4T1 cells after different treatments. ***$P < 0.001$, ****$P < 0.0001$.

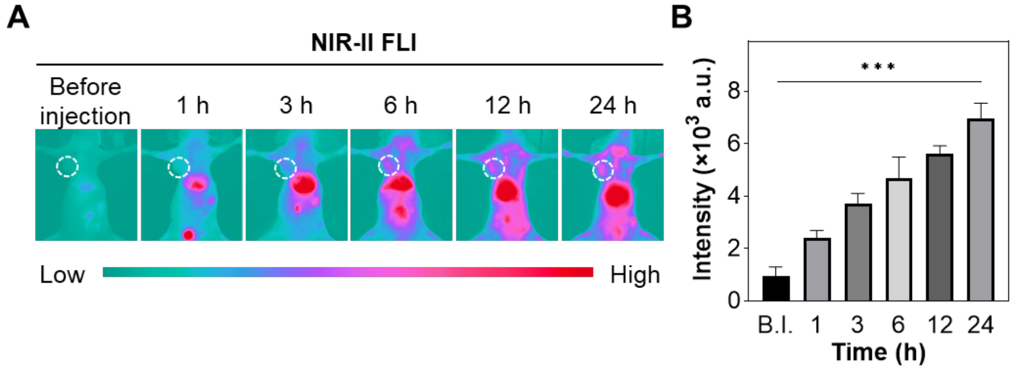

Figure S40. *In vivo* NIR-II FLI on 4T1 tumor-bearing mice. (A) NIR-II FLI (with an excitation wavelength of 660 nm) of 4T1 tumor-bearing mice at different monitoring times after administration of PTQ@R. The white dashed circles indicate tumor areas. (B) Fluorescence intensity of tumors *in vivo* at different monitoring times. B.I.: before injection. ***$P < 0.001$.

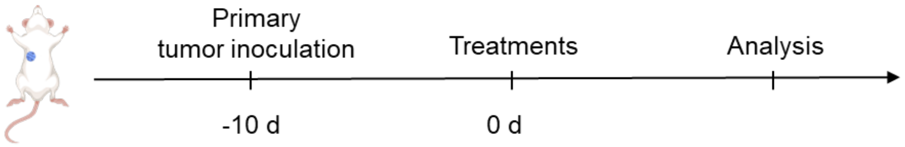

Figure S41. Schematic illustration of treatment of the primary tumor.

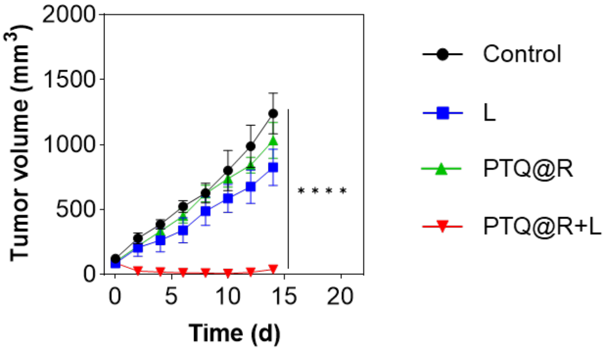

Figure S42. Average primary tumors growth curves of mice after various treatments. ****$P < 0.0001$.

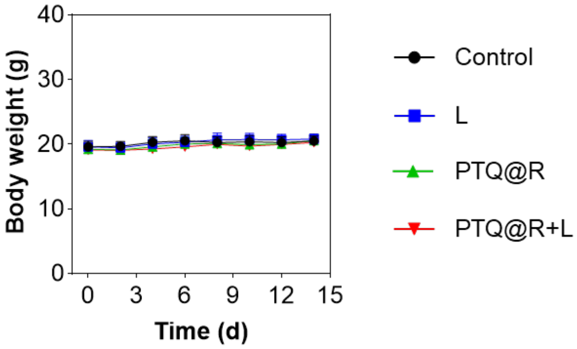

Figure S43. Body weight curves of 4T1 tumor-bearing mice after various treatments.

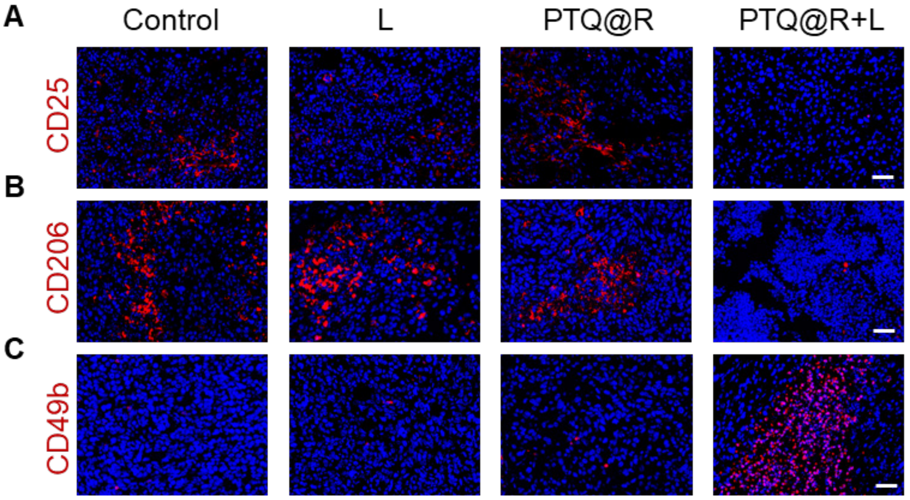

Figure S44. Representative immunofluorescence images of primary tumor stained with (A) CD25, (B) CD206 and (C) CD49b. Scale bar: 30 μm.

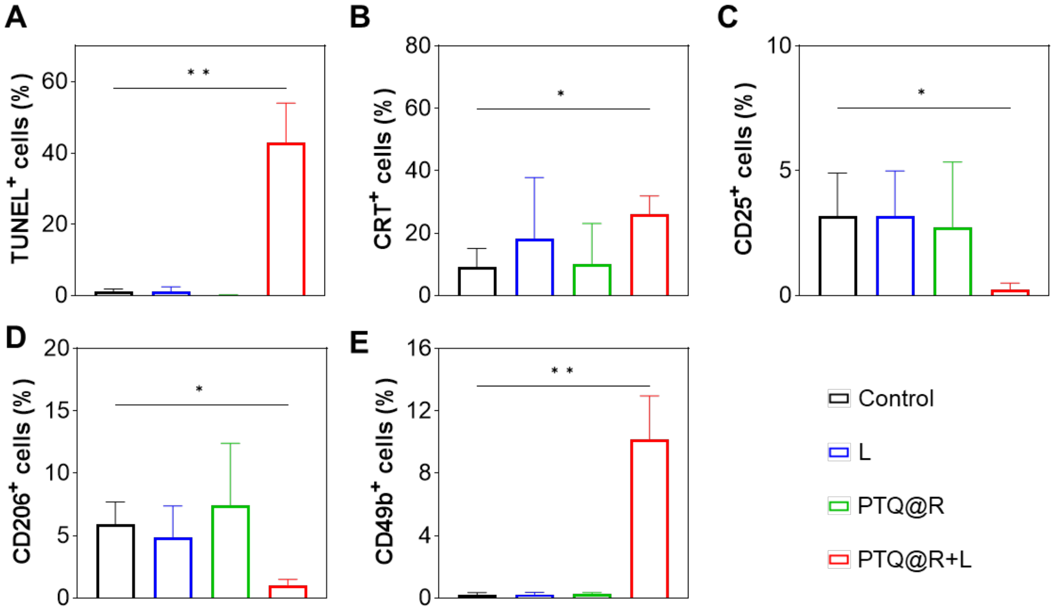

Figure S45. The quantitative results of (A) TUNEL$^+$, (B) CRT$^+$, (C) CD25$^+$, (D) CD206$^+$ and (E) CD49b$^+$ cells in primary tumors after different treatments. *$P < 0.05$, **$P < 0.01$.

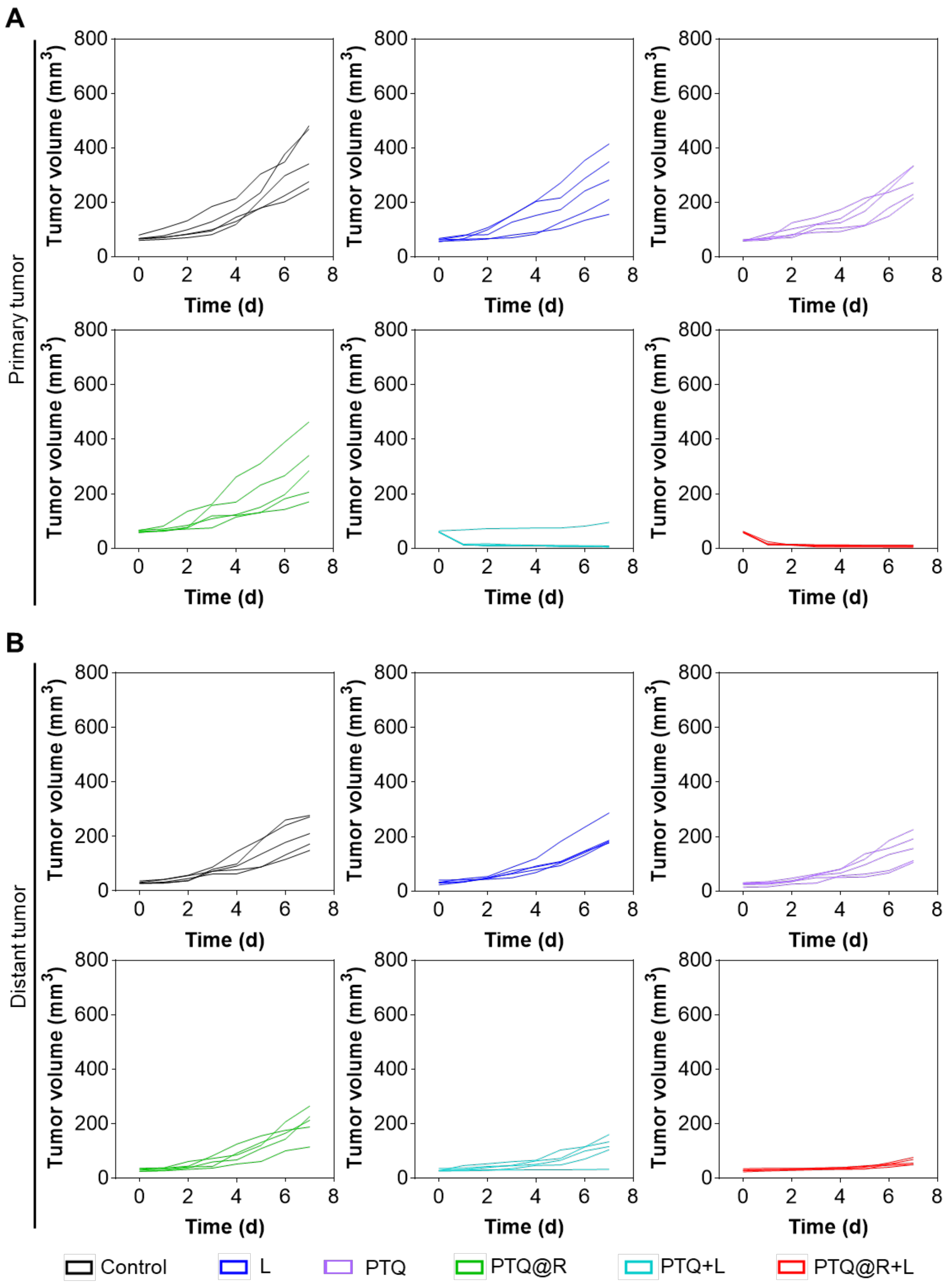

Figure S46. Individual tumor growth curves of the (A) primary or (B) distant tumors during different treatments.

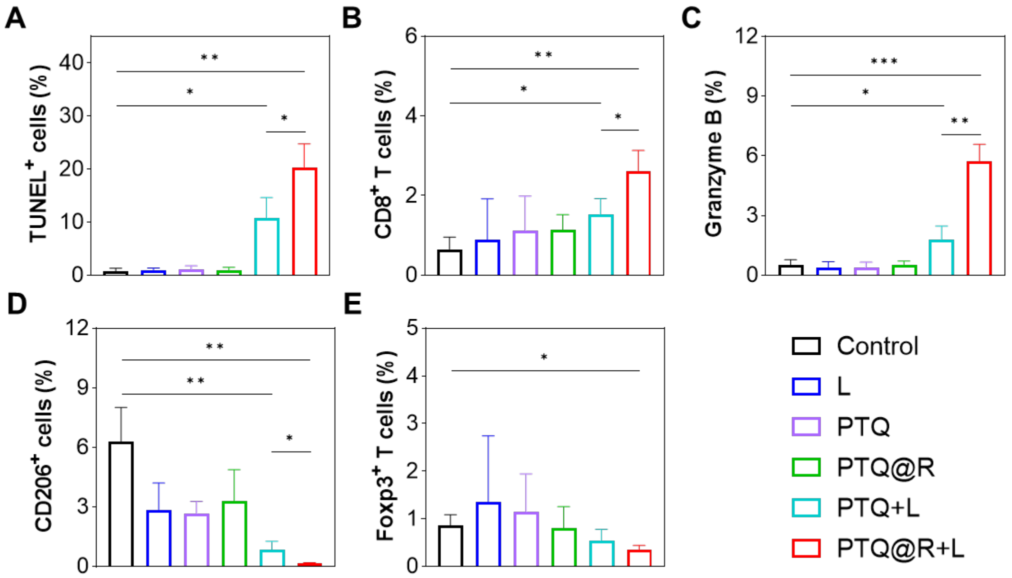

Figure S47. The quantitative results of (A) TUNEL$^+$ cells, (B) CD8$^+$ T cells, (C) Granzyme B, (D) CD206$^+$ cells and (E) Foxp3$^+$ T cells in distant tumors after different treatments. **P* < 0.05, ***P* < 0.01, ****P* < 0.001.

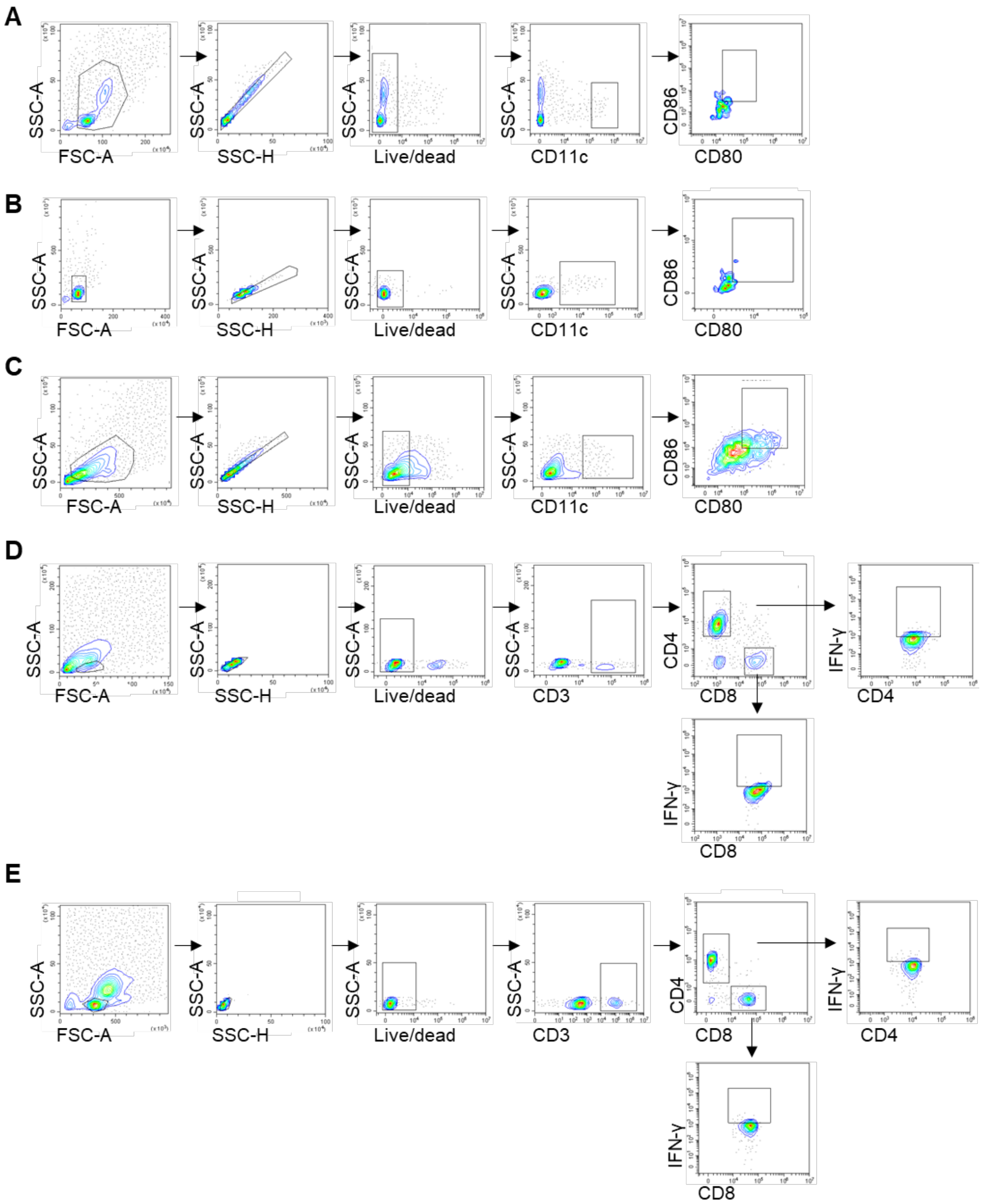

Figure S48. The gating strategies for cytometric analysis of DCs and T lymphocytes. Gating strategies for the analysis of DCs in (A) spleens, (B) lymph nodes and (C) primary tumors by flow cytometry. Gating strategies for the analysis of T lymphocytes in (D) distant tumors and (E) spleens by flow cytometry.

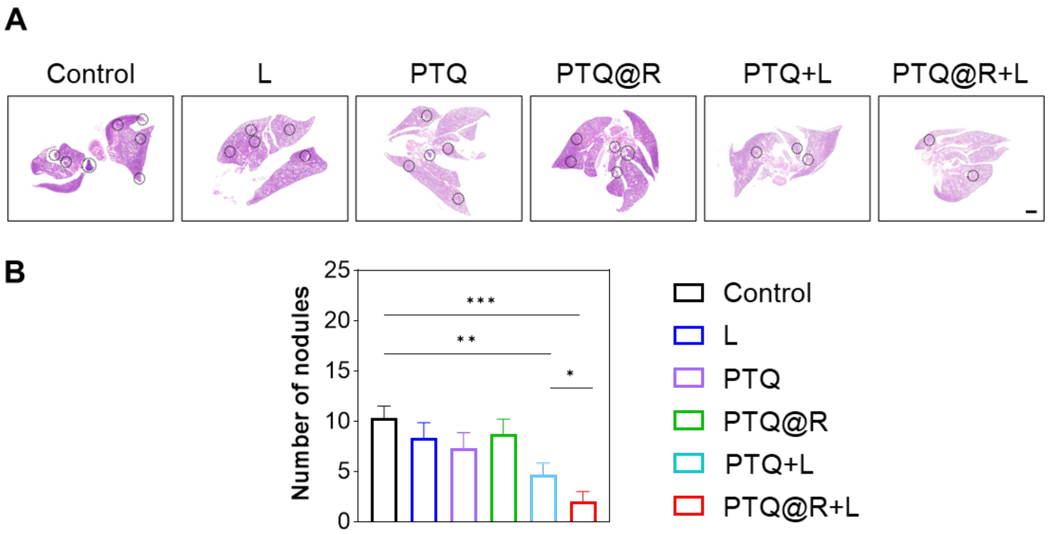

Figure S49. (A) Representative H&E staining images of lungs harvested from mice. Scale bar: 1 mm. (B) Number of pulmonary metastatic nodules from mice after various treatments. *$P < 0.05$, **$P < 0.01$, ****$P < 0.0001$.

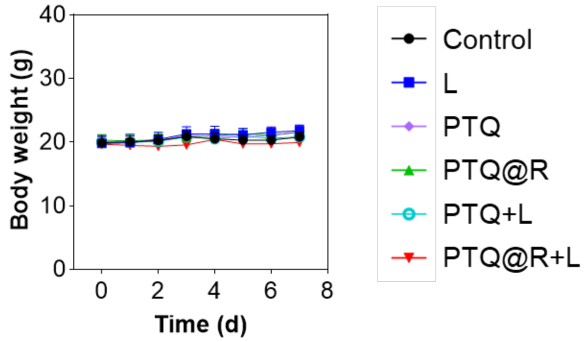

Figure S50. Body weight curves of tumor-bearing mice after various treatments.

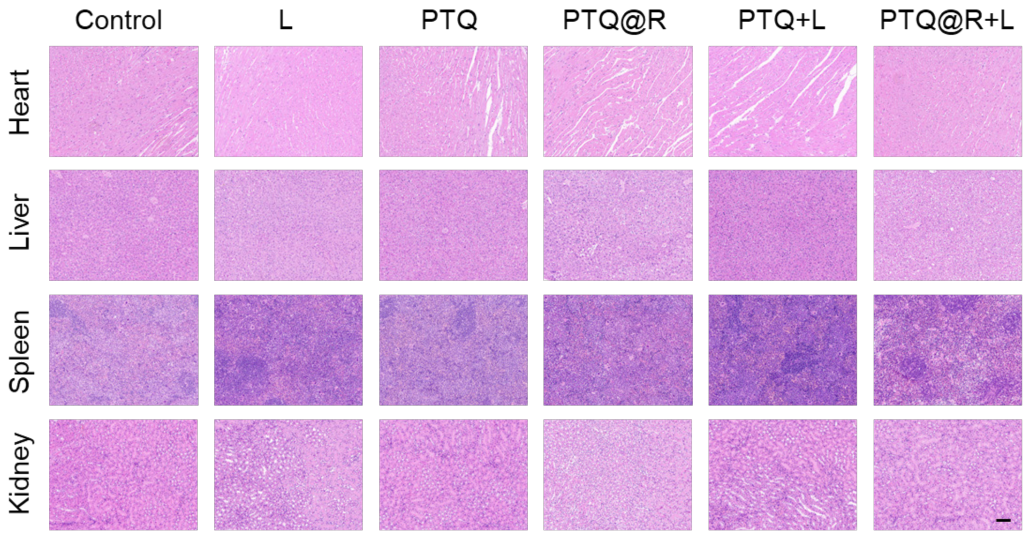

Figure S51. H&E images of hearts, livers, spleens and kidneys were harvested from mice at day 7 after various treatments. Scale bar: 100 μm.

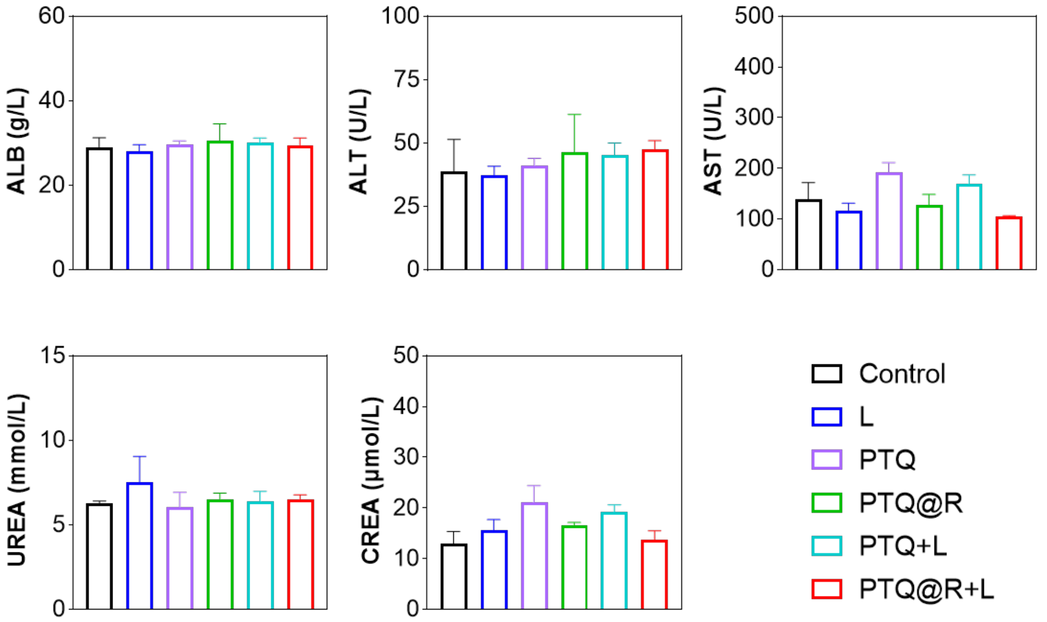

Figure S52. Blood biochemistry analysis after different treatments (ALB: albumin, ALT: alanine aminotransferase, AST: aspartate aminotransferase, UREA: urea, CREA: creatinine).

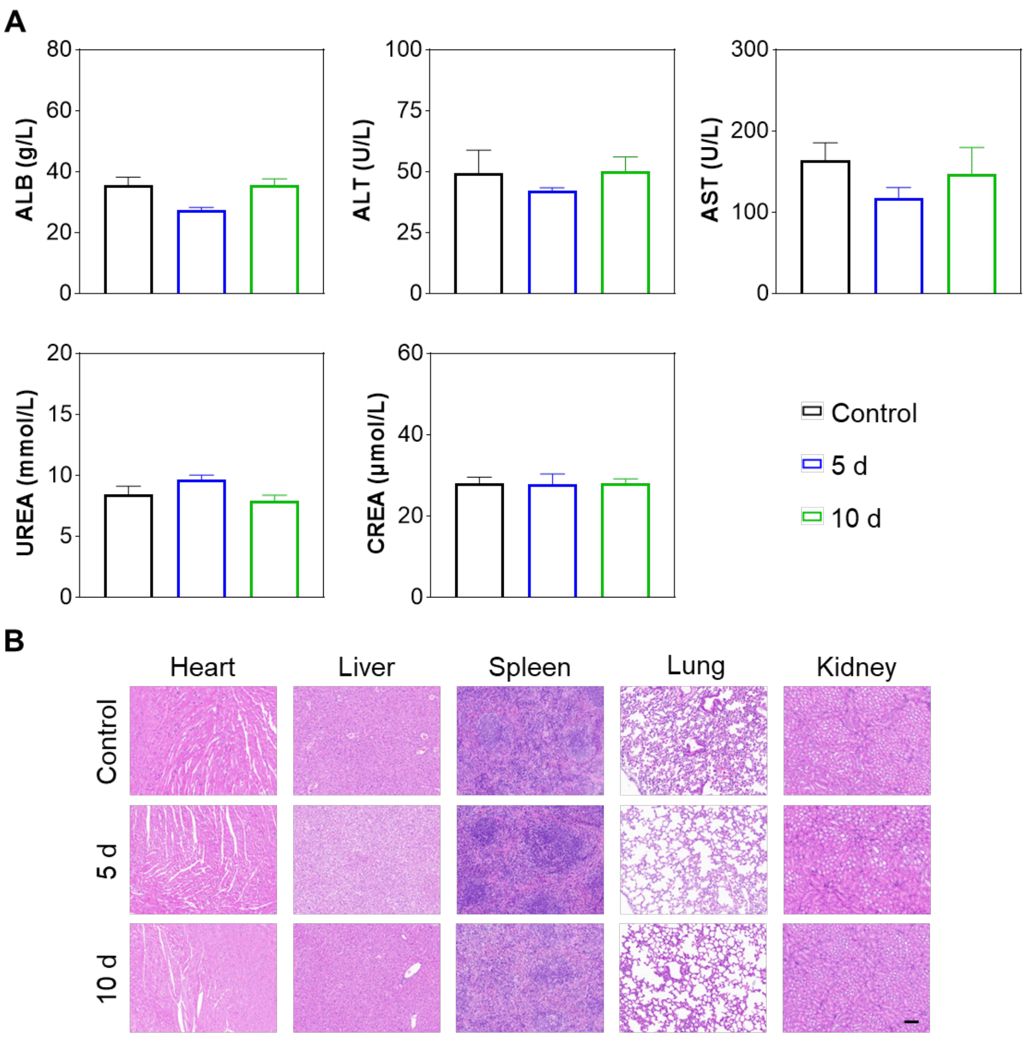

Figure S53. (A) Blood biochemistry analysis after treatment with PTQ@R (ALB: albumin, ALT: alanine aminotransferase, AST: aspartate aminotransferase, UREA: urea, CREA: creatinine). (B) Representative H&E staining images of major organs from PTQ@R treated mice at 5 and 10 days post-injection. Scale bar: 100 μm.

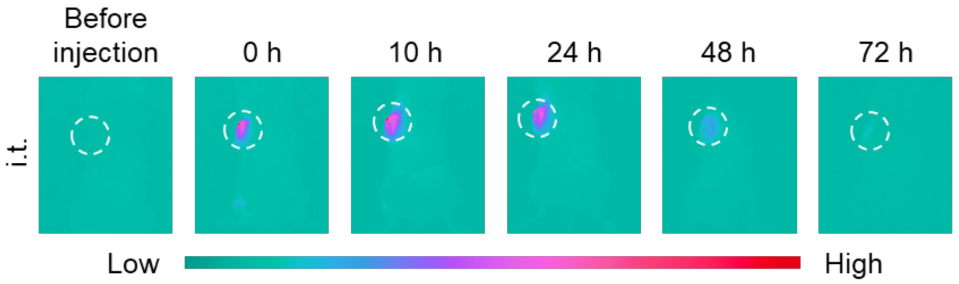

Figure S54. The clearance of PTQ@R post-injection was monitored by NIR-II FLI (i.t.: intratumor injection). The white dashed circles indicate tumor areas.

Table S1. The Clog *P* values of OMe-TQ, OC6-TQ and PEG_{420}-TQ [6].

Compound	OMe-TQ	OC6-TQ	PEG_{420}-TQ
Clog *P*	14.0	23.4	12.8

Table S2. Cartesian coordinates for DFT optimized structure (at B3LYP/6-31g (d) level) of OMe-TQ.

C	1.52268100	-0.75690300	-0.11799800
C	0.74859400	0.41366700	0.02699400
C	-0.70632300	0.40314700	0.02007300
C	-1.46204200	-0.78297000	-0.09152200
C	-0.69158000	-1.97528000	-0.22039500
C	0.77089400	-1.96077000	-0.24955100
N	-1.20748400	-3.21241700	-0.35488700
S	0.05721000	-4.23823000	-0.48175100
N	1.30592100	-3.18990000	-0.38293000
C	9.48042200	-0.60525600	3.43075600
C	9.84713100	0.14904700	2.30622300
C	9.11113700	0.06556700	1.13293700
C	7.99865000	-0.78910800	1.04111400
C	7.64787300	-1.54886000	2.16239100
C	8.36994800	-1.45314300	3.35409800
N	7.25818300	-0.88501400	-0.17297900
C	7.96737300	-1.05341200	-1.39742000
C	5.84689500	-0.84994800	-0.15868200
C	7.63987500	-0.28584800	-2.52960800
C	8.33519100	-0.44448400	-3.71957800
C	9.39527200	-1.35943200	-3.80770600
C	9.73908000	-2.11880100	-2.68420800
C	9.01962700	-1.96960300	-1.49624200
C	5.09326000	-1.59193100	-1.08566800
C	3.70501100	-1.55317800	-1.06470000
C	3.00022200	-0.77229000	-0.12915800
C	3.76436900	-0.03309100	0.79489000

C	5.15203100	-0.06993600	0.78465200
O	10.02185400	-1.42943100	-5.02081000
O	10.26404800	-0.44145300	4.53905200
O	-10.08798800	0.33674500	-4.68652000
O	-9.94168100	-2.63448100	4.53309700
C	-5.01769200	-1.86155900	0.66740000
C	-3.63047600	-1.79774400	0.65784100
C	-2.93912900	-0.82317700	-0.08680600
C	-3.71582800	0.08512700	-0.83194100
C	-5.10294800	0.02588400	-0.83222400
C	-7.58501200	-0.93708700	2.35281200
C	-8.27596900	-1.36076200	3.47901500
C	-9.31792100	-2.29330700	3.36527800
C	-9.64827500	-2.80022800	2.10371600
C	-8.93334400	-2.38547000	0.97784400
C	-5.78421800	-0.94914400	-0.07984700
C	-7.89914600	-1.44937500	1.08134600
N	-7.19435000	-1.01020400	-0.07689000
C	-9.80500000	0.53986400	-2.25882900
C	-9.05268800	0.19659000	-1.13324500
C	-7.93858600	-0.64282400	-1.23574900
C	-7.58250600	-1.13109700	-2.50556200
C	-8.31169600	-0.77660200	-3.63157100
C	-9.43315700	0.05878900	-3.51899400
N	1.39739400	1.60587100	0.20669300
C	0.73136300	2.72628700	0.34079600
C	-0.72487600	2.72909000	0.21130200
N	-1.37360200	1.59606300	0.10018600
C	-1.56190600	3.95901200	0.14044300
C	1.54977300	3.92453400	0.67645300
C	1.12825300	4.86940000	1.62631300
C	1.95359200	5.93701700	1.97646500

C	3.20591100	6.08443800	1.37724400
C	3.63675000	5.14645600	0.43594600
C	2.82080000	4.06987500	0.09586700
C	-2.83338400	3.95711900	0.73790100
C	-3.66577500	5.06952100	0.63712800
C	-3.25114800	6.19436300	-0.08000300
C	-1.99848400	6.19781500	-0.69636600
C	-1.15674300	5.09221300	-0.58367200
C	11.09594800	-2.34293600	-5.16541700
C	-10.99472100	-3.58148000	4.47541100
C	9.93543400	-1.17932100	5.70391700
C	-11.22338400	1.18316600	-4.63009500
H	10.70713200	0.80739400	2.38094600
H	9.39604500	0.66248600	0.27212700
H	6.79545700	-2.21875900	2.10651800
H	8.06475800	-2.05389500	4.20334200
H	6.83048100	0.43492200	-2.46845600
H	8.08428300	0.14496300	-4.59604100
H	10.55099800	-2.83624700	-2.72080300
H	9.28504300	-2.57046700	-0.63197500
H	5.60153100	-2.20634400	-1.82118200
H	3.15586700	-2.14516900	-1.78780300
H	3.26127600	0.58502000	1.52785400
H	5.70841900	0.51229500	1.51159800
H	-5.51527500	-2.62776100	1.25234200
H	-3.07113300	-2.52277200	1.23780700
H	-3.22351700	0.85341000	-1.41481700
H	-5.66883500	0.74255300	-1.41796800
H	-6.78899600	-0.20548800	2.45043900
H	-8.03516400	-0.96906400	4.46247400
H	-10.44547100	-3.52496800	1.98312200
H	-9.18762700	-2.79163300	0.00373300

H	-10.66433400	1.18990200	-2.13923800
H	-9.34029100	0.58226000	-0.16001700
H	-6.72438000	-1.78886300	-2.60217600
H	-8.03907400	-1.14992700	-4.61384100
H	0.15992900	4.76206900	2.10384300
H	1.61712900	6.65316700	2.72130300
H	3.84430500	6.92195700	1.64580100
H	4.61274800	5.25069400	-0.03055400
H	3.15809200	3.32367400	-0.61595400
H	-3.15787300	3.07020800	1.27210800
H	-4.64188100	5.05652200	1.11478300
H	-3.90227200	7.06062300	-0.16225700
H	-1.67446200	7.06231400	-1.26951100
H	-0.18823100	5.10450000	-1.07259000
H	11.44427400	-2.23654100	-6.19455100
H	11.92085700	-2.11040200	-4.47831700
H	10.77169900	-3.37903600	-4.99848000
H	-11.34303400	-3.70632900	5.50247600
H	-11.82628200	-3.22540700	3.85215900
H	-10.64778100	-4.55003800	4.09072500
H	10.67409400	-0.89703300	6.45656500
H	8.93078300	-0.92993500	6.07129400
H	9.99413200	-2.26196400	5.52783400
H	-11.58444000	1.27026400	-5.65665600
H	-12.01705400	0.75535100	-4.00255000
H	-10.96600600	2.18153700	-4.25121100

Table S3. Cartesian coordinates for DFT optimized structure (at B3LYP/6-31g (d) level) of OC6-TQ.

C	1.65420500	-0.10997900	0.29595100
C	0.85299600	-0.18722000	-0.86288900
C	-0.60132700	-0.17839400	-0.81852300
C	-1.32939000	-0.05149000	0.38336400

C	-0.53123100	0.04738800	1.56031200
C	0.93042200	0.00262200	1.51886700
N	-1.01809800	0.16095600	2.81120800
S	0.27013900	0.22500600	3.81340900
N	1.49395200	0.10639400	2.73805400
C	9.63859500	3.23380500	-0.75601800
C	9.97196000	1.97417100	-1.27667200
C	9.22746400	0.85288600	-0.93921300
C	8.13896600	0.95193800	-0.05526800
C	7.82096800	2.20860000	0.47126100
C	8.55142600	3.34552200	0.11859700
N	7.39048300	-0.20708700	0.30360700
C	8.09453600	-1.38093700	0.70101100
C	5.97950400	-0.18228800	0.29832500
C	7.74409800	-2.64034600	0.18247200
C	8.43493600	-3.78154200	0.56360600
C	9.51331200	-3.69682700	1.45775000
C	9.87945200	-2.44676500	1.96941800
C	9.16479300	-1.30547900	1.59835900
C	5.23693200	-0.93722500	1.22412000
C	3.84846100	-0.90742100	1.21330500
C	3.13143100	-0.13432500	0.28046800
C	3.88435500	0.61823000	-0.64207400
C	5.27234100	0.59893800	-0.63510600
O	10.13253700	-4.87705400	1.75762300
O	10.42748400	4.27350400	-1.15993500
O	-9.98071800	-4.72833800	0.36478200
O	-9.76272200	4.88452800	1.53919200
C	-4.85727800	0.92447800	1.37593800
C	-3.47202600	0.89446500	1.28297400
C	-2.80497200	-0.02867400	0.45520700
C	-3.60444800	-0.93041300	-0.27383200

C	-4.98962600	-0.91136200	-0.18310200
C	-7.45088800	2.41438600	0.21850800
C	-8.13154100	3.60281600	0.44163000
C	-9.14745100	3.67185300	1.40717900
C	-9.46127200	2.52863900	2.15123300
C	-8.75641500	1.34224800	1.93629100
C	-5.64674200	0.01898000	0.64400300
C	-7.74856300	1.26347300	0.96957400
N	-7.05446700	0.04035800	0.73724500
C	-9.70250600	-2.38537800	-0.30480800
C	-8.94182900	-1.21870600	-0.20083100
C	-7.80767600	-1.16402200	0.61594000
C	-7.44056400	-2.31865400	1.32959000
C	-8.17847300	-3.48808600	1.21406300
C	-9.31997100	-3.53307200	0.39916700
N	1.47390900	-0.24867900	-2.08156100
C	0.78197100	-0.32801800	-3.19149900
C	-0.67442600	-0.44102700	-3.13647200
N	-1.29637600	-0.32437500	-1.98905500
C	-1.54135600	-0.74042200	-4.31007000
C	1.57345400	-0.23987900	-4.45018500
C	1.13537000	0.51324200	-5.55179700
C	1.93685700	0.64047300	-6.68540400
C	3.18134400	0.01026500	-6.74179600
C	3.62869000	-0.73578100	-5.64874800
C	2.83684100	-0.85151200	-4.50854200
C	-2.80945900	-0.14180700	-4.39488700
C	-3.66919900	-0.44810600	-5.44727300
C	-3.28556400	-1.37322900	-6.42114500
C	-2.03633100	-1.99039100	-6.33388300
C	-1.16745400	-1.67393600	-5.29055400
C	11.23127100	-4.86259600	2.66565200

C	11.72597300	-6.29440400	2.82089900
C	12.91664400	-6.40566700	3.78160400
C	13.42421600	-7.84401100	3.94691500
C	14.61536600	-7.96579800	4.90576200
C	15.11629600	-9.40482500	5.06548800
C	-10.79741100	5.02527300	2.50948400
C	10.14303700	5.58056300	-0.66709700
C	-11.14273100	-4.84556400	-0.45227700
C	-11.30185100	6.46078800	2.44822400
C	-12.42667200	6.73306500	3.45505000
C	-12.94436000	8.17638900	3.40396800
C	-14.06991800	8.45799300	4.40741500
C	-14.58160200	9.90096700	4.35050900
C	-11.67065500	-6.26681900	-0.30990600
C	-12.93001700	-6.51470800	-1.14989900
C	-13.47098800	-7.94412800	-1.01622000
C	-14.73058600	-8.20209700	-1.85285100
C	-15.26426900	-9.63151000	-1.71434400
C	11.15847900	6.53897200	-1.27473800
C	10.95175800	7.98684800	-0.81213800
C	11.96856600	8.96337900	-1.41732400
C	11.77026700	10.41397400	-0.95956200
C	12.78905800	11.38305800	-1.56797500
H	10.81378300	1.90132900	-1.95837000
H	9.48728500	-0.11487300	-1.35693600
H	6.98727700	2.30188200	1.16035700
H	8.27187800	4.30288100	0.54316900
H	6.92036000	-2.71659400	-0.52045200
H	8.16600800	-4.75561300	0.16670000
H	10.70568000	-2.34762300	2.66404300
H	9.44822600	-0.34103000	2.00829000
H	5.75444200	-1.54470600	1.95889800

H	3.30873100	-1.49443200	1.94747900
H	3.37184300	1.22238000	-1.38012900
H	5.81944000	1.19151900	-1.36068800
H	-5.33543600	1.64951600	2.02590000
H	-2.89478700	1.60033700	1.86901800
H	-3.13153600	-1.65367200	-0.92625500
H	-5.57322900	-1.62156600	-0.75922500
H	-6.67571400	2.36952000	-0.54016600
H	-7.90293600	4.49388600	-0.13493200
H	-10.23804900	2.54978200	2.90699000
H	-8.99812500	0.46287400	2.52524700
H	-10.57733800	-2.38831200	-0.94486000
H	-9.23868100	-0.33565600	-0.75834500
H	-6.56688500	-2.29206300	1.97336200
H	-7.89689100	-4.38210500	1.76184600
H	0.17300000	1.01314300	-5.51751000
H	1.58792800	1.23585100	-7.52484400
H	3.80101400	0.10416600	-7.62967100
H	4.59891800	-1.22425800	-5.68221100
H	3.18755600	-1.40852800	-3.64599100
H	-3.10970000	0.55690700	-3.62098000
H	-4.64244500	0.03213400	-5.50450400
H	-3.95799800	-1.61544300	-7.23987800
H	-1.73620000	-2.72303200	-7.07821800
H	-0.20193400	-2.16515500	-5.23003000
H	12.03118600	-4.21431700	2.27707800
H	10.91112000	-4.45401300	3.63596400
H	10.89403600	-6.91545700	3.17723100
H	12.00280700	-6.67838500	1.83056700
H	13.73986800	-5.77079900	3.42180300
H	12.63258100	-6.00701200	4.76677000
H	12.60193700	-8.47987100	4.30685900

H	13.70833100	-8.24399100	2.96236600
H	15.43662200	-7.32992700	4.54548500
H	14.33067700	-7.56577600	5.88937700
H	15.96614900	-9.45847200	5.75537100
H	14.32712800	-10.05811400	5.45766500
H	15.44117300	-9.82062000	4.10373900
H	-11.61213800	4.31710600	2.29525300
H	-10.40669200	4.79249700	3.51151300
H	9.11865900	5.87170300	-0.94422500
H	10.20803500	5.59012100	0.43140300
H	-11.90143300	-4.11474100	-0.13369500
H	-10.88820900	-4.62813300	-1.50057100
H	-10.45727500	7.13687700	2.63360500
H	-11.65048000	6.66616900	1.42786000
H	-13.26234900	6.04243300	3.26821500
H	-12.07072700	6.51246400	4.47229800
H	-12.10975100	8.86810000	3.59094800
H	-13.30045400	8.39849000	2.38714200
H	-14.90346700	7.76612700	4.22001700
H	-13.71328900	8.23563800	5.42325100
H	-15.38375700	10.07007600	5.07783200
H	-13.77821600	10.61538900	4.56851900
H	-14.97707800	10.14249400	3.35619800
H	-10.87712700	-6.96586500	-0.60387700
H	-11.88041600	-6.45781400	0.75044700
H	-13.71463000	-5.80207500	-0.85531000
H	-12.71241400	-6.30798200	-2.20825400
H	-12.68726000	-8.65767100	-1.31054200
H	-13.68893100	-8.15229900	0.04170400
H	-15.51339300	-7.48867400	-1.55813400
H	-14.51203900	-7.99353000	-2.90988300
H	-16.16273700	-9.78397400	-2.32309400

H	-14.51600700	-10.36686400	-2.03483500
H	-15.52489200	-9.85831400	-0.67318200
H	12.16657700	6.19728600	-1.00666100
H	11.08662800	6.47801400	-2.36832800
H	9.93503400	8.31488400	-1.07441000
H	11.01366700	8.03443000	0.28517400
H	12.98586000	8.63615600	-1.15632100
H	11.90726900	8.91734400	-2.51466500
H	10.75336800	10.74010500	-1.22049900
H	11.83152700	10.45906700	0.13715300
H	12.62115100	12.40933400	-1.22211700
H	13.81419000	11.10323000	-1.29555900
H	12.72796600	11.38646500	-2.66325400

Table S4. Cartesian coordinates for DFT optimized structure (at B3LYP/6-31g (d) level) of PEG_{420}-TQ.

C	1.71528500	-0.18709100	0.17994700
C	0.90085400	-0.29159300	-0.96727200
C	-0.55274900	-0.27903800	-0.90637300
C	-1.26629300	-0.12133900	0.30039100
C	-0.45465900	0.00356200	1.46542900
C	1.00619900	-0.04470400	1.40821600
N	-0.92687200	0.14759200	2.71884600
S	0.37288600	0.23278000	3.70430000
N	1.58392900	0.08660600	2.61809500
C	9.67321500	3.15993900	-0.99670500
C	10.01237900	1.89631900	-1.50138300
C	9.27587800	0.77590600	-1.14333600
C	8.19176600	0.88203600	-0.25487900
C	7.86888200	2.14464000	0.25480500
C	8.59091400	3.28019400	-0.11848500
N	7.45160800	-0.27522100	0.12475600
C	8.16216600	-1.44216800	0.52898100
C	6.03966100	-0.25401400	0.13434800

C	7.80261700	-2.70956400	0.03693000
C	8.49928400	-3.84504900	0.42517400
C	9.59138700	-3.74440100	1.29942400
C	9.96737000	-2.48789300	1.78529100
C	9.24711100	-1.35247100	1.40762900
C	5.30973000	-0.98837800	1.08587800
C	3.92102400	-0.96155000	1.08987600
C	3.19247600	-0.21128800	0.14752700
C	3.93311600	0.52072000	-0.80099700
C	5.32121900	0.50348000	-0.80945400
O	10.21751900	-4.92045300	1.60995000
O	10.45393700	4.20075600	-1.41947200
O	-9.93561500	-4.75942000	0.52556300
O	-9.66092300	4.89049000	1.36350000
C	-4.77897900	0.89339200	1.30731400
C	-3.39487300	0.85500800	1.20020400
C	-2.74110500	-0.09140400	0.38838000
C	-3.55188600	-1.00760600	-0.30923800
C	-4.93607900	-0.97951400	-0.20465400
C	-7.36802700	2.36357300	0.11760000
C	-8.03980500	3.56362200	0.30338800
C	-9.05235200	3.66894600	1.26793600
C	-9.37374500	2.55430600	2.04961800
C	-8.67770300	1.35673800	1.87199600
C	-5.57962000	-0.02581100	0.60581300
C	-7.67243800	1.23979800	0.90604400
N	-6.98705700	0.00556900	0.71256000
C	-9.65758600	-2.43995000	-0.21820000
C	-8.89179400	-1.27359100	-0.16044700
C	-7.74617200	-1.19843000	0.63893600
C	-7.37257200	-2.33204700	1.38216700
C	-8.11583500	-3.50196000	1.31263100

C	-9.26787800	-3.56543100	0.51546800
N	1.50772400	-0.38358000	-2.19119700
C	0.80281700	-0.48862700	-3.29071500
C	-0.65323200	-0.59795800	-3.21612900
N	-1.26171200	-0.45231700	-2.06487500
C	-1.53374000	-0.92443200	-4.37217100
C	1.57933800	-0.43230200	-4.56040700
C	1.12933200	0.29534500	-5.67426500
C	1.91741900	0.39403600	-6.82002600
C	3.15996100	-0.24001400	-6.87629500
C	3.61903000	-0.96096900	-5.77133300
C	2.84077800	-1.04787500	-4.61927600
C	-2.80169400	-0.32557100	-4.45752600
C	-3.67369600	-0.65607500	-5.49233700
C	-3.30256700	-1.60581200	-6.44718200
C	-2.05346000	-2.22306400	-6.35872300
C	-1.17232300	-1.88255400	-5.33342300
C	11.32692700	-4.88536700	2.49665100
C	11.80774900	-6.32068200	2.65292100
O	12.91315800	-6.29482700	3.53256900
C	13.46648700	-7.57587300	3.77167900
C	14.63716700	-7.39977800	4.72764800
O	15.18790000	-8.67895000	4.96393900
C	16.29018800	-8.64524500	5.84466700
C	-10.69002900	5.06283600	2.32760300
C	10.16188700	5.50651200	-0.94173000
C	-11.10632900	-4.88948200	-0.26846200
C	-11.16975100	6.50160500	2.20287100
O	-12.19672800	6.68121800	3.15649600
C	-12.73954700	7.98880800	3.15263700
C	-13.82409200	8.03962100	4.21873200
O	-14.36482800	9.34458500	4.21158300

C	-15.38869200	9.51726200	5.16751200
C	-11.62212000	-6.30575300	-0.05897200
O	-12.78801000	-6.44354800	-0.84526200
C	-13.37989800	-7.72585900	-0.74611100
C	-14.61314700	-7.73303000	-1.63741400
O	-15.20238400	-9.01282500	-1.53599800
C	-16.36456300	-9.14365400	-2.32596300
C	11.17798700	6.44293500	-1.57923500
O	10.89599500	7.74466100	-1.10785300
C	11.77266800	8.72825400	-1.62585300
C	11.35570500	10.06963500	-1.04085800
O	12.23234600	11.04894200	-1.55850400
C	11.94317700	12.34655900	-1.08469400
H	10.85085800	1.81810700	-2.18653100
H	9.53896900	-0.19620000	-1.54855600
H	7.03853100	2.24352400	0.94699400
H	8.30895000	4.24265900	0.29304200
H	6.96749000	-2.79687600	-0.65098600
H	8.22346100	-4.82522100	0.04864100
H	10.80491900	-2.37890000	2.46488500
H	9.53738600	-0.38197300	1.79777100
H	5.83692100	-1.57706900	1.82908800
H	3.39059800	-1.53185400	1.84370300
H	3.41102800	1.10658800	-1.54701200
H	5.85925100	1.07956700	-1.55489200
H	-5.24721400	1.63620100	1.94437600
H	-2.80822200	1.57243500	1.76234600
H	-3.08905200	-1.74886700	-0.94850700
H	-5.52911300	-1.70064800	-0.75716100
H	-6.59491100	2.28885000	-0.64070200
H	-7.80610900	4.43383800	-0.30217800
H	-10.14889200	2.60645200	2.80570700

H	-8.92426300	0.49903800	2.49002700
H	-10.54133900	-2.46014400	-0.84574200
H	-9.19347600	-0.40725000	-0.74093300
H	-6.49006700	-2.28912900	2.01276000
H	-7.82955900	-4.37979600	1.88358700
H	0.16832800	0.79788000	-5.64038100
H	1.55958000	0.97008200	-7.66912400
H	3.76905100	-0.16854300	-7.77351400
H	4.58779100	-1.45232700	-5.80495700
H	3.20068700	-1.58525500	-3.74809200
H	-3.09195200	0.39286100	-3.69799500
H	-4.64665900	-0.17542500	-5.55070900
H	-3.98455900	-1.86688500	-7.25207100
H	-1.76299000	-2.97443800	-7.08803700
H	-0.20697700	-2.37396000	-5.27166300
H	12.13539600	-4.26180500	2.09129900
H	11.03620000	-4.47931700	3.47501100
H	10.99374900	-6.94585300	3.05316600
H	12.08980200	-6.72925100	1.66944400
H	12.72004700	-8.25214400	4.21756700
H	13.81704000	-8.03645300	2.83447400
H	15.38240000	-6.72050700	4.28172000
H	14.28564600	-6.93724300	5.66471400
H	16.64098800	-9.67390600	5.96186400
H	17.11251900	-8.02980100	5.44532100
H	16.00998900	-8.24763400	6.83352800
H	-11.52410900	4.37274300	2.14088700
H	-10.31279700	4.88049200	3.34299500
H	9.14448500	5.81028200	-1.22315600
H	10.24507600	5.55317100	0.15259500
H	-11.87202000	-4.16333900	0.03690100
H	-10.88137200	-4.72641300	-1.33121400

H	-10.33094400	7.19220600	2.38478600
H	-11.53768000	6.68521900	1.18079100
H	-11.96436800	8.73944100	3.37391100
H	-13.17182300	8.23463100	2.16973600
H	-14.59782400	7.28572500	3.99821800
H	-13.39087400	7.79239900	5.20198600
H	-15.73810000	10.54959200	5.08300000
H	-16.23585000	8.83643100	4.98512000
H	-15.02247000	9.34598100	6.19268300
H	-10.85039700	-7.03252100	-0.35870600
H	-11.83894200	-6.47019900	1.00855700
H	-12.67973300	-8.51094900	-1.07279000
H	-13.67008100	-7.94821200	0.29297900
H	-15.31197800	-6.94490900	-1.31159500
H	-14.32190400	-7.50895200	-2.67693000
H	-16.74135900	-10.15931100	-2.17968500
H	-17.14472700	-8.42559200	-2.02561000
H	-16.14935800	-8.99225200	-3.39611800
H	12.19798000	6.13090100	-1.30355400
H	11.09778600	6.38976500	-2.67669500
H	12.81717900	8.51092000	-1.35169000
H	11.71712500	8.76801000	-2.72525900
H	10.30971400	10.28556200	-1.31442800
H	11.40884500	10.02799200	0.05961900
H	12.66989900	13.02517700	-1.53890100
H	10.92775000	12.66732900	-1.36832700
H	12.03145700	12.40926400	0.01199700

References

1. Bishnoi S, Rehman S, Dutta SB, De SK, Chakraborty A, Nayak D, et al. Optical-property-enhancing novel near-infrared active niosome nanoformulation for deep-tissue bioimaging. ACS Omega. 2021; 6: 22616-24.
2. Zhao D, Tao W, Li S, Chen Y, Sun Y, He Z, et al. Apoptotic body-mediated intercellular delivery for enhanced drug penetration and whole tumor destruction. Sci Adv. 2021; 7: eabg0880.
3. Helft J, Böttcher J, Chakravarty P, Zelenay S, Huotari J, Schraml BU, et al. GM-CSF mouse bone marrow cultures comprise a heterogeneous population of $CD11c^{+}MHCII^{+}$ macrophages and dendritic cells. Immunity. 2015; 42: 1197-211.
4. Toda G, Yamauchi T, Kadowaki T, Ueki K. Preparation and culture of bone marrow-derived macrophages from mice for functional analysis. STAR Protoc. 2021; 2: 100246.
5. Li H, Zhu W, Li M, Li Y, Kwok RTK, Lam JWY, et al. Side area-assisted 3D evaporator with antibiofouling function for ultra-efficient solar steam generation. Adv Mater. 2021; 33: e2102258.
6. Cheng T, Zhao Y, Li X, Lin F, Xu Y, Zhang X, et al. Computation of octanol-water partition coefficients by guiding an additive model with knowledge. Chem Inf Model. 2007; 47: 2140-8.
